# The Role of α-CTD in the Genome-Wide Transcriptional Regulation of the *Bacillus subtilis* Cells

**DOI:** 10.1371/journal.pone.0131588

**Published:** 2015-07-08

**Authors:** Satohiko Murayama, Shu Ishikawa, Onuma Chumsakul, Naotake Ogasawara, Taku Oshima

**Affiliations:** Graduate School of Biological Sciences, Nara Institute of Science and Technology, 8916–5, Takayama, Ikoma, Nara 630–0192, Japan; Indian Institute of Science, INDIA

## Abstract

The amino acid sequence of the RNA polymerase (RNAP) α-subunit is well conserved throughout the Eubacteria. Its C-terminal domain (α-CTD) is important for the transcriptional regulation of specific promoters in both *Escherichia coli* and *Bacillus subtilis*, through interactions with transcription factors and/or a DNA element called the “UP element”. However, there is only limited information regarding the α-CTD regulated genes in *B*. *subtilis* and the importance of this subunit in the transcriptional regulation of *B*. *subtilis*. Here, we established strains and the growth conditions in which the α-subunit of RNAP was replaced with a C-terminally truncated version. Transcriptomic and ChAP-chip analyses revealed that α-CTD deficiency reduced the transcription and RNAP binding of genes related to the utilization of secondary carbon sources, transition state responses, and ribosome synthesis. In *E*. *coli*, it is known that α-CTD also contributes to the expression of genes related to the utilization of secondary carbon sources and ribosome synthesis. Our results suggest that the biological importance of α-CTD is conserved in *B*. *subtilis* and *E*. *coli*, but that its specific roles have diversified between these two bacteria.

## Introduction

The bacterial RNA polymerase (RNAP) holoenzyme, which is composed of α (dimer), β, β’ and σ subunits, binds to promoters to initiate transcription [1]. RNAP holoenzymes assembled with the major sigma factors, σ^70^ (*E*. *coli*) or σ^A^ (*B*. *subtilis*), recognize canonical promoters that have two conserved elements, the -10 and -35 elements, in both *E*. *coli* and *B*. *subtilis* [1–4].

The α-subunit of RNAP, encoded by the *rpoA* gene, has two domains: an N-terminal domain (α-NTD) and a C-terminal domain (α-CTD), that are connected by a flexible linker [4–6]. The former is required for dimerization of the α subunit and initiates RNAP assembly [7–11], while the latter supports the efficient recruitment of RNAP to promoters through interactions with AT-rich DNA elements upstream of the -35 elements of promoters, so called “UP elements”, or interactions with transcriptional activators that bind upstream of the -35 elements [1,12–16]. At the amino acid level, the α subunits are well conserved between *B*. *subtilis* and *E*. *coli* (36% identity and 65% similarity) [17]. The α-CTD residues that interact with UP elements are also conserved, suggesting that the mechanism through which transcription is activated by the interaction between the α-subunit of RNAP, and promoters may also be conserved between the two bacteria [4,18].

Studies have suggested that α-CTD is essential for cell viability. In *E*. *coli*, the temperature-sensitive growth caused by mutations in α-NTD was not complemented by the expression of an α-CTD-truncated α-subunit [8,19]. Experiments examining alanine substitutions of various amino acids in the *B*. *subtilis* α-CTD revealed that the G292A and R261A substitutions could not be introduced, suggesting that these residues are essential for the viability of *B*. *subtilis* cells [20]. In *Staphylococcus aureus*, the phage G1 protein, gp67, inhibits the expression of translation-related genes (i.e., those encoding rRNA and ribosomal proteins) by disrupting the interaction of α-CTD with the UP element, potentially explaining the growth defects caused by the expression of gp67 [21]. The *E*. *coli* rRNA gene promoter, *rrn P1*, is also activated in the presence of the UP element and the transcriptional activator, Fis, which enhance the promoter’s basal activity by ~ 300-fold [22]. These results suggest that activation of the rRNA promoter by α-CTD might be essential for the viabilities of *E*. *coli* and *S*. *aureus*. Meanwhile, *B*. *subtilis* rRNA promoters are only weakly dependent on their upstream sequence (enhanced 3 fold) [23].

The interactions of α-CTD with transcriptional regulators (p4 at the A3 promoter of the phage Φ29 and ComA at the *srfA* promoter [17,20]) or UP elements (at the Φ29 early operon promoters, C2, A2b and A2c [24]) in *B*. *subtilis* were demonstrated *in vitro* using α-CTD-deficient reconstituted RNAP. In addition, deletion analysis upstream of a number of promoters (*hag*, *fliD*, *motA* flagellar genes and *spoVG* gene related to spore formation) followed by *in vitro* transctiption and *in vivo lacZ* reporter analysis revealed the ability of AT-rich sequences upstream of the -35 element to enhance their transcriptional activities [25–28]. Thus, UP elements appear to contribute to the activation of a number of promoters in *B*. *subtilis* cells. In contrast to the relatively rich information regarding α-CTD dependent transcriptional regulation in *E*. *coli* [4,7,29], however, we have only limited information on α-CTD-dependent transcriptional regulation in *B*. *subtilis*, or its importance to global transcriptional regulation in this organism.

The aim of this study was to gain new insight into the role of α-CTD in the transcriptional regulation of *B*. *subtilis*. To this end, we applied genome-wide approaches to identify α-CTD-dependent gene expressions in *B*. *subtilis* cells. We constructed *B*. *subtilis* strains in which the expression of intact and C-terminal-truncated α subunits was induced by different stimuli (IPTG and xylose) under the control of the P*spac* and P*xyl* promoters. We then used transcriptome and ChAP-chip analyses to examine the impact of a defective α-CTD on genome-wide transcription. Our results revealed that α-CTD deficiency primarily down-regulated the transcription of genes related to the transition-state response, the utilization of secondary carbon sources, and the synthesis of ribosomal proteins. Notably, a number of genes found to be down-regulated by α-CTD deficiency contained transcriptional activator binding sites and UP elements upstream of the -35 elements of their promoters. Furthermore, the ChAP-chip results identified two types of promoters for which α-CTD deficiency inhibited the recruitment or the productive complex formation of RNAP. Thus, our results demonstrate that although α-CTD contributes to the same biological activities (i.e., the utilization of secondary carbon sources and ribosomal synthesis) in *B*. *subtilis* and *E*. *coli*, its specific roles have diversified between these two bacteria.

## Results and Discussion

### Construction of strains to investigate α-CTD activity *in vivo*

**Fig 1:**
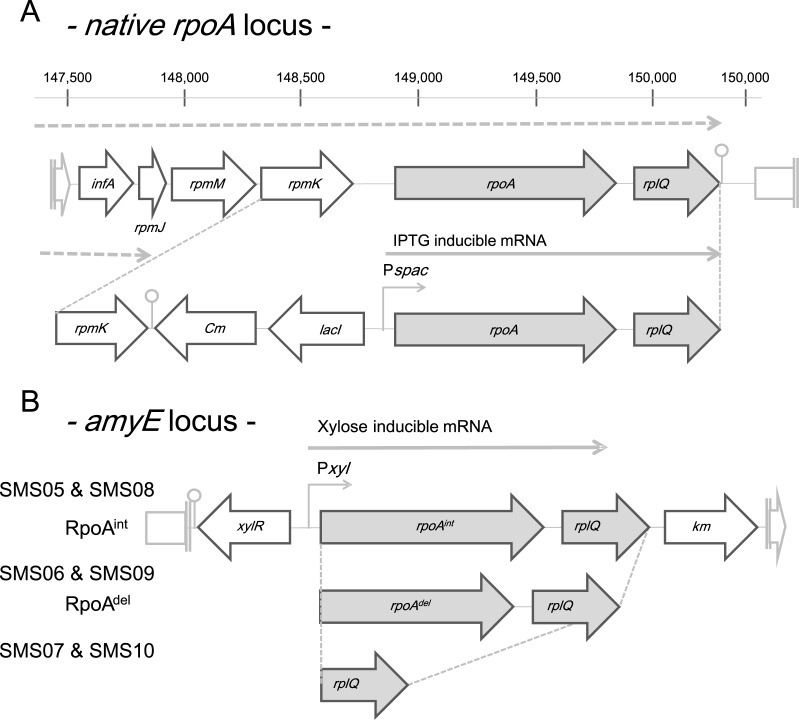
Schematic representation of the loci used to switch *rpoA* expression from IPTG-to xylose-inducible. Open thick arrows represent genes, while thick gray arrows represent genes whose expression levels were induced by IPTG or xylose via the P*spac* and P*xyl* promoters, respectively. The dashed line (with arrowhead) represents the region in which transcription was initiated from the original promoter of the operon; this region includes *rpoA* and *rplQ*, which are expected to be located upstream of *infA* [31]. Gray solid lines represent the transcribed regions of the new transcriptional units (P*spac*-*rpoA* ^*int*^ *-rplQ*, P*xyl*-*rpoA* ^*int*^ *-rplQ*, P*xyl*-*rpoA* ^*del*^ *-rplQ* or P*xyl*-*rplQ*) regulated by the P*spac* and P*xyl* promoters. Native *rpoA* locus (A) and *amyE* locus (B) are shown.

To examine the *in vivo* activities of α-CTD, we constructed strains in which the expression of RpoA (we use “RpoA” to indicate the product of the *rpoA* gene, instead of “α-subunit") could be switched from an intact RpoA (RpoA^int^) to a C-terminally truncated RpoA (RpoA^del^). RpoA^del^ lacked the folded domain of α-CTD, which is composed of four α-helices(α1 ~ α4, 66 amino acids), while retaining the four amino acids at the C-terminal end (RKDD, outside of the folded domain) (S1 Fig) [30]. The expressions of RpoA^int^ and RpoA^del^ were respectively controlled by the inducible promoters, P*spac*, which is repressed by the LacI repressor and activated by IPTG (isopropyl β-D-1-thiogalactopyranoside), and P*xyl* which is repressed by the XylR repressor and activated by xylose (Fig 1). The *rpoA* gene is co-transcribed with *infA* (encoding a translation initiation factor), *rpmJ*, *rpmM*, *rpmK* and *rplQ* (encoding ribosome subunits) (Fig 1A) [31]. The transcription of this transcriptional unit (TU) initiates upstream of *infA*, while the transcriptional terminator follows after the termination codon of *rplQ* [31]. We introduced the P*spac* promoter with *lacI* (encoding LacI) into the intergenic region between *rpoA* and *rpmK* on the *B*. *subtilis* chromosome (Fig 1A), thus, terminating transcription of *infA* upstream of *rpoA* and putting the expression of *rpoA* and *rplQ* under the control of the P*spac* promoter. In addition, a DNA fragment containing the P*xyl* promoter followed by the genes encoding RpoA^int^ or RpoA^del^ and *xylR* (encoding XylR) was introduced into the *amyE* locus of the *B*. *subtilis* chromosome (Fig 1B). The *rplQ* gene is essential for *B*. *subtilis* cell viability [31]. The expressions of *rpoA* and *rplQ* via the P*spac* promoter were simultaneously repressed by LacI when our constructed strain was grown in LB medium without IPTG. To compensate for the depletion of *rplQ*, we introduced *rplQ* downstream of the P*xyl* promoter and *rpoA* into the *amyE* locus. Our constructed strain, which harbored an IPTG-inducible intact *rpoA* (*rpoA* ^*int*^) gene and a xylose-inducible truncated *rpoA* gene (*rpoA* ^*del*^), was unable to grow on the LB plate containing 1% xylose and lacking IPTG, whereas the strain harboring xylose-inducible *rpoA* ^int^ (instead of *rpoA* ^*del*^) at the *amyE* locus was viable on the same LB plate (S2 Fig). This confirms that α-CTD is essential for the cell viability of *B*. *subtilis*.

We designated the constructed strains as SMS05 (P*spac*-*rpoA* ^int^-*rplQ*, P*xyl*-*rpoA* ^int^ *-rplQ*), SMS06 (P*spac*-*rpoA* ^int^-*rplQ*, P*xyl*-*rpoA* ^del^-*rplQ*) and SMS07 (P*spac*-*rpoA* ^int^-*rplQ*, P*xyl*-*rplQ*) and used this system to examine the function of α-CTD in *B*. *subtilis*.

### Growth of cells following the induction of RpoA^del^

**Fig 2:**
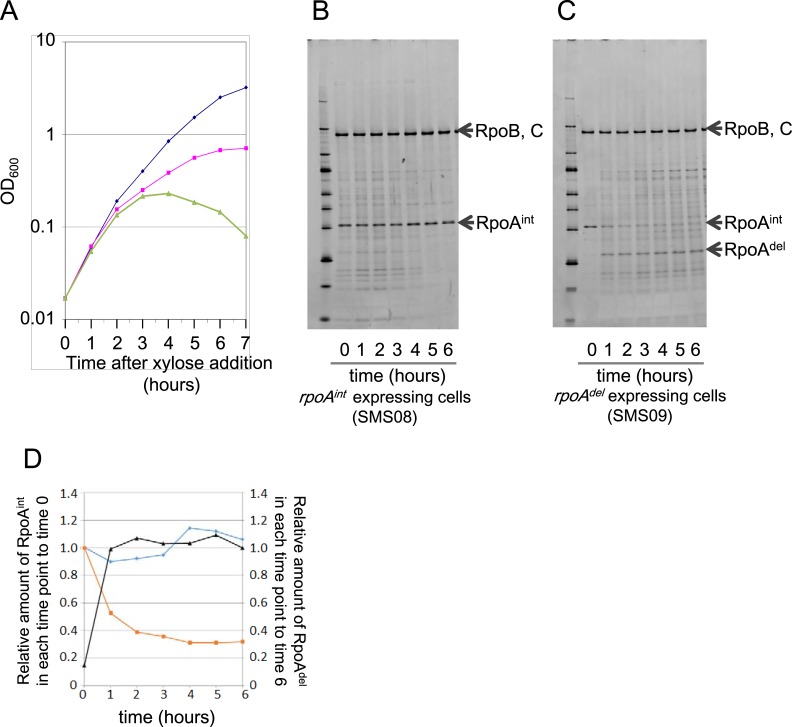
The effects of replacing RpoA^int^ with RpoA^del^ in the RNAP complexes of *rpoA* ^*del*^-expressing cells. (A) Growth curves of strains that expressed RpoA^int^ (SMS05, blue line), RpoA^del^ (SMS06, pink line) and RplQ without RpoA (SMS07, green line), upon xylose induction. (B and C) Pull-down assays of RpoA assembled in RNAP complexes. RNAP complexes were purified using His-tagged RpoC as bait, separated by SDS-PAGE and detected by fluorescent staining with Flamingo dye (Bio-Rad), which is characterized by a high sensitivity and a low background, allowing accurate quantification [32]. The band positions corresponding to RpoB and C, RpoA^int^ and RpoA^del^ in the SDS-PAGE gel are indicated by arrows (right). The duration of cultivation in LB^xyl^ is shown at the bottom of each panel. (D) Time course of alteration in the relative amounts of RpoA^int^ and RpoA^del^ in the RNAP complexes of *rpoA* ^*int*^- and *rpoA* ^*del*^-expressing cells grown in LB^xyl^. The blue line indicates the relative ratio (see below) of the amount of RpoA^int^ in the RNAP complexes of *rpoA* ^*int*^-expressing cells at each time point. For normalization, the amount of RpoA^int^ was divided by that of RpoB, C in each lane as follows: normalized signal intensity of RpoA = [signal intensity of the RpoA band] / [signal intensity of the RpoB, C band]. The amount of RpoA^int^ at each time point was then divided by the amount of RpoA^int^ at time 0 as follows: relative ratio (RpoA^int (X hour)^) = [normalized signal intensity of RpoA^int (X hour)^] / [normalized signal intensity of RpoA^int (0 hour)^]. The orange line represents the relative ratios of RpoA^int^ in the RNAP complexes of *rpoA* ^*del*^-expressing cells at each time point, calculated as described above. The black line shows the relative ratios of the amount of RpoA^del^ in the RNAP complexes of *rpoA* ^*del*^-expressing cells at each time point against to the amount of RpoA^del^ at 6 hours after the beginning of the RpoA^del^ induction. Here, the relative ratio was calculated as: relative ratio (RpoA^del (X hour)^) = [normalized signal intensity of RpoA^del (X hour)^] / [normalized signal intensity of RpoA^del (6 hour)^]. Each relative ratio was calculated using the average values obtained from triplicate experiments.

We first examined the effects of the RpoA^del^ expression/RpoA^int^ depletion on the cell growth. *B*. *subtilis* cells were cultivated to log phase in LB medium containing 1 mM IPTG (LB^IPTG^), to trigger the expression of RpoA^int^. The cells were, then, washed with LB medium to remove the IPTG, and re-cultivated in LB medium containing 1% xylose (LB^xyl^). The growth curves of the *rpoA* ^*int*^- and the *rpoA* ^*del*^-expressing strains in LB^xyl^ are shown in Fig 2A. After the induction of *rpoA* ^*del*^ expression, the growth of both strains continued for at least 6 hours. There was little difference in the growth rates during the initial 2 hours of the cultivation in LB^xyl^ (Fig 2A, blue and pink lines). However, the growth rate of *rpoA* ^*del*^-expressing cells over the total 6-hour cultivation (Fig 2A, pink line) was significantly reduced, compared with that of *rpoA* ^*int*^-expressing cells (Fig 2A, blue line); the doubling time of *rpoA* ^*del*^ expressing cells was 1.9 hours, while that of the *rpoA* ^*int*^-expressing cells was 1.0 hour. Meanwhile, SMS07, in which *rplQ* was expressed, but RpoA was depleted, showed growth arrest in LB^xyl^ (Fig 2A, green line), suggesting that *rpoA* ^*del*^-expressing cells had sufficient RNAP activity to maintain cell growth for several hours.

### In the RNAP complex, RpoA^int^ is replaced by RpoA^del^ in *rpoA* ^*del*^-expressing cells

In order to compare the activity of RpoA^del^ with that of RpoA^int^ in transcriptional regulation, the expressed RpoA^del^ must be correctly assembled into the RNAP complex. To test whether this was the case, we constructed strains in which RpoC (the β’ subunit of RNAP) was expressed with a C-terminal histidine-tag. We have demonstrated that the C-terminal histidine-tagged RpoC (RpoC-His) correctly assembled with other RNAP subunits and formed competent RNAP complex, which could interact with the elongation factors GreA and NusA, and showed elongation activity [33]. In addition, the *B*.*subtilis* cells expressing the tagged RpoC showed normal growth [34]. These cells were designated SMS08 (P*spac*-*rpoA* ^int^-*rplQ*, P*xyl*-*rpoA* ^int^ *-rplQ*, *rpoC*-His12) and SMS09 (P*spac*-*rpoA* ^int^-*rplQ*, P*xyl*-*rpoA* ^del^-*rplQ*, *rpoC*-His12).

Western blotting using the anti-RpoB antibody and crude extracts prepared from *rpoA* ^*int*^- and *rpoA* ^*del*^-expressing cells indicated that the amount of RNAP complex was constant in both cell types during the 6 hours of growth in LB^xyl^ (S3 Fig). This result indicated that α-CTD deficiency did not affect the amount of RNAP complex in *B*. *subtilis* cells grown under our experimental conditions. The RNAP complexes were affinity purified via the C-terminal histidine-tag of RpoC (RpoC-His) and resolved by SDS-PAGE. To evaluate alterations in the amount of RpoA^int^ and RpoA^del^ assembled into the RNAP complex, the amounts of RpoA^int^ were determined at each time point relative to that at the beginning of the RpoA^del^ induction (0 hour), while those of RpoA^del^ at each time point were determined relative to that at the end of the 6-hour study period (Fig 2B and 2C). Our results revealed that the relative amount of RpoA^int^ in the RNAP complexes of *rpoA* ^*int*^-expressing cells was constant during the 6-hour cultivation in LB^xyl^ (Fig 2D, blue line), while the relative amount of RpoA^int^ in the RNAP complexes of *rpoA* ^*del*^-expressing cells gradually decreased to about 30% of the initial amount (0 hour) over the first 3 hours of cultivation in LB^xyl^ and more moderately decreased thereafter (Fig 2D, orange line). This is consistent with the slow turnover of RpoA in RNAP complexes, as previously observed in *E*. *coli* cells [8]. In parallel with this reduction in RpoA^int^, the amount of RpoA^del^ assembled in the RNAP complexes increased between 0 and 3 hours of cultivation in this system (Fig 2D, black line). It is possible that the remaining signal at the RpoA^int^ position after 3 hours of cultivation reflected background noise. Regardless, our results indicate that at least 70% of the RpoA^int^ assembled into RNAP complexes was replaced by RpoA^del^ in *rpoA* ^*del*^-expressing cells cultivated in LB^xyl^.

### RpoA^del^-containing RNAP complex can bind DNA and form elongation complexes in *B*. *subtilis*

We next questioned whether RNAP assembled with RpoA^del^ lost DNA-binding activity. We used ChAP-chip analysis of RpoC and RpoA to evaluate the DNA-binding activity of the RpoA^del^-containing RNAP toward the *B*. *subtilis* chromosome [34]. In addition to the RpoC-His-expressing strains, we constructed strains expressing RpoA with a C-terminal histidine tag (RpoA^int^-His and RpoA^del^-His) and designated them SMS18 (P*spac-rpoA* ^*int*^ *-rplQ*, Pxyl-*rpoA* ^*int*^-His-*rplQ*) and SMS19 (P*spac-rpoA* ^*int*^-*rplQ*, P*xyl-rpoA* ^*del*^-His-*rplQ*).

Given the above results reflecting the replacement of RpoA^int^ with RpoA^del^ in the RNAP complexes of *rpoA* ^*del*^-expressing cells (SMS09) cultivated for 3 hours in LB^xyl^, cells were cultured for 3 hours in LB^xyl^ and then treated with formaldehyde to crosslink the chromosomal DNA and RNAP. ChAP-chip analyses of RpoC-His and RpoA-His were performed as described previously [33,34].

Because there are multiple and highly homologous rRNA and tRNA genes, raising the possibility that cross hybridization could prevent the accurate quantification of their RNAP bindings, we compared the RNAP binding intensities with respect to protein-coding regions on *B*. *subtilis* genomic DNA in the *rpoA* ^*del*^- and *rpoA* ^*int*^-expressing cells. The RNAP (RpoC-His) binding intensities in the *rpoA* ^*del*^ (SMS09) and *rpoA* ^*int*^ (SMS08)-expressing cells were positively correlated (S4A Fig, *r* > 0.90), as were the binding intensities of RpoA^del^-His and RpoA^int^-His in the *rpoA* ^*del*^ (SMS19) and *rpoA* ^*int*^-expressing cells (SMS18) (S5 Fig, *r* > 0.96). These results clearly indicated that the RNAP complex assembled with RpoA^del^ could bind to the *B*. *subtilis* chromosome and form an elongation complex. Detailed analysis of the ChAP-chip results discussed later.

### The impact of α-CTD deficiency on the *B*. *subtilis* transcriptome

To explore the impact of α-CTD deficiency on global transcriptional gene regulation in *B*. *subtilis*, the transcriptomes of *rpoA* ^*int*^-expressing cells (SMS08) and *rpoA* ^*del*^-expressing cells (SMS09) were compared using the Affymetrix Genechip *B*. *subtilis* genome array (Affymetrix). We used the same growth conditions used in the ChAP-chip analysis and determined the transcriptomes of *rpoA* ^*int*^- and *rpoA* ^*del*^-expressing cells grown in LB^xyl^ for 0, 1, 2 and 3 hours.

**Fig 3:**
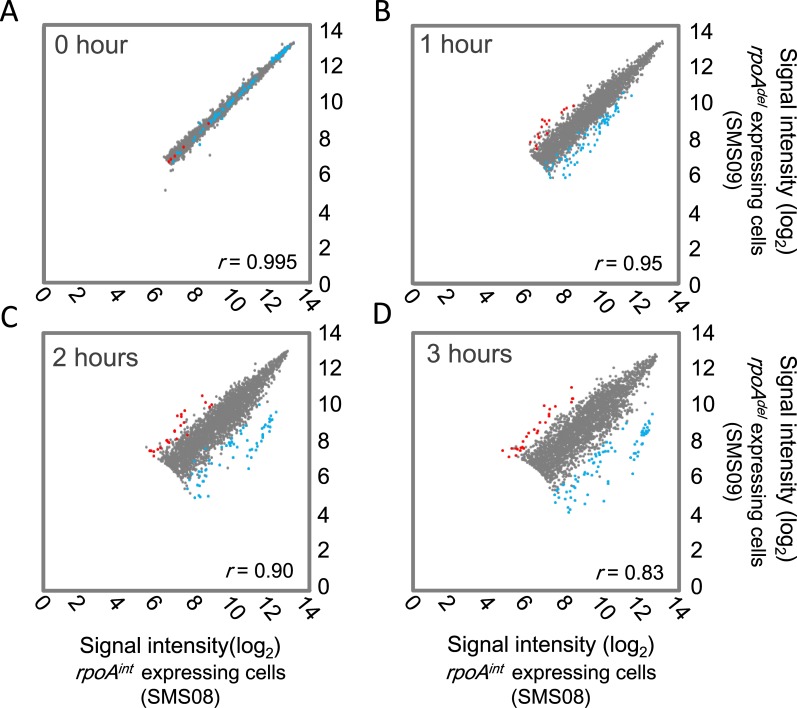
Transcriptome analysis of *rpoA* ^*int*^-expressing cells (SMS08) and *rpoA* ^*del*^-expressing cells (SMS09). A scatter plot (log_2_ scale) of the transcriptional signal intensity (averaged from duplicate experiments) of each gene in *rpoA* ^*del*^-expressing cells (vertical axis) versus *rpoA* ^*int*^-expressing cells (horizontal axis) at 0 hour (A) and at 1 hour (B), 2 hours (C) and 3 hours (D) after the beginning of the RpoA^del^ induction. The correlation coefficients between the transcriptomes of *rpoA* ^*int*^ and *rpoA* ^*del*^-expressing cells are indicated as (*r*) in each panel. The average signal intensities from two independent experiments are plotted. For each gene plotted, the sum of the signal intensities for all experiments performed at the same time point (two experiments each for *rpoA* ^*int*^- and *rpoA* ^*del*^-expressing cells) was > 400; this avoided the inclusion of minimally expressed genes in our analysis. We analyzed a total of 2755 (0 hour), 2855 (1 hour), 2921 (2 hour) and 2956 (3 hour) genes. The genes found to be down-regulated in *rpoA* ^*del*^-expressing cells compared to *rpoA* ^*int*^-expressing cells at 3 hours after the beginning of the RpoA^del^ induction are shown as blue dots, and the up-regulated genes are shown by red dots.

The differences between the transcriptomes of *rpoA* ^*int*^-expressing cells and *rpoA* ^*del*^-expressing cells gradually increased during their cultivation in LB^xyl^. The correlation coefficient, therefore, gradually decreased over the same period (Fig 3). To identify the genes whose expression levels were affected by α-CTD deficiency, we extracted data for genes whose expression levels differed by > 4- or < -0.25 fold (> 2 or < -2 in log_2_ scale) in the *rpoA* ^*del*^-expressing cells compared to the *rpoA* ^*int*^-expressing cells and which had a false discovery rate (*q*-value) of < 0.2. After 3 hours cultivation, we identified 53 down-regulated genes and 27 up-regulated genes differentially expressed between the *rpoA* ^*int*^-expressing cells (SMS08) and the *rpoA* ^*del*^-expressing cells (SMS09) (S5 Table). Amongst them, there were 20 down- and 5 up-regulated genes that were also differentially expressed after 2 hours of cultivation (S2 Table). At 2 hours, we identified four additional down-regulated genes (*yhfH*, *yhzC*, *ykbA* and *yokG*) and one additional up-regulated gene (*ybcP*) in the *rpoA* ^*del*^-expressing cells. At 3 hours, these genes were excluded from the list of differentially expressed genes for the following reasons: *yhzC* showed a low expression level; *yhfH* had a high *q*-value (log_2_ fold = -4.13, *q* = 0.201); *ykbA* and *yokG* had moderate fold changes or a high *q*-value (*ykbA*, log_2_ fold = -1.95, *q* = 0.14; *yokG*, log_2_ fold = -2.47, *q* = 0.24); and *ybcP* had a high *q*-value (log_2_ fold = 2.64, *q* = 0.24). In general, however, the up- and down-regulated genes overlapped at 2 and 3 hours. At 0 and 1 hour, no up- or down-regulation was identified by the criteria used in this study. We detected the known down-regulation of the *srf* operon that was previously associated with α-CTD deficiency [20], but not the known down-regulation of flagellar genes, *hag*, *fliD* and *motA* [25,26]. These results might indicate that the transcriptional regulation of *hag*, *fliD* and *motA* have more factors affecting them than simple UP element dependent regulation.

### α-CTD deficiency increases the transcription of genes with alternative σ factor-dependent promoters

**Fig 4:**
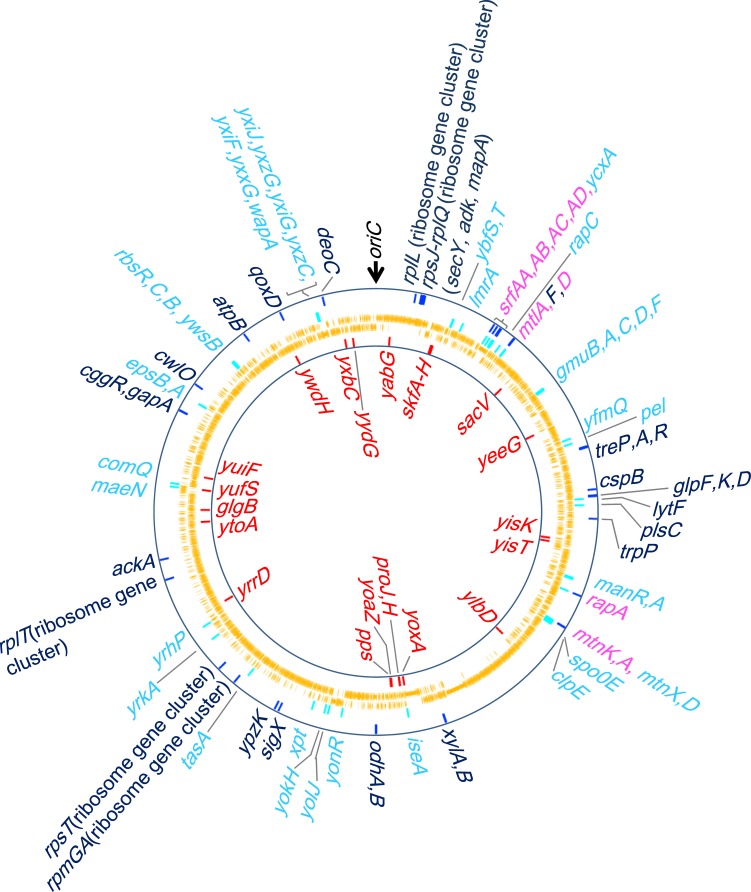
Genome-wide map of the genes up- and down-regulated, and highly reduced RNAP binding in *rpoA* ^*del*^-expressing cells (at 3 hours). From the outermost ring, the genes with highly reduced RNAP binding identified by ChAP-chip analysis (dark-blue bars, gene names corresponding to those bars are indicated by dark-blue letters outside of rings), down-regulated genes identified by transcriptome analysis (sky-blue bars, gene names corresponding to those bars are indicated by sky-blue letters outside of rings), CDSs (orange bars, an outer and inner ring indicates clockwise and counterclockwise CDSs, respectively) and up-regulated genes identified by transcriptome analysis (red bars in innermost ring, gene names corresponding to those bars are indicated by red letters) are shown as bars at their corresponding genome coordinates. The pink letters indicate the genes which were identified as the genes both down-regulated and with highly reduced RNAP binding. The GenomeMatcher was used to draw rings [40].

In the DBTBS and BsubCyc databases [35,36], there is information about promoters for nine of the genes found to be up-regulated in *rpoA* ^*del*^-expressing cells. Of them, six are known to interact with alternative σ factors, while the other three are known to be σ^A^ dependent (Fig 4 and S1 Table). Therefore, some of the up-regulation caused by α-CTD deficiency might be explained by the activation of alternative σ factors. However, no unique alternative σ factor was found to be responsible for up-regulation in *rpoA* ^*del*^-expressing cells (S1 Table), making it impossible to estimate the stresses and environmental signals required to activate these promoters under α-CTD deficiency. In addition, the three up-regulated TUs found to have σ^A^-dependent promoters were also differed from each other in terms of their functions; the *skf* operon is involved in the transition state response [37]; *proH-proJ* is involved in stress response [38]; and the *yydF* operon is involved in cell wall metabolism [39]. Hence, we did not estimate the potential biological relevance of the transcriptional up-regulation by α-CTD deficiency. In addition, the up-regulation induced by α-CTD deficiency was relatively weaker than the corresponding down-regulation (Fig 3C and 3D). Hence, we focused the following analyses on the genes and transcriptional units found to be down-regulated in α-CTD deficient cells.

### Functions and transcriptional regulation of the genes down-regulated under α-CTD deficiency

To examine the potential biological relevance of the α-CTD-dependent activation of gene expression in *B*. *subtilis* cells, we investigated the functions of the 53 genes found to be down-regulated after 3 hours of growth in LB^xyl^ (Fig 4 and Table 1, in which the result of ChAP-chip is also summarized [the ChAP-chip result is discussed below]). The functions and other data regarding these down-regulated genes and transcriptional units (TUs) are presented in Table 2. Interestingly, we identified a large proportion of genes known to be up-regulated in response to changes in nutrient availability. More than half of the genes were related to the transition-state response (13 genes), including the quorum-sensing-related genes (*srfAA*, *AB*, *AC*, *AD* [*srf* operon], *rapA*, *rapC and pel*) [41,42], and biofilm-formation-related genes (*epsB*, *A* and *tasA*) [43], or the utilization of secondary carbon sources (16 genes), including genes related to the utilization of phosphoenolpyruvate-carbohydrate phosphotransferase system (PTS) sugars and other carbon sources (*rbsR*, *C*, *B* [the *rbs* operon], *gmuB*, *A*, *C*, *D*, *F* [the *gmu* operon], *gamA*, *P*, *manP*, *A*, *manR* and *mtlA*, *D*) [44–49] (Fig 4 and Table 2). These results strongly suggest that α-CTD plays an important role in the activation of genes involved in the adaptation to nutrient availability.

**Table 1:** Functional categories of the genes that showed down-regulation and/or highly reduced RNAP binding in *rpoA* ^*del*^-expressing cells.

Functional category	Transcriptome^a^	ChAP-chip^b^	Overlap^c^	Total^d^
Antibiotics related	2(2)			2(2)
Cell wall metabolism	3(2)	1(1)		4(3)
Membrane synthesis	1(1)			1(1)
Methionine salvage	4(2)	2(1)	2(1)	4(2)
Purine/pyrimidine metabolism	1(1)	1(1)		2(2)
Stress response	1(1)	2(2)		3(3)
Transition state response	13(8)	5(2)	5(2)	13(8)
Utilization of secondary carbon sources	16(7)	17(9)	2(1)	31(15)
Energy production	0(0)	4(3)		4(3)
Ribosomal synthesis	0(0)	17(5)		17(5)
Undefined	12(12)	1(1)		13(13)
Total	53(36)	50(25)	9(4)	94(57)

^a^: Number of genes and TUs (in parentheses) down-regulated in *rpoA* ^*del*^-expressing cells, as identified by transcriptome analysis.

^b^: Number of genes and TUs (in parentheses) that showed highly reduced RNAP binding in *rpoA* ^*del*^-expressing cells, as identified by ChAP-chip analysis.

^c^: Number of genes detected as being altered by both transcriptome and ChAP-chip analyses.

^d^: Total number of genes and TUs in each functional category.

**Table 2:** Summary of the genes identified by transcriptomic analysis as being down-regulated in *rpoA* ^*del*^-expressing cells.

TUs^a^	TFs^b^	Functional category	Gene	Function and/or encoded protein^c^
*srfAA-srfAB-comS-srfAC-srfAD*	ComA, PerR, CodY	Transition state response	*srfAA*	Surfactin synthase subunit 1
			*srfAB*	Surfactin synthase subunit 2
			*comS*	Competence protein S
			*srfAC*	Surfactin synthase subunit 3
			*srfAD*	Surfactin synthase thioesterase subunit
*rapA-phrA*	ComA, (Spo0A)	Transition state response	*rapA*	Response regulator aspartate phosphatase A
*rapC-phrC*	ComA, (CodY)	Transition state response	*rapC*	Response regulator aspartate phosphatase C
*pel*	(ComA)	Transition state response	*pel*	Pectate lyase
*comQX*		Transition state response	*comQ*	Competence regulatory protein, ComQ
*spo0E*		Transition state response	*spo0E*	Aspartyl-phosphate phosphatase, Spo0E
*eps*	RemA, SinR	Transition state response	*epsA*	Similar to capsular polysaccharide biosynthesis protein
			*epsB*	Similar to capsular polysaccharide biosynthesis protein
*yqxM-sipW-tasA*	RemA, SinR, AbrB, (Spo0A)	Transition state response	*tasA*	Spore coat-associated protein N
*manPA-yjdF*	ManR	Utilization of secondary carbon sources	*manA*	Mannose-6-phosphate isomerase, ManA
			*manP*	PTS system mannose-specific EIIBCA component
*manR*	ManR	Utilization of secondary carbon sources	*manR*	Probable transcriptional regulator, ManR
*rbsRKDACB*	CcpA, AbrB	Utilization of secondary carbon sources	*rbsC*	Ribose transport system permease protein, RbsC
			*rbsA*	Ribose import ATP-binding protein, RbsA
			*rbsR*	Ribose operon repressor
*mtlAFD*	(MtlR)	Utilization of secondary carbon sources	*mtlA*	PTS system mannitol-specific EIICB component
			*mtlD*	Mannitol-1-phosphate 5-dehydrogenase
*gamAP*	(YgaR)	Utilization of secondary carbon sources	*gamA*	Probable glucosamine-6-phosphate deaminase 2
			*gamP*	Putative PTS system glucosamine-specific EIICBA component
*gmuBACDREFG*	(CcpA), (GmuR)	Utilization of secondary carbon sources	*gmuB*	Oligo-beta-mannoside-specific phosphotransferase enzyme IIB component
			*gmuA*	Oligo-beta-mannoside-specific phosphotransferase enzyme IIA component
			*gmuC*	Oligo-beta-mannoside permease IIC component
			*gmuD*	6-Phospho-beta-glucosidase GmuD
			*gmuF*	Mannose-6-phosphate isomerase
*maeN*	YufM	Utilization of secondary carbon sources	*maeN*	Na(+)-malate symporter
*mtnKA*		Methionine salvage	*mtnA*	Methylthioribose-1-phosphate isomerase
			*mtnK*	Methylthioribose kinase
*wapA-yxxG*	YycF	Cell wall metabolism	*wapA*	Wall-associated protein
			*yxxG*	Uncharacterized protein YxxG
*yoeB*	YycF	Cell wall metabolism	*yoeB*	Uncharacterized protein YoeB
*plsC*		Membrane synthesis	*plsC*	1-Acyl-sn-glycerol-3-phosphate acyltransferase
*mtnWXBD*		Methionine salvage	*mtnX*	2-hydroxy-3-keto-5-methylthiopentenyl-1-phosphate phosphatase
			*mtnD*	Acireductone dioxygenase
*xpt-pbuX*	PurR	Purine/pyrimidine metabolism	*xpt*	Xanthine phosphoribosyltransferase
*lmrAB*	LmrA	Antibiotics related	*lmrA*	HTH-type transcriptional regulator, LmrA
*sunS*		Antibiotics related	*sunS*	SPBc2 prophage-derived glycosyltransferase, SunS
*ywsB*		Undefined	*ywsB*	Cell wall-binding protein, YwsB
*ycxA*		Undefined	*ycxA*	Uncharacterized MFS-type transporter, YcxA
*yokH*		Undefined	*yokH*	SPBc2 prophage-derived uncharacterized protein, YokH
*clpE*	CtsR	Stress response	*clpE*	ATP-dependent Clp protease ATP-binding subunit, ClpE
*yrkA*		Undefined	*yrkA*	UPF0053 protein, YrkA
*yxzC*		Undefined	*yxzC*	Uncharacterized protein, YxzC
*yxiG*		Undefined	*yxiG*	Uncharacterized protein, YxiG
*yonR*		Undefined	*yonR*	SPBc2 prophage-derived uncharacterized HTH-type transcriptional regulator, YonR
*yxiF*		Undefined	*yxiF*	Uncharacterized protein, YxiF
*yrhP*		Undefined	*yrhP*	Uncharacterized membrane protein, YrhP
*yfmQ*		Undefined	*yfmQ*	Uncharacterized protein, YfmQ
*yxzG*		Undefined	*yxzG*	Uncharacterized protein, YxzG
*yxiJ*		Undefined	*yxiJ*	Uncharacterized protein, YxiJ

^a^: Information on the transcriptional units (TUs) was acquired from DBTBS and Bsubcyc. [35, 36]

^b^: Transcription factors (TFs) whose binding sites have been identified near the transcriptional start site of each gene. Parentheses indicate TFs whose involvements have been suggested by genetic analysis (i.e., gene knockout), but whose binding sites not yet been identified.

^c^: Information on gene function was mainly acquired from the Panther database (http://www.pantherdb.org/), while the functions of *epsA* and *epsB* were obtained from the BSORF database (http://bacillus.genome.ad.jp/). The *plsC* gene is essential for the viability of *B*. *subtilis* cells.

### ChAP-chip analysis suggests the down-regulation of genes related to utilization of secondary carbon sources and ribosome synthesis in the *rpoA* ^*del*^-expressing cells

To further investigate the α-CTD-dependent activation of gene expression in *B*. *subtilis* cells, we extracted the genes whose RNAP bindings were highly reduced in *rpoA* ^*del*^-expressing cells compared to *rpoA* ^*int*^-expressing cells, based on our RpoC-His ChAP-chip result. We calculated the relative ratio of the RNAP binding intensities obtained for each gene from the *rpoA* ^*del*^- and *rpoA* ^*int*^-expressing cells and extracted the 50 genes that were found to be most highly reduced in their RNAP binding (≈ top 1% of all genes), based on the rank of genes in order of the level of reduction (the rank is the average of ranks in duplicate experiments, see S3 Table). The genes highly reduced in their RNAP binding are summarized with up- and down-regulated genes identified by transcriptome in Fig 4 and Table 3.

**Table 3:** Summary of the genes that showed highly reduced RNAP binding in the *rpoA* ^*del*^-expressing cells, as identified by ChAP-chip analysis.

TUs^a^	Transcr-iptome^b^	Regulator^c^	Functional category	Gene	Esse-ntial^d^	Function and/or encoded protein ^e^
*srfAA-srfAB-comS-srfAC-srfAD*	Y	ComA, PerR, CodY	Transition state response	*srfAA*		Surfactin synthase subunit 1
	Y			*srfAB*		Surfactin synthase subunit 2
	Y			*srfAC*		Surfactin synthase subunit 3
	Y			*srfAD*		Surfactin synthase thioesterase subunit
*rapA-phrA*	Y	ComA, (Spo0A)	Transition state response	*rapA*		Response regulator aspartate phosphatase A
*mtnKA*	Y		Methionine salvages	*mtnA*		Methylthioribose-1-phosphate isomerase
	Y			*mtnK*		Methylthioribose kinase
*mtlAFD*	Y	(MtlR)	Utilization of secondary carbon sources	*mtlA*		PTS system mannitol-specific EIICB component
	Y			*mtlD*		Mannitol-1-phosphate 5-dehydrogenase
				*mtlF*		PTS system mannitol-specific EIIA component
*trePAR*		(CcpA)	Utilization of secondary carbon sources	*treP*		PTS system trehalose-specific EIIBC component
				*treA*		Trehalose-6-phosphate hydrolase
				*treR*		Trehalose operon transcriptional repressor
*cggR-gapA-pgk-tpiA-pgm-emo*		(CcpA), CggR	Utilization of secondary carbon sources	*cggR*		Central glycolytic genes regulator
				*gapA*		Glyceraldehyde-3-phosphate dehydrogenase 1
*xylAB*		CcpA, XylR	Utilization of secondary carbon sources	*xylA*		Xylose isomerase
				*xylB*		Xylulose kinase
*ackA*			Utilization of secondary carbon sources	*ackA*		Acetate kinase
*glpFK*		CcpA	Utilization of secondary carbon sources	*glpK*		Glycerol kinase
				*glpF*		Glycerol uptake facilitator protein
*glpD*			Utilization of secondary carbon sources	*glpD*		Aerobic glycerol-3-phosphate dehydrogenase
*trpP*			Utilization of secondary carbon sources	*trpP*		Probable tryptophan transport protein
*infC-rplT (ribosomal protein gene cluster)*			Ribosome synthesis	*rplT*	Y	50S ribosomal protein, L20
*rpsT (ribosomal protein gene cluster)*			Ribosome synthesis	*rpsT*		30S ribosomal protein, S20
*rpmGA (ribosomal protein gene cluster)*			Ribosome synthesis	*rpmGA*		50S ribosomal protein, L33
*rpsJ-rplQ (ribosomal protein gene clusters)*			Ribosome synthesis	*rplV*		50S ribosomal protein, L22
				*rpsC*	Y	30S ribosomal protein, S3
				*rplP*	Y	50S ribosomal protein, L16
				*rplX*	Y	50S ribosomal protein, L24
				*rplE*	Y	50S ribosomal protein, L5
				*rpsH*	Y	RNA polymerase sigma-H factor
				*rplF*	Y	50S ribosomal protein, L6
				*rplR*	Y	50S ribosomal protein, L18
				*rpsE*	Y	30S ribosomal protein, S5
				*rpmD*	Y	50S ribosomal protein, L30
				*secY*	Y	Protein translocase subunit, SecY
				*adk*	Y	Adenylate kinase
				*mapA*	Y	Methionine aminopeptidase 1
				*rpsK*	Y	30S ribosomal protein, S11
				*rplQ*	Y	50S ribosomal protein, L17
*rplJ-rplL (ribosomal protein gene cluster)*			Ribosome synthesis	*rplL*	Y	50S ribosomal protein, L7/L12
*cwlO*		YycF	Cell wall metabolism	*cwlO*		Peptidoglycan DL-endopeptidase, CwlO
*deoC*			Purine/ pyrimidine metabolism	*deoC*		Deoxyribose-phosphate aldolase
*odhAB*			Energy production	*odhA*		2-Oxoglutarate dehydrogenase E1 component
				*odhB*		Dihydrolipoyllysine-residue succinyltransferase component of 2-oxoglutarate dehydrogenase complex
*qoxD*			Energy production	*qoxD*		Quinol oxidase subunit 4
*atpB*			Energy production	*atpB*		ATP synthase subunit a
*cspB*			Stress response	*cspB*		Major cold-shock protein.
*sigX*			Stress response	*sigX*		RNA polymerase ECF-type sigma factor
*ypzK*			Undefined	*ypzK*		Riboflavin biosynthesis, reductase, *ribT*, *ribbed*

^a^: Information regarding the TUs was acquired from DBTBS and Bsubcyc. [35, 36]

^b^: The down-regulated genes identified by our transcriptomic analysis are indicated with "Y."

^c^: TFs whose binding sites have been identified near the transcriptional start site (TSS) of each gene. Parentheses indicate TFs whose involvement have been suggested by genetic analysis (i.e., gene knockout), but whose binding sites not yet been identified.

^d^: An essential gene is indicated with "Y."

^e^: The information on gene function was mainly acquired from the Panther database (http://www.pantherdb.org/), while the functions of *mtlF*, *cspB*, *ypzK* and *sigX* were obtained from the Bsubcyc (*mtlF*) [36] and BSORF (others) databases (http://bacillus.genome.ad.jp/).

Among the genes that showed highly reduced RNAP binding, nine were included in four TUs identified in our transcriptome analysis as being down-regulated in *rpoA* ^*del*^-expressing cells (the *srf* operon, *mtlAFD*, *mtnKA*, and *rapA-phrA*) (Fig 4; indicated by pink letters, Table 1, Table 3 and S3 Table). Interestingly, the RNAP binding in *rpoA* ^*del*^-expressing cells was also found to be highly reduced for a number of additional genes related to the utilization of secondary carbon sources, including *treP*, *treA*, *treR* (TU: *trePAR*), *cggR*, *gapA* (*cggR-gapA-pgk-tpiA-pgm-eno*), *xylA*, *B* (*xylAB*), *ackA* (*ackA*), *glpK*, *glpF* (*glpFK*), *glpD* (*glpD*) and *trpP* (*trpP*) (Fig 4, Table 3 and S3 Table). Although these secondary carbon metabolism-related genes were not identified in our transcriptome analysis, our RNAP-binding results suggest that their expression levels may be regulated by α-CTD (the reason for the small overlapping of the transcriptome and ChAP-chip results is discussed in the next sub-section).

Greatly reduced RNAP binding was also observed for 16 genes encoding ribosomal proteins (13 essential and three non-essential) and three encoding essential proteins (*secY*, *adk*, *mapA*) from the five clusters of ribosomal protein genes (Fig 4, Tables 1 and 3 and S3 Table; see also S6 Fig (1, 5 through 7) for ribosomal protein gene clusters *infC-rplT*, *rpsT*, *rpmGA*, *rpsJ-rplQ* and *rplJ-rplL*). Although we have only limited information about these TUs and their promoters, and were thus unable to predict whether UP elements or activators contribute to activating these genes in *B*. *subtilis*, our results suggest that certain mechanisms may govern the α-CTD-dependent regulation of transcription at these ribosomal protein gene clusters. The α-CTD-dependent activation of essential ribosomal protein genes might explain why α-CTD is essential for cell viability in *B*. *subtilis*.

### Transcriptome and ChAP-chip analyses are complementary in detecting the impact of the α-CTD deficiency in *B*. *subtilis* cells

The signal intensities determined by transcriptome analysis represent the RNAs accumulated in the cells, while the RNAP binding intensities determined by ChAP-chip analysis indicate the amount of transcribing RNAPs present on a given gene. This might explain the differences in our detection of down-regulation in *rpoA* ^*del*^-expressing cells using these two platforms. To examine this possibility, we evaluated the distribution of these genes in a scatter plot generated from our transcriptome analysis (S7 Fig). We found that the genes with highly reduced RNAP binding tended to be represented by fewer transcripts in *rpoA* ^*del*^-expressing cells versus the *rpoA* ^*int*^-expressing cells (S7 Fig, dark-blue dots), but that genes with higher transcript signal intensities were less often identified as being down-regulated by the criteria used in our transcriptome analysis. This suggests that RNA accumulation may have obscured some of the down-regulated genes in our transcriptome analysis, especially if the genes are highly expressed.

In addition, high background signal in the ChAP-chip data might also be the reason for the small overlapping of down-regulation detected by the transcriptome and ChAP-chip analysis. The background signal reduced the sensitivity of detection of differences in RNAP binding to protein coding regions on *B*. *subtilis* genomic DNA in *rpoA* ^*del*^- and *rpoA* ^*int*^- expressing cells, especially if the genes were expressed at low levels.

In fact, the signal intensities of the genes with highly reduced RNAP binding identified in ChAP-chip (S7A Fig, dark-blue dots in a scatter plot generated from our transcriptome analysis) tended to be higher than those of the down-regulated genes identified in transcriptome (S7A Fig, sky-blue dots). In addition, the signal intensities of genes identified as down-regulated and with highly reduced RNAP binding were intermediate (S7A Fig, pink dots). This clearly supports our hypothesis that ChAP-chip analysis could mainly detect the difference in RNAP binding to relatively highly expressed genes in the *rpoA* ^*del*^- and *rpoA* ^*int*^-expressing cells. In contrast, transcriptome analysis appears to be more suitable for detection of differential expression in relatively poorly expressed genes in *rpoA* ^*del*^- and *rpoA* ^*int*^- expressing cells.

In addition, the intensities of RNAP binding to the up- and down-regulated genes tended to be increased and decreased, respectively, in *rpoA* ^*del*^-expressing cells compared to *rpoA* ^*int*^-expressing cells (S4A Fig, red and blue dots). This tendency was weak but statistically significant (S4B Fig).

Taken together, we concluded that both transcriptome and ChAP-chip results reflected the impact of α-CTD-deficiency on the global transcriptional regulation of *B*. *subtilis*.

### Transcriptional regulation of the genes down-regulated and highly reduced RNAP binding under α-CTD deficiency

We next investigated the transcriptional-regulator binding sites related to the down-regulated genes and the genes that had highly reduced RNAP binding in *rpoA* ^*del*^-expressing cells, using the information on the TUs and their transcriptional regulators deposited in the DBTBS and Bsubcyc databases [35,36]. In *E*. *coli*, the activators are classified as Class I or Class II based on the location of their binding sites and the mode through which they interact with RNAP to activate gene expression. Class I activators bind upstream of the -35 elements of promoters to interact with α-CTD, while Class II activators interact with the σ subunit via a binding sites that overlap with the -35 element of promoters [1]. From the databases, we found nine TUs, including the genes down-regulated and/or highly reduced RNAP binding in *rpoA* ^*del*^-expressing cells, whose activator binding sites are located upstream of the -35 elements of promoters (S1 and S3 Tables). The activators, ComA (regulating three TUs; the *srf* operon, *rapA-phrA*, *rapC-phrC*), RemA (two TUs; *eps* and *yqxM-sipW-tasA*), ManR (two TUs; *manPA-yjdF* and *manR*), YufM (one TU; *maeN*) and YycF(WalR) (one TU; *cwlO*) might have the potential to directly regulate the expression levels of the relevant TUs as Class I activators via their interaction with α-CTD (the down-regulation of *cwlO* was identified by ChAP-chip analysis, others were identified by transcriptome). Meanwhile, the transcriptional regulators, CcpA (regulating one TU; *rbs* operon), SinR (one TU; *yqxM-sipW-tasA*), AbrB (one TU; *spo0E*), CtsR (one TU; *clpE*), LmrA (one TU; *lmrAB*), YycF(WalR) (two TUs; *wapA-yxxG* and *yoeB*), CggR (one TU; *cggR-gapA-pgk-tpiA-pgm-eno*) and XylR (one TU; *xylAB*) might bind to promoter regions and/or transcriptional start sites of the nine TUs (two TUs, the down-regulation of *cggR-eno* and *xylAB* were identified by ChAP-chip analysis, others were identified by transcriptome) (S1 and S3 Tables). Hence, these transcriptional regulators might have the potential to directly repress the expression levels of these TUs. The α-CTD mediated activation might compete with the direct action of the repressors on the promoter region, and therefore, the TUs might be further repressed in the absence of the α-CTD.

### UP elements are located upstream of the -35 promoter elements of genes that show down-regulation and highly reduced RNAP binding in *rpoA* ^*del*^-expressing cells

As the residues responsible for the interaction with the UP elements found in the α-CTD are known to be highly conserved in bacterial RNAPs [4], we next evaluated the contribution of the UP element to regulating the genes that showed down-regulation of transcription and/or highly reduced RNAP binding in *rpoA* ^*del*^-expressing cells.

We first extracted the -41 to -57 sequences of their known promoters [35,36] (35 promoters for 33 TUs, covering 55 genes in total that showed down-regulation of transcription and/or highly reduced RNAP binding in *rpoA* ^*del*^-expressing cells) and compared them with the UP element consensus sequence proposed for *E*. *coli* [4,13,14]. Twelve of the promoters were found to contain UP-element-like sequences in which > 60% of the nucleotides coincided with the consensus sequence [4,13,50] (S8 Fig).

We then further evaluated the sequences upstream of the -35 elements of promoters with respect to the transcriptional start sites (TSSs) of *B*. *subtilis*, which were experimentally determined by new-generation sequencing [51]. We extracted the -38 to -57 sequence of each promoter (corresponding to the TSS) and searched for conserved nucleotides using the Weblogo program [52]. A conserved AT rich sequence was found in two separate regions upstream of the genes that showed down-regulation of transcription and/or highly reduced RNAP binding in *rpoA* ^*del*^-expressing cells (S9 Fig); centred at the -43 and -53 positions, these sequences corresponded to the proximal and distal subsets of the UP elements proposed in *E*. *coli* promoters [4,13]. Little conservation of AT-rich sequences was detected upstream of the -35 promoter elements of the other tested genes. These results suggest that although it is rare for the entire UP element sequence to be conserved upstream of the -35 element of the promoter of a putative α-CTD-dependent gene, such elements are partially conserved, potentially contributing to the activation of gene expression in *B*. *subtilis*. This is consistent with the previous observation that half of an UP element is partially able to activate transcription in *E*. *coli* [4].

### ChAP-chip analysis reveals two types of α-CTD dependent transcriptional activation in *B*. *subtilis*

**Fig 5:**
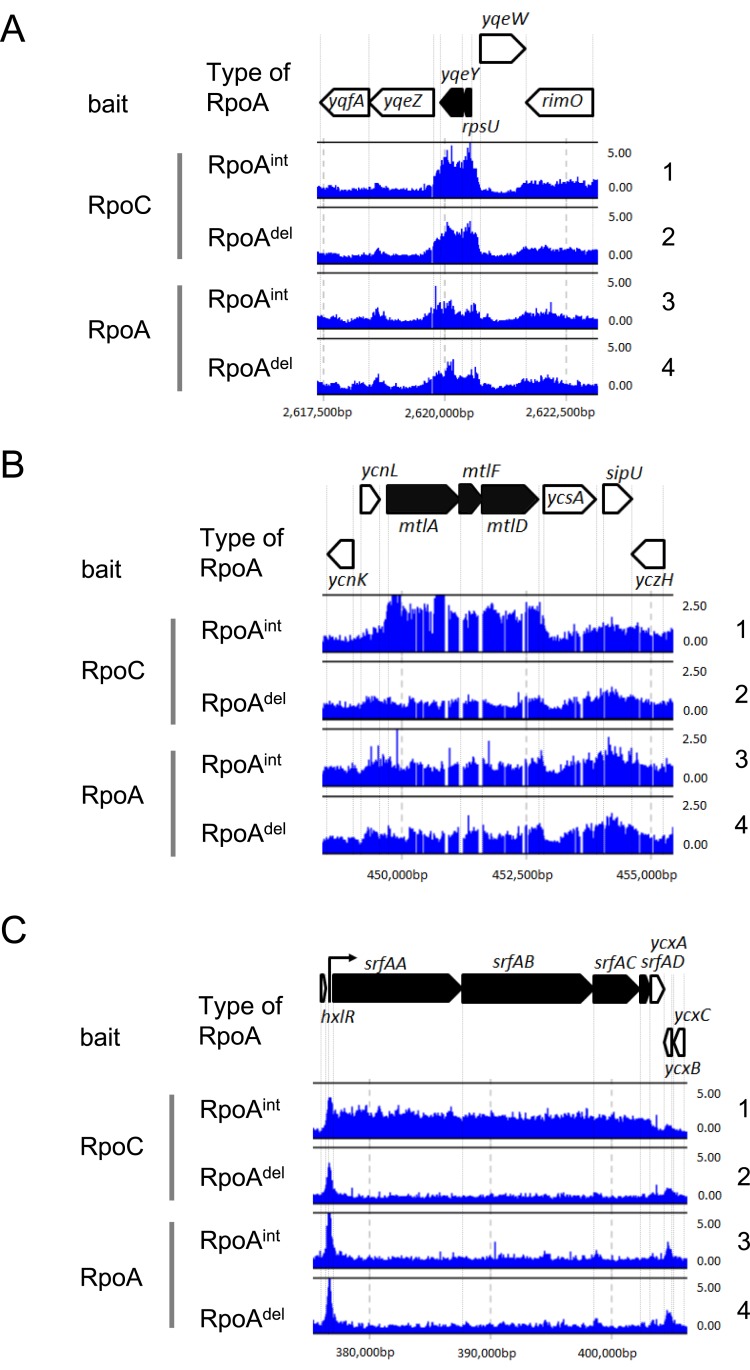
RNAP binding effects following the replacement of RpoA^int^ with RpoA^del^ in the RNAP complex. (A) Typical RNAP binding profiles obtained from *rpoA* ^*int*^- and *rpoA* ^*del*^-expressing cells; *rpsU* and *yqeY* are indicated, with the RNAP binding signal of each probe mapped to the corresponding position in the *B*. *subtilis* chromosome. The binding intensity (shown by vertical bars) was determined as the relative ratio of the signal intensities obtained for the hybridization of labeled DNA fragments prepared from the affinity purification with RpoC or RpoA (ChAP DNA) versus whole cell extract (control DNA) fractions in each experiment. The RNAP binding intensities determined by affinity purification with RpoC as bait are shown in lanes 1 and 2. The RNAP binding intensities determined by affinity purification with RpoA as bait are shown in lanes 3 and 4. The RNAP binding intensities in the *rpoA* ^*int*^-expressing cells are shown in lanes 1 (SMS08) and 3 (SMS18), and those in *rpoA* ^*del*^-expressing cells are indicated in lanes 2 (SMS09) and 4 (SMS19). The arrangement of genes in the presented chromosomal region is indicated by thick arrows at the top of the figure. The RNAP binding profiles obtained from one (Exp. 1) of duplicate experiments are shown as representative. (B) Binding to the *mtl* operon is shown as a typical example of the reduced RNAP binding observed in *rpoA* ^*del*^-expressing cells. (C) Binding to the *srf* operon is shown as an example of the unique RNAP binding profile observed in *rpoA* ^*del*^-expressing cells, in which reductions were seen in the protein coding regions but not in the promoter proximal regions.

To examine the effects of α-CTD deficiency on RNAP binding, we assessed the RNAP binding profiles of the genes that showed highly reduced RNAP binding in *rpoA* ^*del*^ cells upon visual inspection of the RNAP (especially RpoC-His) distributions. The typical distributions of RNAP (revealed by using RpoC-His or RpoA-His) on the *B*. *subtilis* chromosome are shown in Fig 5A, and the binding profiles of RNAP throughout the chromosome of *B*. *subtilis* (as assessed in duplicate experiments) are presented in S10 Fig.

The RNAP (RpoC-His) binding signals were generally similar in the *rpoA* ^*del*^- and *rpoA* ^*int*^-expressing cells (Fig 5A, lanes 1 and 2), but they were reduced across the promoters and protein-coding regions for most of the TUs identified as having highly reduced RNAP binding in *rpoA* ^*del*^-expressing cells (Fig 5B, lanes 1 and 2, and S6 Fig (1 through 7). Thus, α-CTD deficiency appears to inhibit the recruitment of RNAP to many promoters in *B*. *subtilis*.

Interestingly, we observed unique RNAP binding profiles for *cggR-gapA*, *odhAB*, *qox* and the *srf* operon in *rpoA* ^*del*^-expressing cells: the RNAP (RpoC-His) binding signals were reduced in the coding regions, but peaked across the promoters or promoter-proximal regions of these genes (Fig 5C, lanes 1 and 2; S6 Fig (8 and 9). These results suggest that RNAP could be recruited to these gene promoters in the absence of α-CTD, but that α-CTD is needed to form the productive complex or overcome pausing or termination signals located in the promoter-proximal regions. While α-CTD could be involved in anti-termination activity or another regulatory activity governing transcriptional elongation, we believe that it is more likely to be involved at the transcriptional initiation step. A previous study showed that the *E*. *coli* transcriptional activator, CRP, interacts with α-CTD, and that this enhanced formation of the productive open complex at the *malT*, *lac* and *gal* promoters; in the absence of CRP, in contrast, RNAP formed non-productive (*malT*) or less active (*lac* and *gal*) RNAP-promoter complexes [53]. The RNAP binding profiles determined herein for the *cggR-gapA*, *odhAB*, *qox* and *srf* operons suggest that α-CTD may also enhance formation of the productive complex in *B*. *subtilis* by interacting with transcriptional activators.

## Conclusions

In this study, we used transcriptome and ChAP-chip analyses to investigate the role of α-CTD in *B*. *subtilis* cells, and identified 53 genes whose mRNA levels were down-regulated and 50 genes whose RNAP binding was highly reduced in α-CTD-deficient cells compared to controls (Fig 4, Table 1). Interestingly, relatively few genes overlapped between the two data sets (nine genes, Table 1). We speculate that whereas the ChAP-chip analysis could detect the effect of α-CTD deficiency on highly expressed genes, accumulated mRNA may have reduced the ability of our transcriptome analysis to detect reductions in mRNA expression and the higher background of ChAP-chip analysis reduced the sensitivity of detection of the reduction of RNAP binding to low expression genes (S7A Fig). Thus, our combined use of transcriptome analysis plus ChAP-chip analysis enabled us to examine the effects of α-CTD deficiency on transcriptional regulation more comprehensively than transcriptome analysis alone.

Our analysis of the biological functions of the genes that showed down-regulation or highly reduced RNAP binding under α-CTD deficiency indicated that α-CTD may be important to the transition-state response, the utilization of secondary carbon sources, and ribosomal protein synthesis. A time-course study of our transcriptome data revealed that the effect of α-CTD deficiency appeared beginning at 1 hour of cultivation under the inducing conditions (Fig 3B) and increased with time (Fig 3C and 3D). We believe that the effects identified in this study are mainly the direct effects of α-CTD deficiency, although some secondary effects may also have contributed to our findings.

We investigated the activation-related elements upstream of the -35 elements of promoters for the TUs found to undergo down-regulation in α-CTD-deficient cells, and found the transcriptional activator binding sites for nine of the TUs. Furthermore, we found predicted UP elements upstream of the -35 elements of 12 promoters for the genes that showed down-regulation of transcription and/or highly reduced RNAP binding in *rpoA* ^*del*^-expressing cells (S8 Fig). In addition, UP element sequences appear to be at least partially conserved in the regions upstream of some of the TUs that show mRNA down-regulation and/or reduced RNAP binding under α-CTD deficiency (S9 Fig). Together, our results suggest that the interaction of α-CTD with activators and UP elements may enhance the expression of multiple promoters in *B*. *subtilis*, as has been seen in *E*. *coli*. Although future *in vitro* work is warranted to confirm the activity of the UP element/activator for each promoter, our results strongly suggest that, consistent with previous findings in *E*. *coli*, α-CTD contributes to global transcriptional regulation in *B*. *subtilis* cells.

An important finding of this study is that α-CTD appears to contribute to positively regulating genes involved in carbon catabolite repression in *B*. *subtilis* (S11 Fig) [54]. Bacteria have a phosphoenolpyruvate-carbohydrate phosphotransferase system (PTS) composed of multiple proteins (enzyme I [EI], histidine protein [HPr] and enzyme II [EII]) that sense the consumption of carbon sources to control their transport [54]. In *B*. *subtilis*, PTS functions in transcriptional regulation: HPr kinase (HPrK) phosphorylates serine-46 of HPr, and the resulting HPr(Ser-P) interacts with CcpA to repress many genes related to the utilization of secondary carbon sources in the presence of glucose; meanwhile, under glucose depletion, EI phosphorylates histidine-15 in HPr to generate HPr(His-P), thereby reducing the amount of the HPr(Ser-P)-CcpA complex and abrogating CcpA-mediated gene repression. HPr(His-P) also phosphorylates activators that contain the PTS-regulatory domain (PRD) and activates gene expression related to the transport of secondary carbon sources (S11 Fig) [54]. Our results indicate that *rpoA* ^*del*^-expressing cells show decreased mRNA levels and/or RNAP bindings of the *rbs* operon, the *gmu* operon, *trePAR*, *glpFK*, *cggR-gapA* and *xylAB* (whose expression levels are repressed by CcpA [44,45,55–58]), as well as *manPA*, *manR* and *mtlAFD* (whose expression levels are activated by the PRD-containing regulators, ManR and MtlR [48,49]) (S11 Fig). This clearly indicates that α-CTD deficiency impairs the transcriptional response underlying cellular adaptation to glucose depletion. In *B*. *subtilis*, α-CTD may compete with CcpA-mediated repression by enhancing transcription via UP elements and/or other activators. In *E*. *coli*, the formation of the CRP-cAMP complex in the absence of glucose is coupled with PTS, and the CRP-cAMP complex activates the expression of genes involved in the utilization of secondary carbon sources via an interaction with α-CTD [54,59]. Thus, although α-CTD contributes to gene expression related to the utilization of secondary carbon sources in *B*. *subtilis* and *E*. *coli*, the mechanism through which α-CTD activates the utilization of secondary carbon sources appears to differ between *B*. *subtilis* and *E*. *coli*.

Another important finding of the present study is the role of α-CTD in regulating the expression of ribosomal protein genes in *B*. *subtilis*. In *E*. *coli*, α-CTD directly regulates rRNA expression through interactions with the UP element of *rrn* P1 and the activator, Fis [4]. In addition, the transcription of ribosomal protein genes and rRNA is inhibited by DksA and ppGpp in *E*. *coli* [60]. In *B*. *subtilis*, however, the UP element contributes only weakly to rRNA expression and there is no DksA homolog [23]. Our present results suggest that α-CTD positively regulates the expression of many ribosomal protein genes in *B*. *subtilis* (16 genes, Table 1). Therefore, it was suggested that although α-CTD contributes to ribosome synthesis in both *E*. *coli* and *B*. *subtilis*, its specific role differs between the two organisms. Interestingly, α-CTD was shown to participate in the transcriptional regulation of the ribosomal protein gene, *rpsE*, in *S*. *aureus* [21]. Thus, the α-CTD dependent transcriptional regulation of ribosomal protein genes might be conserved in Gram-positive bacteria.

Our ChAP-chip analysis revealed that α-CTD deficiency negatively affected the expression of ribosomal protein genes. Our transcriptional analysis failed to show this effect, perhaps due to the presence of mRNAs that had accumulated prior to the induction of α-CTD deficiency. This might explain why the *rpoA* ^*del*^-expressing cells could grow for at least 6 hours in LB^xyl^, but could not form colonies on LB plates containing 1% xylose.

Our ChAP-chip results also suggested that α-CTD contributes to transcriptional expression in *B*. *subtilis* cells by activating the recruitment of RNAP to promoters and enhancing the formation of the productive (elongation) complex on the promoters of genes found to show down-regulation or reduced RNAP binding in *rpoA* ^*del*^-expressing cells.

In sum, our results collectively suggest that the biological importance of α-CTD is conserved in *B*. *subtilis* and *E*. *coli*, but that its specific roles have diversified between these two bacteria.

## Materials and Methods

### Construction of bacterial strains

The bacterial strains and primers used in this study are listed in S4 and S5 Tables, respectively. The *B*. *subtilis* strains used in this study were derived from *B*. *subtilis* strain 168. Strain SMS01 (168 *rpoA*::*cat*-P*spac*-*rpoA* ^*int*^-*rplQ*) was constructed by transformation of strain 168 with the DNA fragment encoding: the IPTG-inducible promoter (P*spac*), the *lacI* and chloramphenicol resistance (*cat*) genes, flanked by sequences homologous to the 3’ region of *rpsK* with its downstream sequence and the 5’ region of the *rpoA* gene with its upstream sequence. The latter enabled the double-crossover recombination of the DNA fragment with the *B*. *subtilis* chromosome, thereby introducing the P*spac* promoter into the intergenic region between *rpsK* and *rpoA*. The stepwise PCR-based procedures used to construct the recombinant strains are schematically shown in S12 Fig. We initially amplified various short DNA fragments as follows: the 3’ region of the *rpsK* gene with its downstream region was amplified from genomic DNA of strain 168 using primers SMP01 and SMP02 (fragment A); *cat*, *lacI* and the P*spac* promoter were amplified from the genomic DNA of 168*spacZ* (S. Ishikawa, unpublished) using primers SMP03 and SMP04 (fragment B); and the 5’ region of the *rpoA* gene with its upstream region was amplified from strain 168 genomic DNA with primers SMP05 and SMP06 (fragment C). Fragment A–C were joined together to generate the DNA fragment that was used to transform strain 168 by recombinant PCR. After transformation, chloramphenicol-resistant colonies were selected to obtain SMS01. SMS02 (168 *amyE*::P*xyl*-*rpoA* ^*int*^-*rplQ-km*) was similarly constructed by the transformation of strain 168 with a DNA fragment harboring the xylose-inducible promoter (P*xyl*), the intact *rpoA* gene, the *rplQ* gene and the kanamycin resistance gene (*km*), flanked by sequences homologous to the 5’ region of the *xylR* gene and the 3’ region of the *amyE* gene. Double crossover recombination thus introduced the *rpoA-rplQ* genes along with the P*xyl* promoter into the *amyE* locus of the *B*. *subtilis* chromosome (S12 Fig). The DNA P*xyl* promoter was amplified from LY111 genomic DNA [61] using primers SMP07 and SMP08 (fragment D); full-length *rpoA* and *rplQ* were amplified from strain 168 using primers SMP09 and SMP12 (fragment E); the kanamycin-resistant fragment was obtained by PCR using plasmid pDG780 [62] and primers SMP13 and SMP14 (fragment F); and the *amyE* 3’ region was amplified from LY111 genomic DNA using primers SMP15 and SMP16 (fragment G). These four DNA fragments were concatenated by recombinant PCR, and the resulting DNA fragment was used to transform LY111 cells (S12 Fig). The DNA fragment used to construct strain SMS03 (168 *amyE*::P*xyl*-*rpoA* ^*del*^-*rplQ-km*) was generated by recombinant PCR using DNA fragments D, F and G, plus additional fragments H and I, which were PCR amplified from strain 168 genomic DNA using primers SMP09 and SMP17, and SMP11 and SMP12, respectively (S12 Fig). The amplified DNA fragments carried a truncated *rpoA* gene consisting of 1–733 bp and 931–945 bp (the 3’ end) of *rpoA*, *rplQ*, and the intact intergenic region between *rpoA* and *rplQ*, all located downstream of the P*xyl* promoter. The generated fragment was introduced into the *amyE* locus of the LY111 chromosome. The DNA fragment used to construct SMS04 (168 *amyE*::P*xyl*-*rplQ-km*) consisted of fragments D, F and G, and additional fragment J, which carried *rplQ* and the intact intergenic region between *rpoA* and *rplQ* (including the termination codon of the *rpoA* gene). SMS04 was obtained by the transformation of LY111 with the amplified DNA fragment. Kanamycin-resistant colonies were selected to obtain strains SMS02, -03 and -04. These strains were then transformed with genomic DNA purified from SMS01 to construct SMS05 (168 *rpoA*::*cat*-P*spac*-*rpoA* ^*int*^-*rplQ*, *amyE*::P*xyl*-*rpoA* ^*int*^-*rplQ-km*), SMS06 (168 *rpoA*::*cat*-P*spac*-*rpoA* ^*int*^-*rplQ*, *amyE*::P*xyl*-*rpoA* ^*del*^-*rplQ-km*) and SMS07 (168 *rpoA*::*cat*-P*spac*-*rpoA* ^*int*^-*rplQ*, *amyE*::P*xyl*-*rplQ-km*), respectively. SMS05, SMS06 and SMS07 were transformed with genomic DNA purified from 168 rpoC-His, and erythromycin-resistant colonies were obtained and designated SMS08 (168 *rpoA*::*cat*-P*spac*-*rpoA* ^*int*^-*rplQ*, *amyE*::P*xyl*-*rpoA* ^*int*^-*rplQ-km*, *rpoC*::pMUTinHis-*Em* Δ*rpoC*), SMS09 (168 *rpoA*::*cat*-P*spac*-*rpoA* ^*int*^-*rplQ*, *amyE*::P*xyl*-*rpoA* ^*del*^-*rplQ-km*, *rpoC*::pMUTinHis-*Em* Δ*rpoC*), respectively. To construct SMS16 (168 *amyE*::P*xyl*-*rpoA* ^*int*^ *His*-*rplQ-tet*) and SMS17 (168 *amyE*::P*xyl*-*rpoA* ^*del*^ *His*-*rplQ-tet*), we initially constructed SMS14 (168 *amyE*::P*xyl*-*rpoA* ^*int*^-*km*) and SMS15 (168 *amyE*::P*xyl*-*rpoA* ^*del*^ *-km*). For the construction of SMS14, we used fragments D, G, additional fragment K, which was amplified from strain 168 genomic DNA using primers SMP09 and SMP10, and fragment L, which was amplified from plasmid pDG780 using primers SMP27 and SMP14. These fragments were concatenated by recombinant PCR and transformed to LY111 cells to generate SMS14. For the construction of SMS15, we used fragments D, G, L, and additional fragment M, which was amplified from strain 168 genomic DNA using primers SMP09 and SMP17 (S12 Fig). For the construction of SMS16, we used fragment N, which included the 3’ region of the *rpoA* gene and was amplified from strain 168 genomic DNA using primers SMP28 and SMP29, fragment O, which encoded a 12 x Histidine tag and was amplified from strain 168 genomic DNA using primers SMP30 and SMP31, and fragment P, which carried the tetracycline resistance gene (*tet*) and the *amyE* 3’ region and was amplified from LY111 genomic DNA using primers SMP32 and SMP16. Strain SMS14 was transformed with the generated fragment and tetracycline was used to obtain SMS16. Similarly, strain SMS17 was constructed by the transformation of strain SMS15 with the DNA fragment consisting of fragment Q, which included the truncated 3’ region of the *rpoA* gene and was amplified from strain 168 genomic DNA using primers SMP33 and SMP34, fragment O and fragment P (S12 Fig). Transformants were selected based on tetracycline resistance. Finally, SMS18 (168 *rpoA*::*cat*-P*spac*-*rpoA* ^*int*^-*rplQ*, *amyE*::P*xyl*-*rpoA* ^*int*^ *His*-*rplQ-tet*) and SMS19 (168 *rpoA*::*cat*-P*spac*-*rpoA* ^*int*^-*rplQ*, *amyE*::P*xyl*-*rpoA* ^*del*^ *His*-*rplQ-tet*) were generated by transforming strains SMS16 and SMS17 with the chromosomal DNA of strain SMS01.

### Growth conditions

All experiments were performed under the same culture conditions. *B*. *subtilis* cells were grown overnight at 37°C on LB plates containing 1 mM IPTG (to induce the expression of RpoA^int^) and antibiotics, if needed. For pre-culture, *B*. *subtilis* cells were suspended in 50 mL of fresh LB^IPTG^ [Luria-Bertani (LB) medium containing 1 mM IPTG] at OD_600_ ≈ 0.01 and grown to mid-log phase (OD600 ≈ 0.4) at 37°C under aerobic conditions. The cells were harvested by centrifugation (6000 rpm for 10 min) and washed twice with fresh LB medium. The cells were then resuspended in 400 mL of fresh LB^xyl^ (LB containing 1% xylose) at OD_600_ ≈ 0.03, and experimental cultivations were performed at 37°C under aerobic conditions.

### Pull-down purification of RNAP complexes

*B*. *subtilis* cells expressing Histidine-tagged RpoC (SMS08 cells expressing *rpoA* ^*int*^ and SMS09 expressing *rpoA* ^*del*^) were grown in LB^xyl^ at 37°C under aerobic conditions, as described above. The cells were cultivated in LB^xyl^, harvested by centrifugation hourly from 0 to 6 hours, and resuspended in 1.5 mL of binding buffer (0.1 M Tris-HCl, pH 7.5, 20% glycerol, 1 mM β-mercaptoethanol, 50 mM imidazole, 0.5 M NaCl, 1 mM PMSF), and sonicated for a total of 10 min (4-second pulses with 10-second intervals). The lysates were then centrifuged (8000 rpm for 10 min at 4°C), and the supernatants (taken as whole-cell extracts) were mixed with 50 μL of nickel-affinity beads (His-Tag Isolation & Pulldown, Invitrogen). The mixtures were incubated for 30 min at 4°C, and the beads were removed by a brief centrifugation and the use of a magnetic rack. After the beads were subjected to five washes with binding buffer, the immobilized RNAP complexes were eluted with 50 μL of elution buffer (0.1 M Tris-HCl, pH 7.5, 20% glycerol, 1 mM β-mercaptoethanol, 0.5 M imidazole, 0.1 M NaCl). The recovered RNAP complexes were resolved on a 5–20% SDS-PAGE gradient gel, and the results were visualized with the Flamingo (Bio-Rad) fluorescent stain. Gel densitometry was performed using the ImageJ software (NIH). The bands corresponding to RpoC-His, RpoA^int^ and RpoA^del^ were confirmed by western blotting using anti-His-tag and anti-RpoA antibodies.

### Western blotting

Western blotting was performed as described previously [33]. The rabbit poly-clonal anti-RpoA antibody was generated against a mixture of chemically synthesized peptides corresponding to residues 143 to 159 (QRGRGYTPADANKRDDQ), 233 to 250 (HAEIMVEKEEDQKEKVLE), and 297 to 314 (EEVKAKLEELGLGLRKDD) of RpoA. The rabbit immunizations, serum preparation and antibody purification were performed by SIGMA Genosys (Japan). The poly-clonal anti-His-tag antibody and mono-clonal anti-RpoB antibody were purchased from MBL (Japan) and Neoclone (USA), respectively.

### Transcriptome analysis

Total RNA was isolated from *rpoA* ^*int*^- and *rpoA* ^*del*^-expressing cells harvested hourly from 0 to 3 hours after the change of medium (from LB^IPTG^ to LB^xyl^), cDNAs were prepared from each of these eight RNA samples (5 μg), and fragmented cDNAs were subjected to terminal labeling, as previously described [33]. Hybridization and Genechip scanning were performed using a Genechip *B*. *subtilis* genome array and the GCOS software (Affymetrix); an appropriate hybridization condition (at 45°C for 16 hours) and wash and staining protocol for the array, ProkGE-WS2, according to the manufacture’s instruction (Affymetrix). All experiments were performed in duplicate. The signal intensities obtained from all probes were normalized using the GCOS software, with the target signal intensity set to 500 (Affymetrix). To exclude genes with low expression levels, we selected the probes corresponding to the genes with adequate signal intensities (sum of the signal intensities in duplicate experiments, each involving two hybridizations (from dupricate experiments) with SMS08 cells and two with SMS09 cells > 400). The genes that were up- and down-regulated in SMS09 cells versus SMS08 cells were those having relative signal intensity ratios < 0.25 or > 4 and *q* < 0.2 by Welch’s t-test. The raw transcriptome data have been deposited in Arrayexpress under accession, E-MTAB-3383.

### ChAP-chip analysis

ChAP-chip analysis was performed as previously described [33]. Briefly, after the medium was changed from LB^IPTG^ to 400 ml LB^xyl^, cells were grown with aeration at 37°C for 3 hours, and then incubated with formaldehyde (final concentration, 1%) for 30 min at 37°C to crosslink the RNAP complex to the chromosomal DNA. The cells were then pelleted by centrifugation and resuspended in 3 ml of UT buffer (100 mM HEPES, 50 mM imidazole, 8 M urea, 0.5 M NaCl, 1% Triton X-100, and 10 mM β-mercaptoethanol, pH 7.4). The affinity purification of the RNAP-DNA complexes, purification of DNA from these RNAP-DNA complexes and original Whole-cell extract, and amplification of purified DNA for labeling were all performed as previously described [33]. A custom Affymetrix tiling array for *B*. *subtilis* [33] was used, and signal intensities were acquired using the GCOS software in CEL format. The In Silico Molecular Cloning Program, Array Edition (In Silico Biology, Japan) was used to determine the binding intensities and visualize the binding profiles of RNAP on the *B*. *subtilis* chromosome. The signal intensities of DNA affinity-purified with RNAP (ChAP DNA) and purified from whole-cell extracts (control DNA) were adjusted to confer a signal average of 500 for normalization, and the RNAP (RpoC and RpoA) binding intensities of all probes were determined based on their enrichment of DNA fragments by the affinity purification of RNAP. This was calculated by dividing the signal intensities of ChAP DNA by those of control DNA [33]. All experiments were performed in duplicate. The raw ChAP-chip data have been deposited in Arrayexpress under accession numbers, E-MTAB-3384 and -3385.

## Supporting Information

S1 Fig Alignment of amino acid sequences for the *B*. *subtilis* (Bs) and *E*. *coli* (Ec) RpoA proteins. Alignment was performed using ClustalW. The boundary positions between the N-terminal domain (α-NTD) and the linker, and between the linker and the C-terminal domain (α-CTD) (as determined for *E*. *coli* RpoA [4]) are shown. Red letters represent the region deleted in the C-terminally truncated RpoA^del^.
(PDF)

S2 Fig Growth of *B*. *subtilis* cells streaked on LB plates without supplementation (A), or in the presence of 1 mM IPTG (B) or 1% xylose (C). *RpoA* ^*del*^-expressing cells (SMS05; P*spac*-*rpoA* ^int^-*rplQ*, P*xyl*-*rpoA* ^int^ *-rplQ*) were streaked on the left of each plate, and *rpoA* ^*int*^-expressing cells (SMS06; P*spac*-*rpoA* ^int^-*rplQ*, P*xyl*-*rpoA* ^del^-*rplQ*) were streaked on the right.
(PDF)

S3 Fig Western blotting analysis of RNAP complex (RpoB) in crude extracts of *rpoA* ^*int*^-expressing cells (SMS08) and *rpoA* ^*del*^-expressing cells (SMS09). The crude lysates were prepared by the same procedure for the pull down assay. The amount of applied samples to SDS-PAGE were adjusted by the OD_600_ unit for cell culture. The duration of cultivation in LB^xyl^ is shown at the bottom of each panel.
(PDF)

S4 Fig ChAP-chip analysis of RNAP (RpoC-His) in *rpoA* ^*int*^-expressing cells (SMS08) and *rpoA* ^*del*^-expressing cells (SMS09). (A) Scatter plot of the RNAP binding intensity of each gene in *rpoA* ^*del*^-expressing cells (vertical axis) versus *rpoA* ^*int*^-expressing cells (horizontal axis). Genes identified in our transcriptome analysis as being up- and down-regulated in *rpoA* ^*del*^-expressing cells at 3 hours after the beginning of the RpoA^del^ induction are shown as red and blue dots, respectively. The experiments were performed duplicate, and are shown as Exp. 1 and Exp. 2. The correlation coefficients for the RNAP binding intensities in *rpoA* ^*int*^-expressing and *rpoA* ^*del*^-expressing cells are indicated as (*r*) in each panel. (B) Distribution of the relative ratios of RNAP binding intensity (RpoC-His binding intensity) for each gene in *rpoA* ^*int*^- and *rpoA* ^*del*^-expressing cells (relative ratio = [RNAP binding intensity in *rpoA* ^*int*^-expressing cells] / [RNAP binding intensity in *rpoA* ^*int*^-expressing cells]). Three gene sets are shown, as assessed in *rpoA* ^*del*^-expressing cells compared to *rpoA* ^*int*^-expressing cells: all genes subjected to our ChAP-chip analysis (left); genes that were up-regulated in *rpoA* ^*del*^-expressing cells per our transcriptome analysis at 3 hours after the beginning of the RpoA^del^ induction (middle); and genes that were down-regulated *rpoA* ^*del*^-expressing cells per our transcriptome analysis at 3 hours after the beginning of the RpoA^del^ induction (right). Box plots represent the median (horizontal black lines), the upper and lower quartile values (boxes), and the most extreme data points within 1.5-fold interquartile ranges (whiskers). The asterisks indicate statistically significant differences between the gene sets, as assessed by the Wilcoxon rank-sum test (**: *p*-value < 0.01).
(PDF)

S5 Fig ChAP-chip analysis of RpoA in *rpoA* ^*int*^-expressing cells (SMS18) and *rpoA* ^*del*^-expressing cells (SMS19). Scatter plots of the RpoA binding intensity for each gene in SMS19 cells (vertical axis) versus SMS18 cells (horizontal axis) in duplicate experiments. The correlation coefficients of the RNAP binding intensities in SMS18 and SMS19 cells are indicated as (*r*) in each panel.
(PDF)

S6 Fig RNAP binding profiles of the top 50 genes showing the highest reductions in RNAP binding. The results are shown as described for Fig 5. The thick blue arrow at the top of the figure indicates the genes most highly reduced in RNAP binding. Arrow heads in S6 Fig (8 and 9) indicate “peaks” of RNAP observed at promoter or promoter proximal regions in *rpoA* ^*del*^ expressing cells.
(PDF)

S7 Fig The distribution of the genes most highly reduced in their RNAP binding identified by ChAP-chip analysis in the scatter plot of the transcriptional signal intensity of each gene in *rpoA* ^*del*^- and *rpoA* ^*int*^-expressing cells. (A) The scatter plot is same with Fig 3D. The dark-blue dots indicate genes most highly reduced RNAP binding (identified by ChAP-chip analysis). The sky-blue dots indicate genes whose transcription were down-regulated in *rpoA* ^*del*^- expressing cells (identified by transcriptome analysis). The pink dots represent genes highly reduced RNAP binding and down-regulated in *rpoA* ^*del*^- expressing cells (identified by both ChAP-chip and transcriptome analysis). (B) Enlarged view of the area containing the genes that showed the highest signal intensities in our transcriptome analysis.
(PDF)

S8 Fig Candidate UP elements for known promoters of 12 genes found to be down-regulated and/or highly reduced RNAP binding in *rpoA* ^*del*^-expressing cells. The sequences spanning from -41 to -57 are compared with the UP element consensus sequence. The conserved nucleotides are shown in red letters. The difference between each candidate sequence and the consensus sequence is shown at the right of the figure. The *clpE* and *mtnX* genes have two promoters for each gene; in these cases, the “up” notation indicates the upstream promoter.
(PDF)

S9 Fig The analysis of upstream regions of the -35 elements of promoters for genes that are down-regulated and/or highly reduced RNAP binding in *rpoA* ^*del*^-expressing cells (SMS09) compared with *rpoA* ^*int*^-expressing cells (SMS08). Data on the transcriptional start sites (TSSs) were taken from Irnov et al. (2010) [51]. The DNA sequences upstream of each promoter (-38 to -57 bp) were acquired and numbered with respect to the TSS (bottom of bar graphs). The nucleotide frequency in each position was indicated as bar graphs. The nucleotide conservation at each position analyzed using the Weblogo program (http://weblogo.threeplusone.com/) [52] was indicated under bar graphs. The locations of the proximal and distal portions of the putative UP elements are indicated in figures of logo. We identified 25 TSSs corresponding to the genes identified as showing down-regulation (transcriptomic analysis) and highly decreased RNAP binding (ChAP-chip analysis) in *rpoA* ^*del*^-expressing cells: *cggR*, *cspB*, *cwlO*, *deoC*, *glpD*, *glpF*, *odhA*, *rapA*, *rpmGA*, *rpsT*, *srfAA*, *trpP*, *ypzK*, *comQ*, *pel*, *rapC*, *spo0E*, *wapA*, *xpt*, *yrhP*, *ywsB*, *iseA*, *yisT*, *yuiF* and *yxbC*. (A) The nucleotide frequency and conservation in the DNA sequences upstream of 25 promoters (-38 to -57 bp) for the genes down-regulated and highly decreased RNAP binding in *rpoA* ^*del*^-expressing cells. (B) The nucleotide frequency and conservation in the DNA sequences upstream of the other tested genes.
(PDF)

S10 Fig Genome-wide RNAP binding profiles determined by ChAP-chip analysis. **RpoA** ^**int**^ **-His**, **RpoA** ^**del**^ **-His and RpoC-His were used as bait for the purification of RNAP complexes**. The gene arrangement on the *B*. *subtilis* chromosome is shown by thick arrows at the top of the figure. Colors: sky blue indicates genes that are down-regulated in *rpoA* ^*del*^-expressing cells compared with *rpoA* ^*int*^-expressing cells, as determined by transcriptomic analysis; green indicates the top 50 genes showing the greatest reductions in RNAP binding among *rpoA* ^*del*^-expressing cells versus *rpoA* ^*int*^-expressing cells; and dark blue indicates the genes that showed both transcriptomic down-regulation and high-level reductions in RNAP binding in *rpoA* ^*del*^-expressing cells.
(PDF)

S11 Fig The expression of genes (TUs) down-regulated or highly decreased in RNAP binding in *rpoA* ^*del*^-expressing cells are regulated by the carbon catabolite repression in *B*. *subtilis*. The pathway for phosphorylation in the presence of glucose is indicated by red arrows, while that for phosphorylation in the absence of glucose is indicated by black arrows. The phosphoenolpyruvate-carbohydrate phosphotransferase system (PTS) is shown in the middle of the figure, with EI (enzyme I), HPr (histidine protein) and EII (enzyme II) shown in the blue square. The phosphorylated forms of HPr and PRD (the PTS-regulatory domain) containing activator are indicated with the circled-P notation. Unphosphorylated HPr is phosphorylated by HPr kinase (HPrK) in the presence of glucose, or by EI in the absence of glucose. The genes that showed mRNA down-regulation and major reductions in RNAP binding in *rpoA* ^*del*^-expressing cells and are known to be repressed by CcpA and activated by ManR and MtlR are indicated at the bottom left (the expression of those genes are repressed by CcpA) and the bottom right (the expression of those genes are activated by PRD containing activators, MtlR and ManR) of the figure.
(PDF)

S12 Fig Recombinant PCR procedures used to construct the utilized strains. Schematic representation of the primers used to amplify *B*. *subtilis* chromosomal and plasmid sequences (fragments A to P). Black bars indicate the complementary primer sequences used for recombinant PCR.
(PDF)

S1 Table Genes up- and down-regulated in SMS09 cells (expressing RpoA^del^) compared to SMS08 cells (expressing RpoA^int^) at 3 hours after the change of medium. (XLSX)

S2 Table Genes up- and down-regulated in SMS09 cells (expressing RpoA^del^) compared to SMS08 cells (expressing RpoA^int^) at 2 hours after the change of medium. (XLSX)

S3 Table The top 50 genes showing the highest reductions of RNAP binding in SMS09 cells versus SMS08 cells. (XLSX)

S4 Table Bacterial strains used in this study. (XLSX)

S5 Table Primers used in this study. (XLSX)
