# Supplementary material for: The Role of α-CTD in the Genome-Wide Transcriptional Regulation of the Bacillus subtilis Cells
Source: PLoS One. 2015 Jul 8;10(7):e0131588. doi: 10.1371/journal.pone.0131588 (PMC4495994; doi:10.1371/journal.pone.0131588)
Supplement: S1 Fig — Alignment was performed using ClustalW. The boundary positions between the N-terminal domain (α-NTD) and the linker, and between the linker and the C-terminal domain (α-CTD) (as determined for E. coli RpoA [4]) are shown. Red letters represent the region deleted in the C-terminally truncated RpoAdel. (PDF) [file pone.0131588.s001.pdf]

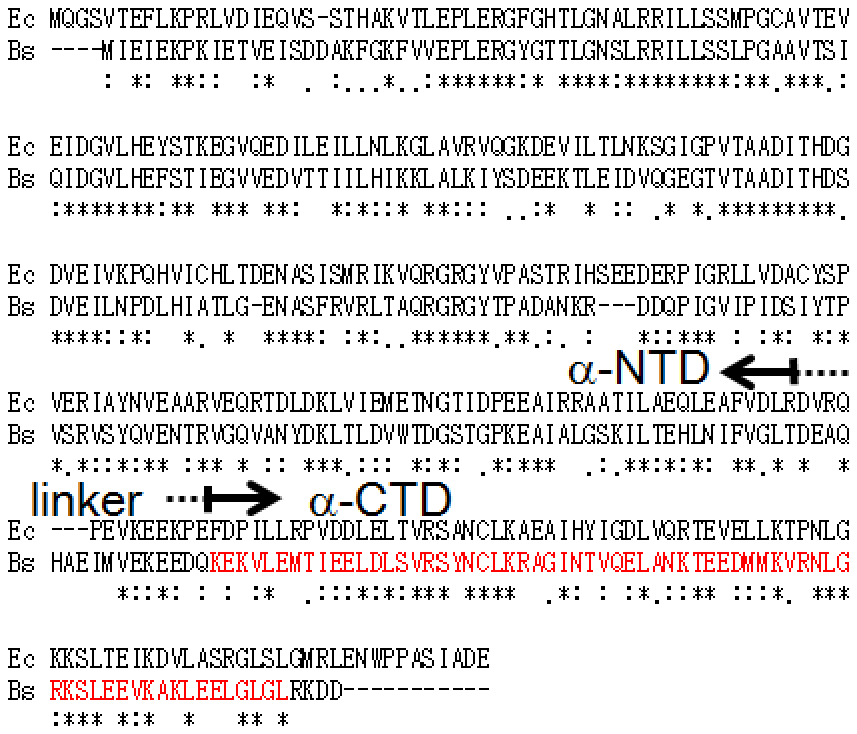

S1.Fig. Alignment of amino acid sequences for the *B. subtilis* (Bs) and *E. coli* (Ec) RpoA proteins. Alignment was performed using ClustalW. The boundary positions between the N-terminal domain (a-NTD) and the linker, and between the linker and the C-terminal domain (a-CTD) (as determined for *E. coli* RpoA [4]) are shown. Red letters represent the region deleted in the C-terminally truncated RpoA^{del}.

S1 Fig.
