# Supplementary material for: The Role of α-CTD in the Genome-Wide Transcriptional Regulation of the Bacillus subtilis Cells
Source: PLoS One. 2015 Jul 8;10(7):e0131588. doi: 10.1371/journal.pone.0131588 (PMC4495994; doi:10.1371/journal.pone.0131588)
Supplement: S2 Fig — RpoA del-expressing cells (SMS05; Pspac-rpoA int-rplQ, Pxyl-rpoA int -rplQ) were streaked on the left of each plate, and rpoA int-expressing cells (SMS06; Pspac-rpoA int-rplQ, Pxyl-rpoA del-rplQ) were streaked on the right. (PDF) [file pone.0131588.s002.pdf]

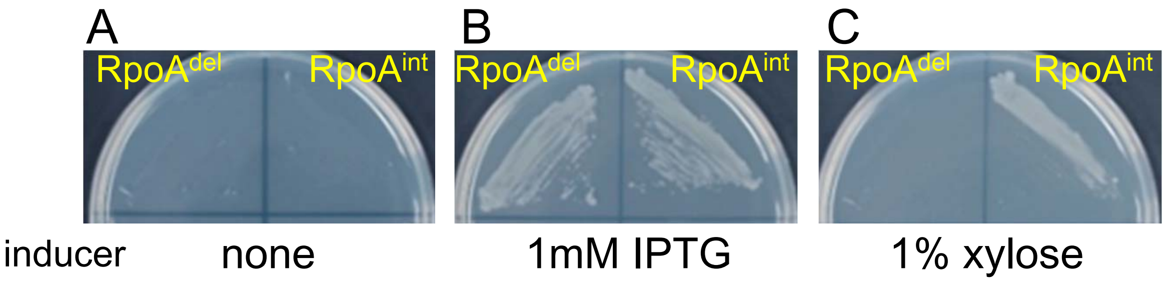

S2. Fig. Growth of *B. subtilis* cells streaked on LB plates without supplementation (A), or in the presence of 1 mM IPTG (B) or 1% xylose (C). *RpoA*^{del}-expressing cells (SMS05; P*spac-rpoA*^{int}-*rplQ*, P*xyl-rpoA*^{int}-*rplQ*) were streaked on the left of each plate, and *rpoA*^{int}-expressing cells (SMS06; P*spac-rpoA*^{int}-*rplQ*, P*xyl-rpoA*^{del}-*rplQ*) were streaked on the right.

S2 Fig.
