# Supplementary material for: The Role of α-CTD in the Genome-Wide Transcriptional Regulation of the Bacillus subtilis Cells
Source: PLoS One. 2015 Jul 8;10(7):e0131588. doi: 10.1371/journal.pone.0131588 (PMC4495994; doi:10.1371/journal.pone.0131588)
Supplement: S3 Fig — The crude lysates were prepared by the same procedure for the pull down assay. The amount of applied samples to SDS-PAGE were adjusted by the OD600 unit for cell culture. The duration of cultivation in LBxyl is shown at the bottom of each panel. (PDF) [file pone.0131588.s003.pdf]

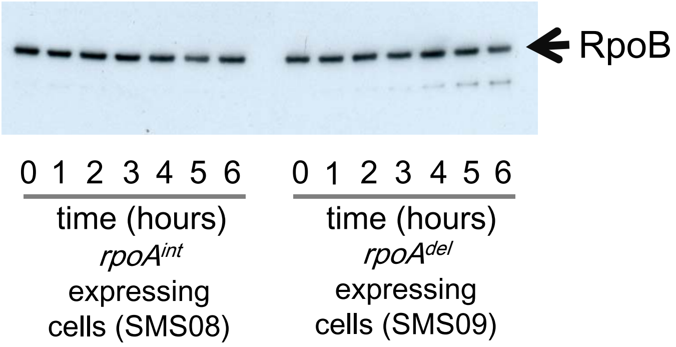

S3. Fig. Western blotting analysis of RNAP complex (RpoB) in crude extracts of *rpoA^{int}*-expressing cells (SMS08) and *rpoA^{del}*-expressing cells (SMS09). The crude lysates were prepared by the same procedure for the pull down assay. The amount of applied samples to SDS-PAGE were adjusted by the OD_{600} unit for cell culture. The duration of cultivation in LB^{xyl} is shown at the bottom of each panel.

S3 Fig.
