# Supplementary material for: The Role of α-CTD in the Genome-Wide Transcriptional Regulation of the Bacillus subtilis Cells
Source: PLoS One. 2015 Jul 8;10(7):e0131588. doi: 10.1371/journal.pone.0131588 (PMC4495994; doi:10.1371/journal.pone.0131588)
Supplement: S4 Fig — (A) Scatter plot of the RNAP binding intensity of each gene in rpoA del-expressing cells (vertical axis) versus rpoA int-expressing cells (horizontal axis). Genes identified in our transcriptome analysis as being up- and down-regulated in rpoA del-expressing cells at 3 hours after the beginning of the RpoAdel induction are shown as red and blue dots, respectively. The experiments were performed duplicate, and are shown as Exp. 1 and Exp. 2. The correlation coefficients for the RNAP binding intensities in rpoA int-expressing and rpoA del-expressing cells are indicated as (r) in each panel. (B) Distribution of the relative ratios of RNAP binding intensity (RpoC-His binding intensity) for each gene in rpoA int- and rpoA del-expressing cells (relative ratio = [RNAP binding intensity in rpoA int-expressing cells] / [RNAP binding intensity in rpoA int-expressing cells]). Three gene sets are shown, as assessed in rpoA del-expressing cells compared to rpoA int-expressing cells: all genes subjected to our ChAP-chip analysis (left); genes that were up-regulated in rpoA del-expressing cells per our transcriptome analysis at 3 hours after the beginning of the RpoAdel induction (middle); and genes that were down-regulated rpoA del-expressing cells per our transcriptome analysis at 3 hours after the beginning of the RpoAdel induction (right). Box plots represent the median (horizontal black lines), the upper and lower quartile values (boxes), and the most extreme data points within 1.5-fold interquartile ranges (whiskers). The asterisks indicate statistically significant differences between the gene sets, as assessed by the Wilcoxon rank-sum test (**: p-value < 0.01). (PDF) [file pone.0131588.s004.pdf]

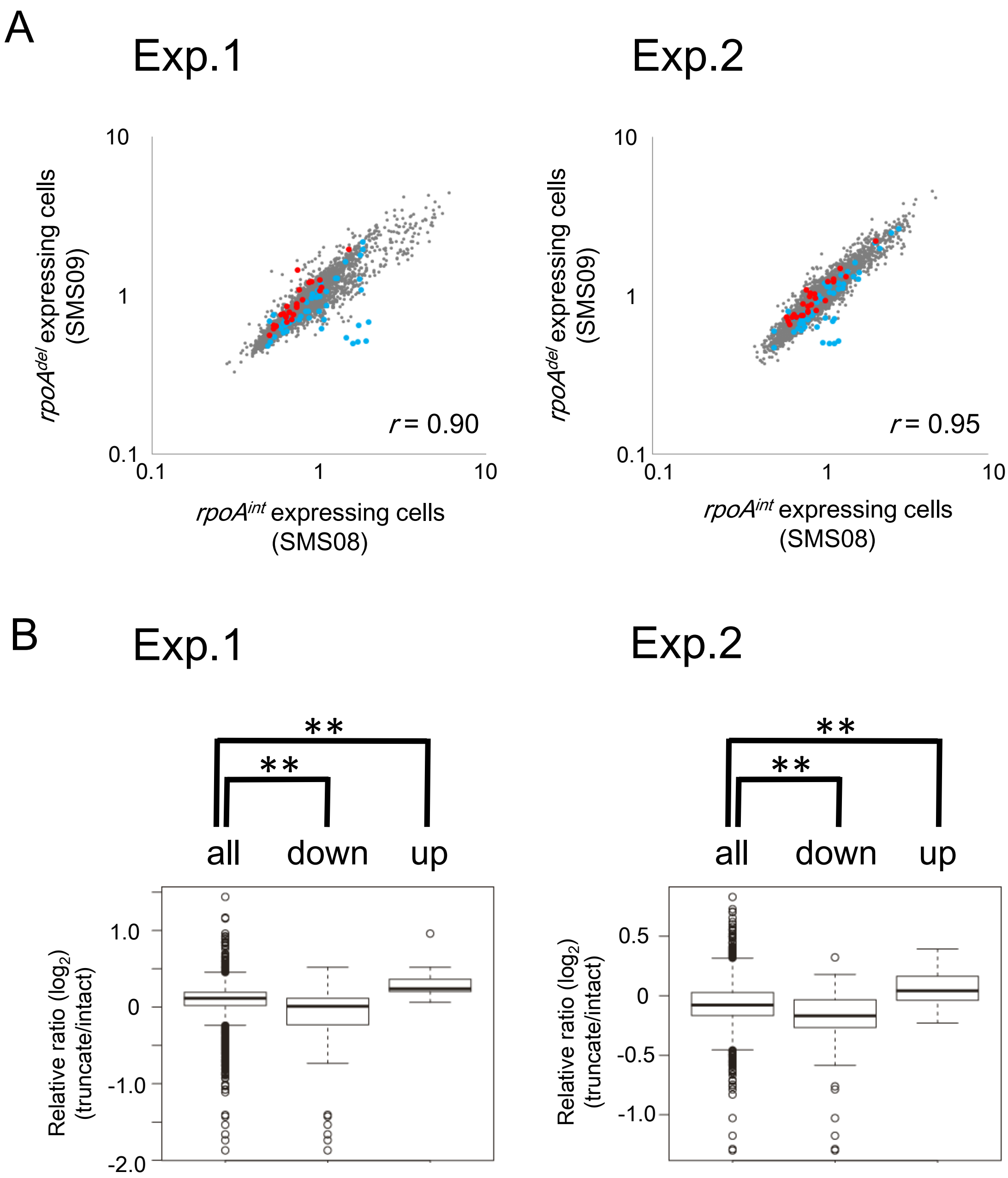
A
Exp.1
Exp.2
10
1
0.1
0.1
1
10
rpoA^del expressing cells (SMS09)
rpoA^int expressing cells (SMS08)
r = 0.90
r = 0.95
B
Exp.1
Exp.2
**
**
all
down
up
1.0
0
-1.0
-2.0
0.5
-0.5
Relative ratio (log2) (truncate/intact)

S4 Fig.

S4.Fig. ChAP-chip analysis of RNAP (RpoC-His) in *rpoA^{int}*-expressing cells (SMS08) and *rpoA^{del}*-expressing cells (SMS09). (A) Scatter plot of the RNAP binding intensity of each gene in *rpoA^{del}*-expressing cells (vertical axis) versus *rpoA^{int}*-expressing cells (horizontal axis). Genes identified in our transcriptome analysis as being up- and down-regulated in *rpoA^{del}*-expressing cells at 3 hours after the beginning of the RpoA^{del} induction are shown as red and blue dots, respectively. The experiments were performed duplicate, and are shown as Exp. 1 and Exp. 2. The correlation coefficients for the RNAP binding intensities in *rpoA^{int}*-expressing and *rpoA^{del}*-expressing cells are indicated as (*r*) in each panel. (B) Distribution of the relative ratios of RNAP binding intensity (RpoC-His binding intensity) for each gene in *rpoA^{int}*- and *rpoA^{del}*-expressing cells (relative ratio = [RNAP binding intensity in *rpoA^{int}*-expressing cells] / [RNAP binding intensity in *rpoA^{int}*-expressing cells]). Three gene sets are shown, as assessed in *rpoA^{del}*-expressing cells compared to *rpoA^{int}*-expressing cells: all genes subjected to our ChAP-chip analysis (left); genes that were up-regulated in *rpoA^{del}*-expressing cells per our transcriptome analysis at 3 hours after the beginning of the RpoA^{del} induction (middle); and genes that were down-regulated *rpoA^{del}*-expressing cells per our transcriptome analysis at 3 hours after the beginning of the RpoA^{del} induction (right). Box plots represent the median (horizontal black lines), the upper and lower quartile values (boxes), and the most extreme data points within 1.5-fold interquartile ranges (whiskers). The asterisks indicate statistically significant differences between the gene sets, as assessed by the Wilcoxon rank-sum test (**: *p*-value < 0.01).

S4 Fig.
