# Supplementary material for: The Role of α-CTD in the Genome-Wide Transcriptional Regulation of the Bacillus subtilis Cells
Source: PLoS One. 2015 Jul 8;10(7):e0131588. doi: 10.1371/journal.pone.0131588 (PMC4495994; doi:10.1371/journal.pone.0131588)
Supplement: S5 Fig — Scatter plots of the RpoA binding intensity for each gene in SMS19 cells (vertical axis) versus SMS18 cells (horizontal axis) in duplicate experiments. The correlation coefficients of the RNAP binding intensities in SMS18 and SMS19 cells are indicated as (r) in each panel. (PDF) [file pone.0131588.s005.pdf]

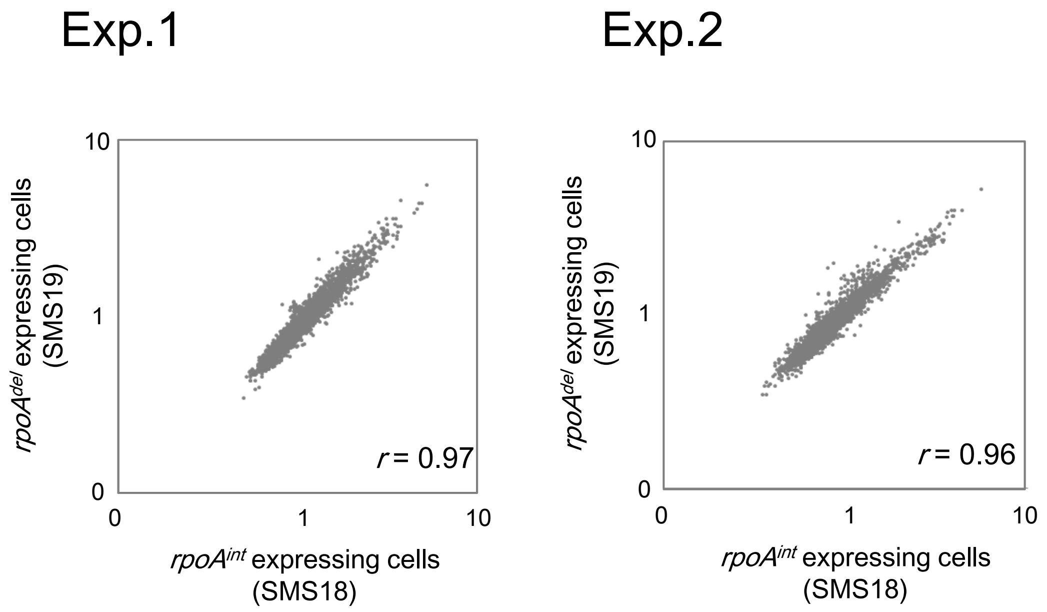

S5.Fig. ChAP-chip analysis of RpoA in *rpoA^int*-expressing cells (SMS18) and *rpoA^del*-expressing cells (SMS19). Scatter plots of the RpoA binding intensity for each gene in SMS19 cells (vertical axis) versus SMS18 cells (horizontal axis) in duplicate experiments. The correlation coefficients of the RNAP binding intensities in SMS18 and SMS19 cells are indicated as (*r*) in each panel.

S5 Fig.
