# Supplementary material for: The Role of α-CTD in the Genome-Wide Transcriptional Regulation of the Bacillus subtilis Cells
Source: PLoS One. 2015 Jul 8;10(7):e0131588. doi: 10.1371/journal.pone.0131588 (PMC4495994; doi:10.1371/journal.pone.0131588)
Supplement: S6 Fig — The results are shown as described for Fig 5. The thick blue arrow at the top of the figure indicates the genes most highly reduced in RNAP binding. Arrow heads in S6 Fig (8 and 9) indicate “peaks” of RNAP observed at promoter or promoter proximal regions in rpoA del expressing cells. (PDF) [file pone.0131588.s006.pdf]

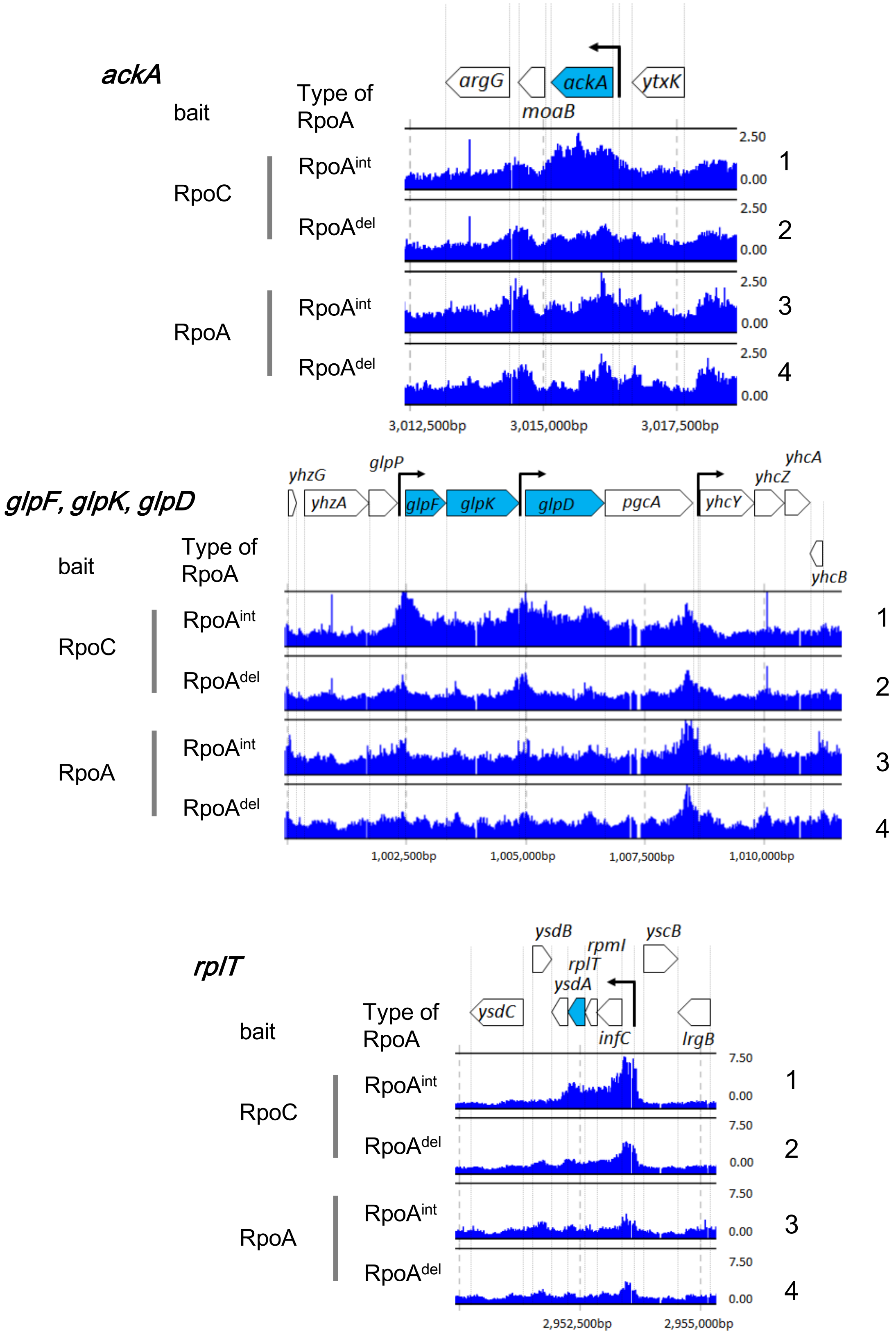
ackA
bait
Type of RpoA
argG
ackA
ytxK
moaB
RpoC
RpoA^int
RpoA^del
RpoA
RpoA^int
RpoA^del
2.50
0.00
1
2
3
4
3,012,500bp
3,015,000bp
3,017,500bp
glpF, glpK, glpD
yhzG
glpP
yhcA
yhzA
glpF
glpK
glpD
pgcA
yhcY
yhcZ
yhcB
bait
Type of RpoA
RpoC
RpoA^int
RpoA^del
RpoA
RpoA^int
RpoA^del
1,002,500bp
1,005,000bp
1,007,500bp
1,010,000bp
rplT
ysdB
rpmI
yscB
rplT
ysdA
ysdC
infC
lrgB
bait
Type of RpoA
RpoC
RpoA^int
RpoA^del
RpoA
RpoA^int
RpoA^del
7.50
0.00
2,952,500bp
2,955,000bp

S6-1 Fig.

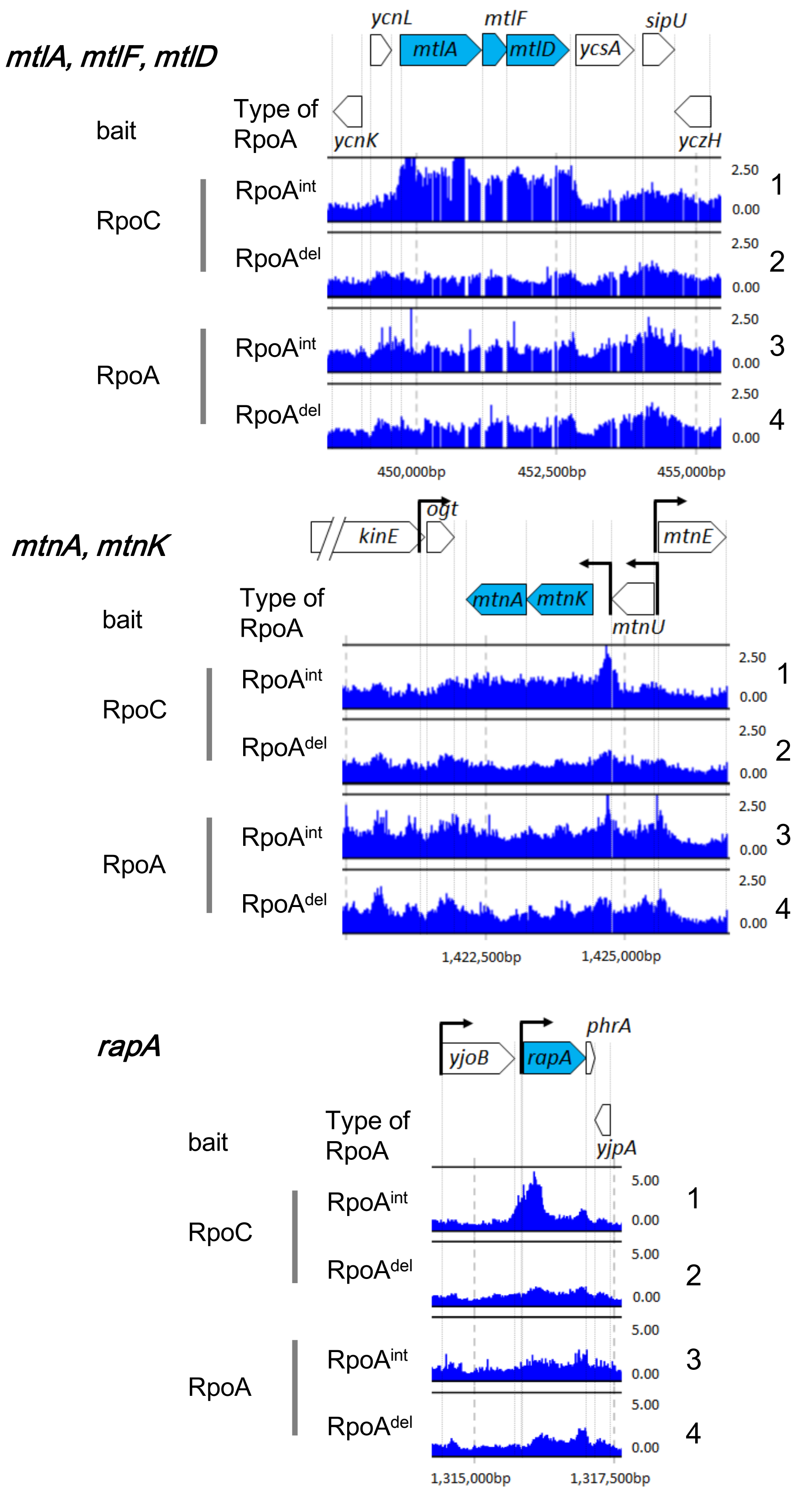
mtlA, mtlF, mtlD
ycnL
mtlF
sipU
mtlA
mtlD
ycsA
bait
Type of RpoA
ycnK
yczH
RpoC
RpoA
RpoAint
RpoAdel
RpoAint
RpoAdel
2.50
0.00
1
2
3
4
450,000bp
452,500bp
455,000bp
mtnA, mtnK
kinE
ogt
mtnE
mtnA
mtnK
mtnU
1,422,500bp
1,425,000bp
rapA
yjoB
rapA
phrA
yjpA
5.00
1,315,000bp
1,317,500bp

S6-2 Fig.

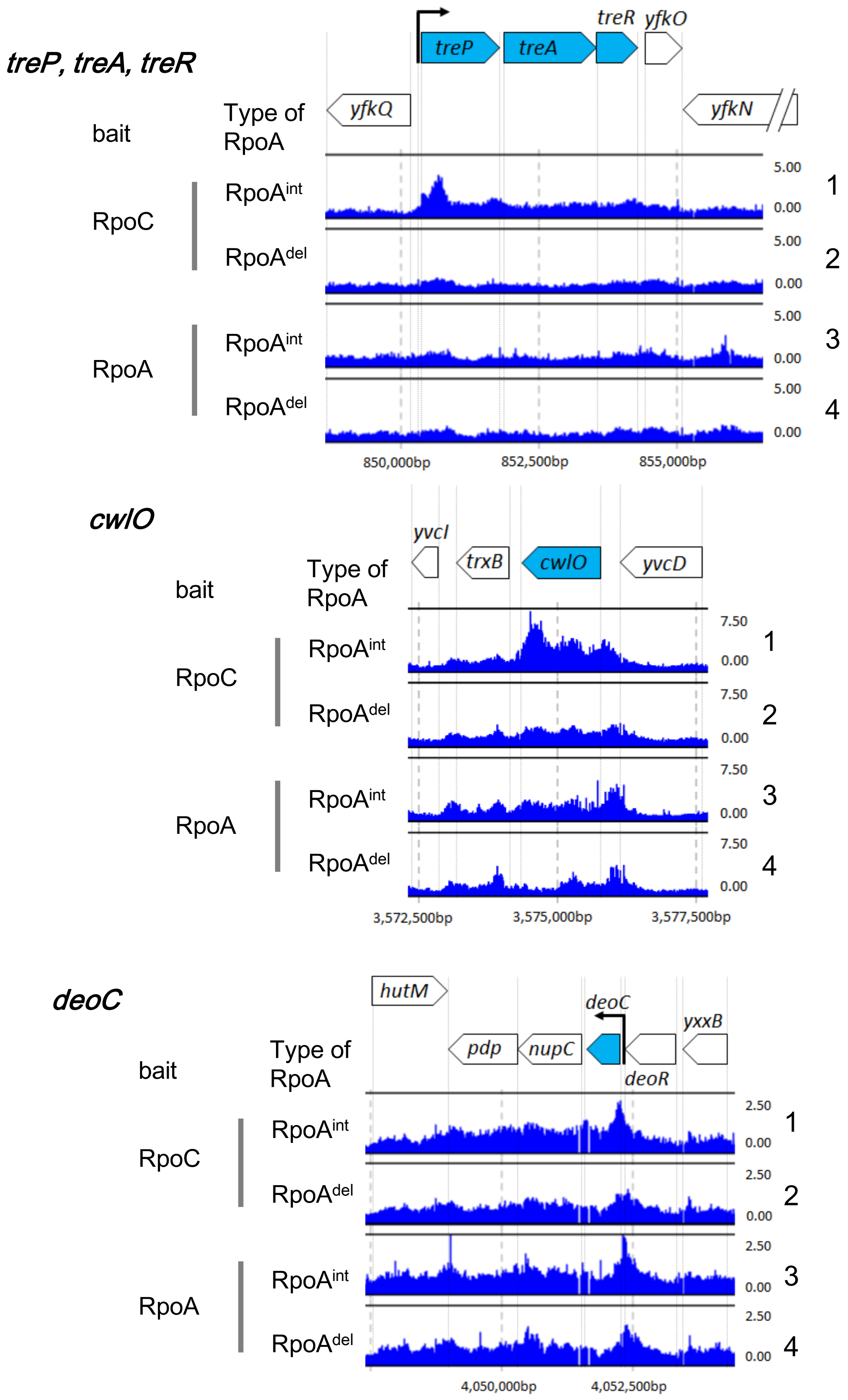

treP, treA, treR
bait
Type of RpoA
RpoC
RpoA
RpoA^int
RpoA^del
treP
treA
treR
yfkO
yfkQ
yfkN
5.00
0.00
1
2
3
4
850,000bp
852,500bp
855,000bp
cwlO
yvcI
trxB
cwlO
yvcD
7.50
0.00
3,572,500bp
3,575,000bp
3,577,500bp
deoC
hutM
pdp
nupC
deoC
deoR
yxxB
2.50
0.00
4,050,000bp
4,052,500bp

S6-3 Fig.

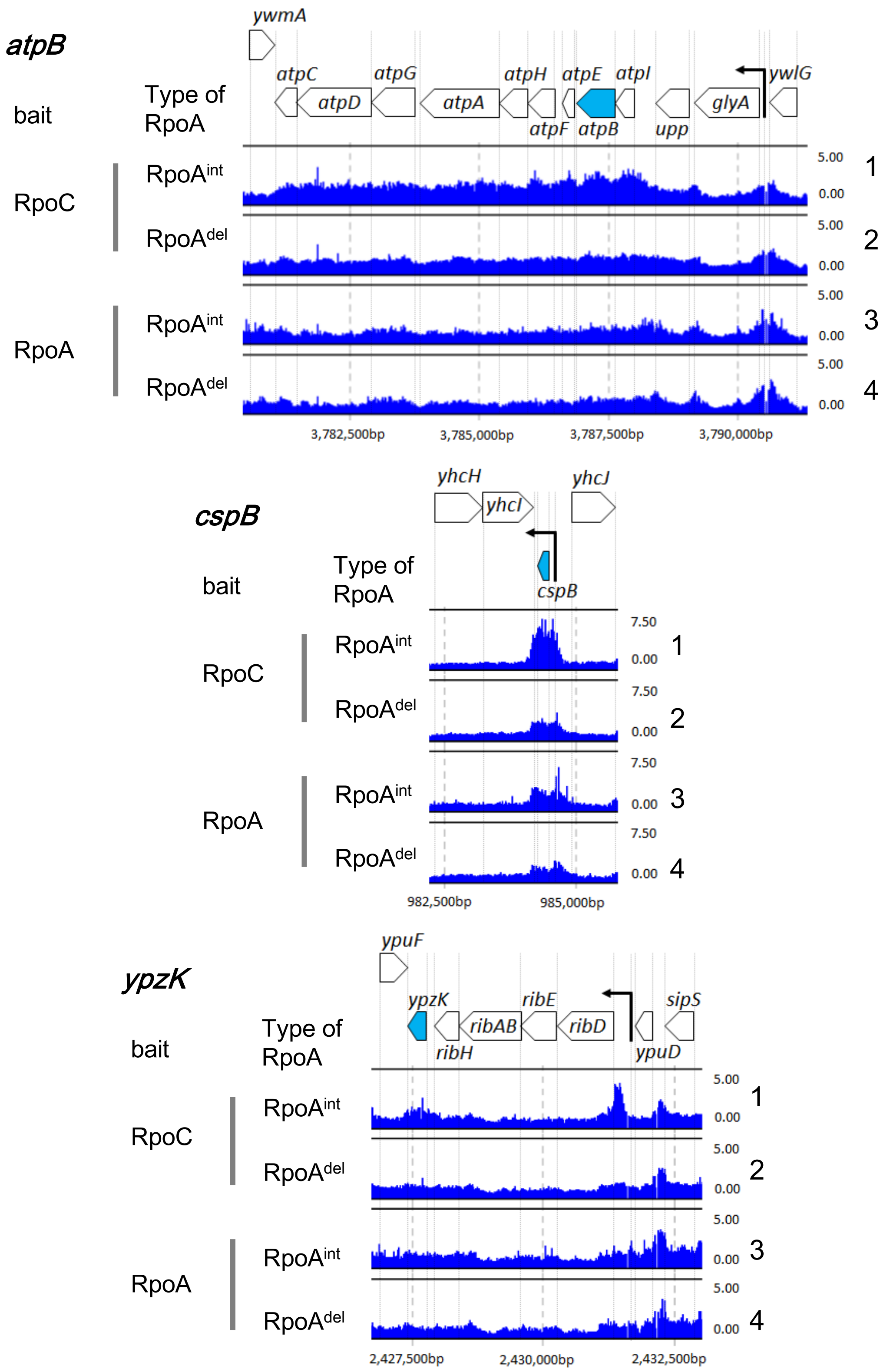

atpB
bait
Type of RpoA
ywmA
atpC
atpD
atpG
atpA
atpH
atpE
atpI
atpF
atpB
upp
glyA
ywlG
RpoC
RpoA
RpoAint
RpoAdel
RpoAint
RpoAdel
5.00
0.00
1
2
3
4
3,782,500bp
3,785,000bp
3,787,500bp
3,790,000bp
cspB
bait
Type of RpoA
yhcH
yhcI
yhcJ
cspB
RpoC
RpoA
RpoAint
RpoAdel
RpoAint
RpoAdel
7.50
0.00
1
2
3
4
982,500bp
985,000bp
ypzK
bait
Type of RpoA
ypuF
ypzK
ribE
sipS
ribAB
ribD
ribH
ypuD
RpoC
RpoA
RpoAint
RpoAdel
RpoAint
RpoAdel
5.00
0.00
1
2
3
4
2,427,500bp
2,430,000bp
2,432,500bp

S6-4 Fig.

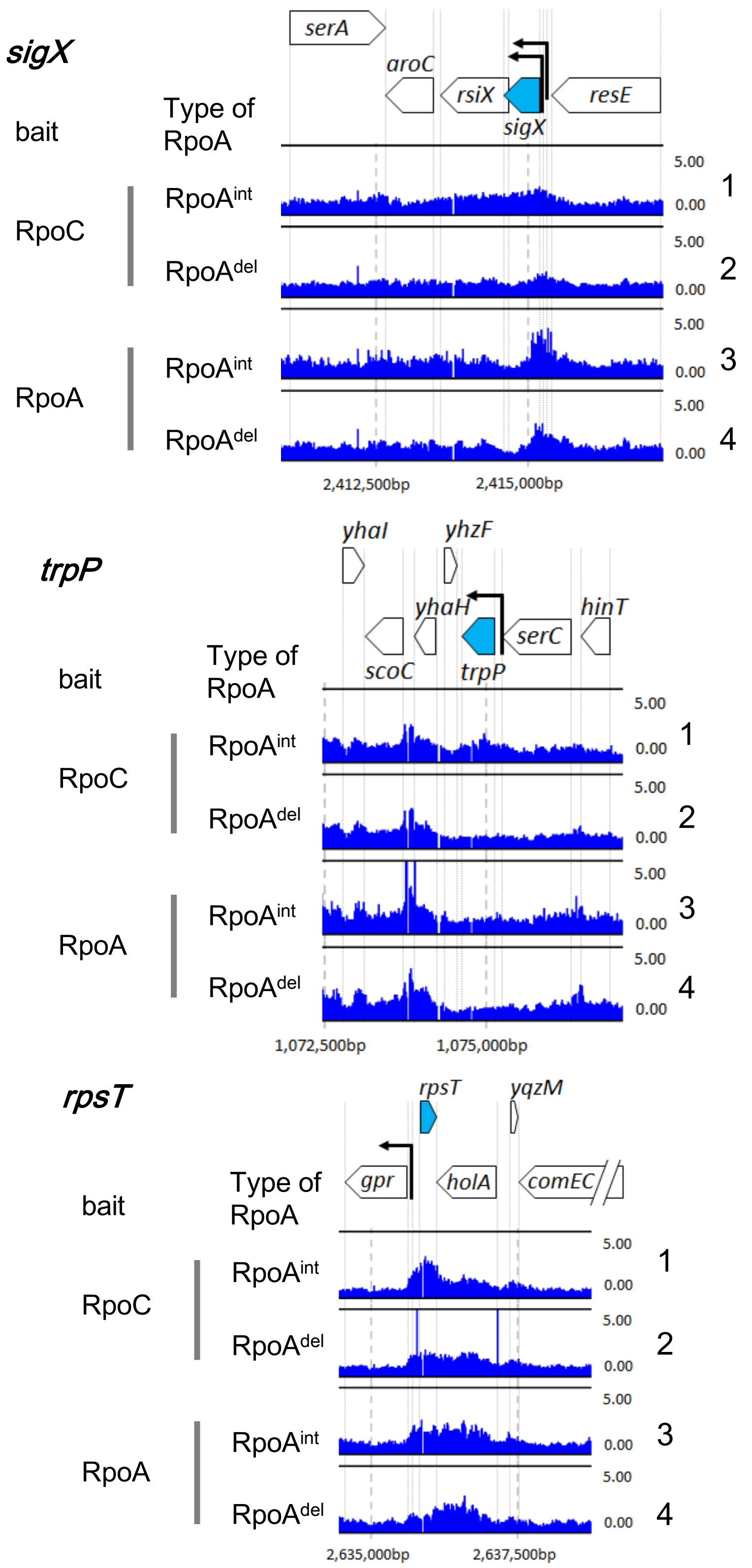

sigX
serA
aroC
rsiX
sigX
resE
bait
Type of RpoA
RpoC
RpoA^int
RpoA^del
RpoA
RpoA^int
RpoA^del
5.00
0.00
1
2
3
4
2,412,500bp
2,415,000bp
trpP
yhaI
yhzF
yhaH
hinT
serC
scoC
trpP
1,072,500bp
1,075,000bp
rpsT
rpsT
yqzM
gpr
holA
comEC
2,635,000bp
2,637,500bp

S6-5 Fig.

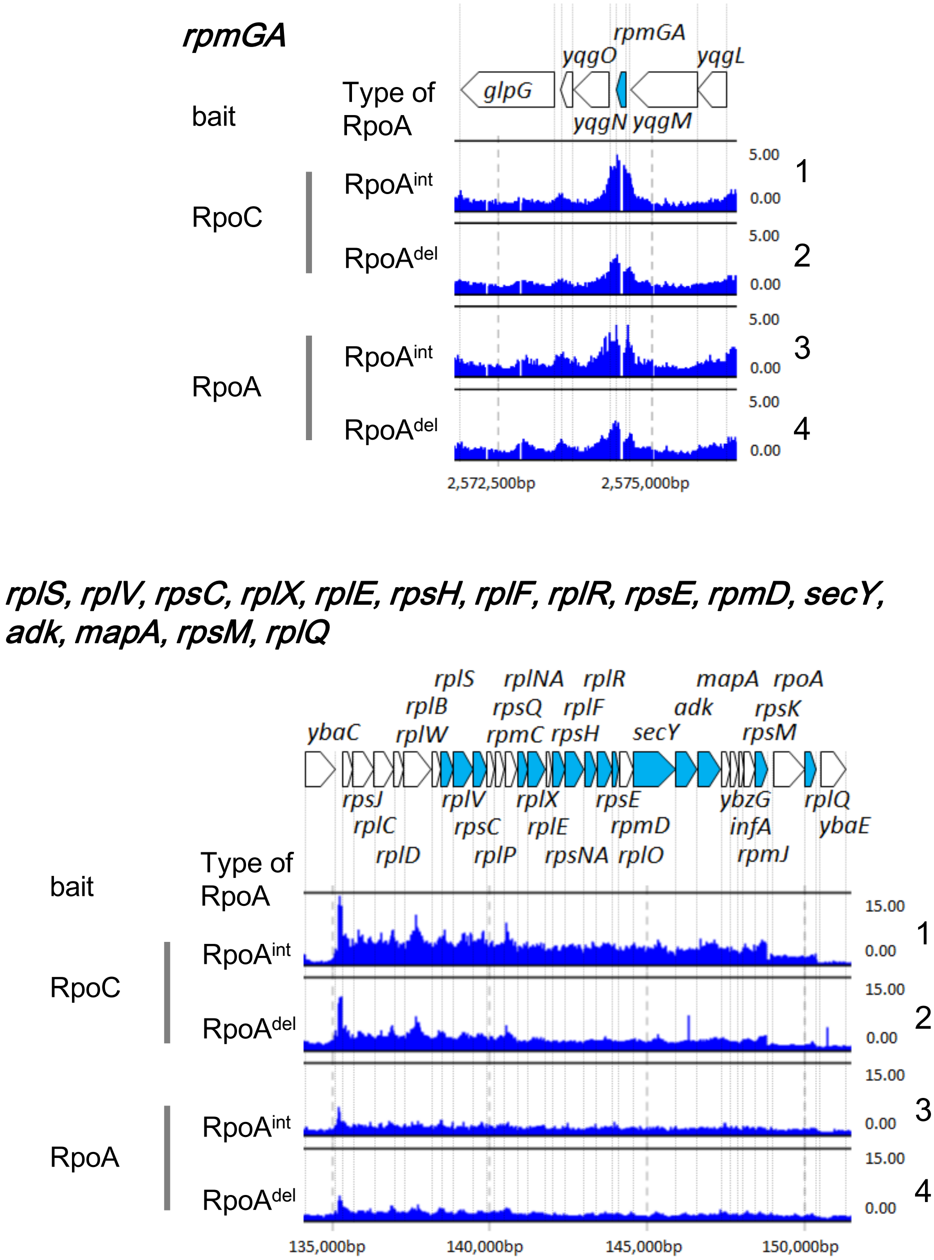
rpmGA
bait
Type of RpoA
glpG
yqgO
rpmGA
yqgL
yqgN
yqgM
RpoC
RpoA
RpoAint
RpoAdel
RpoAint
RpoAdel
5.00
0.00
1
2
3
4
2,572,500bp
2,575,000bp
rplS, rplV, rpsC, rplX, rplE, rpsH, rplF, rplR, rpsE, rpmD, secY, adk, mapA, rpsM, rplQ
rplS
rplNA
rplR
mapA
rpoA
rplB
rpsQ
rplF
adk
rpsK
ybaC
rplW
rpmC
rpsH
secY
rpsM
rpsJ
rplV
rplX
rpsE
ybzG
rplQ
rplC
rpsC
rplE
rpmD
infA
ybaE
rplD
rplP
rpsNA
rplO
rpmJ
bait
Type of RpoA
RpoC
RpoA
RpoAint
RpoAdel
RpoAint
RpoAdel
15.00
0.00
1
2
3
4
135,000bp
140,000bp
145,000bp
150,000bp

S6-6 Fig.

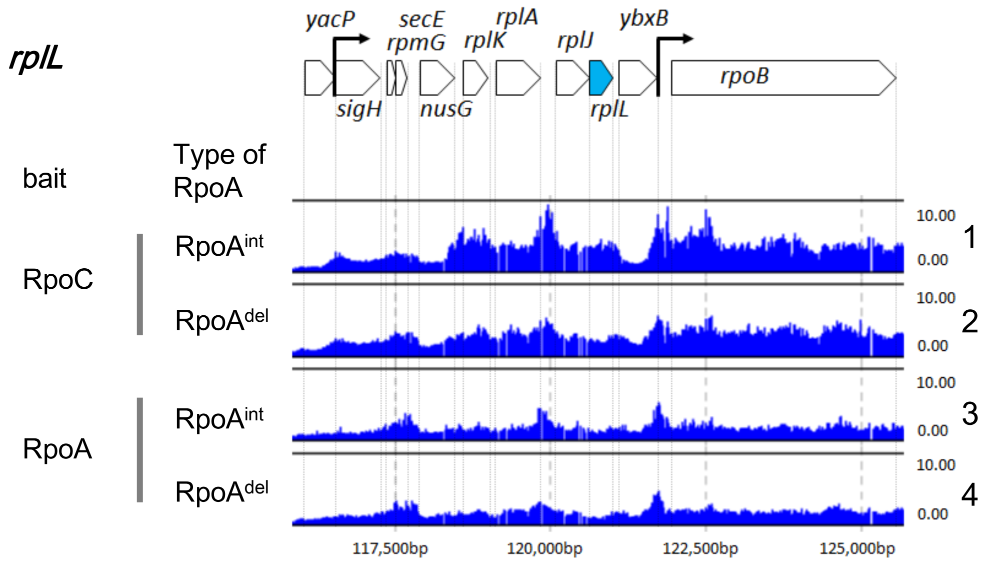
rplL
yacP
secE
rpmG
rplA
rplK
rplJ
ybxB
rpoB
sigH
nusG
rplL
bait
Type of RpoA
RpoC
RpoA^int
RpoA^del
RpoA
RpoA^int
RpoA^del
10.00
0.00
1
2
3
4
117,500bp
120,000bp
122,500bp
125,000bp

S6-7 Fig.

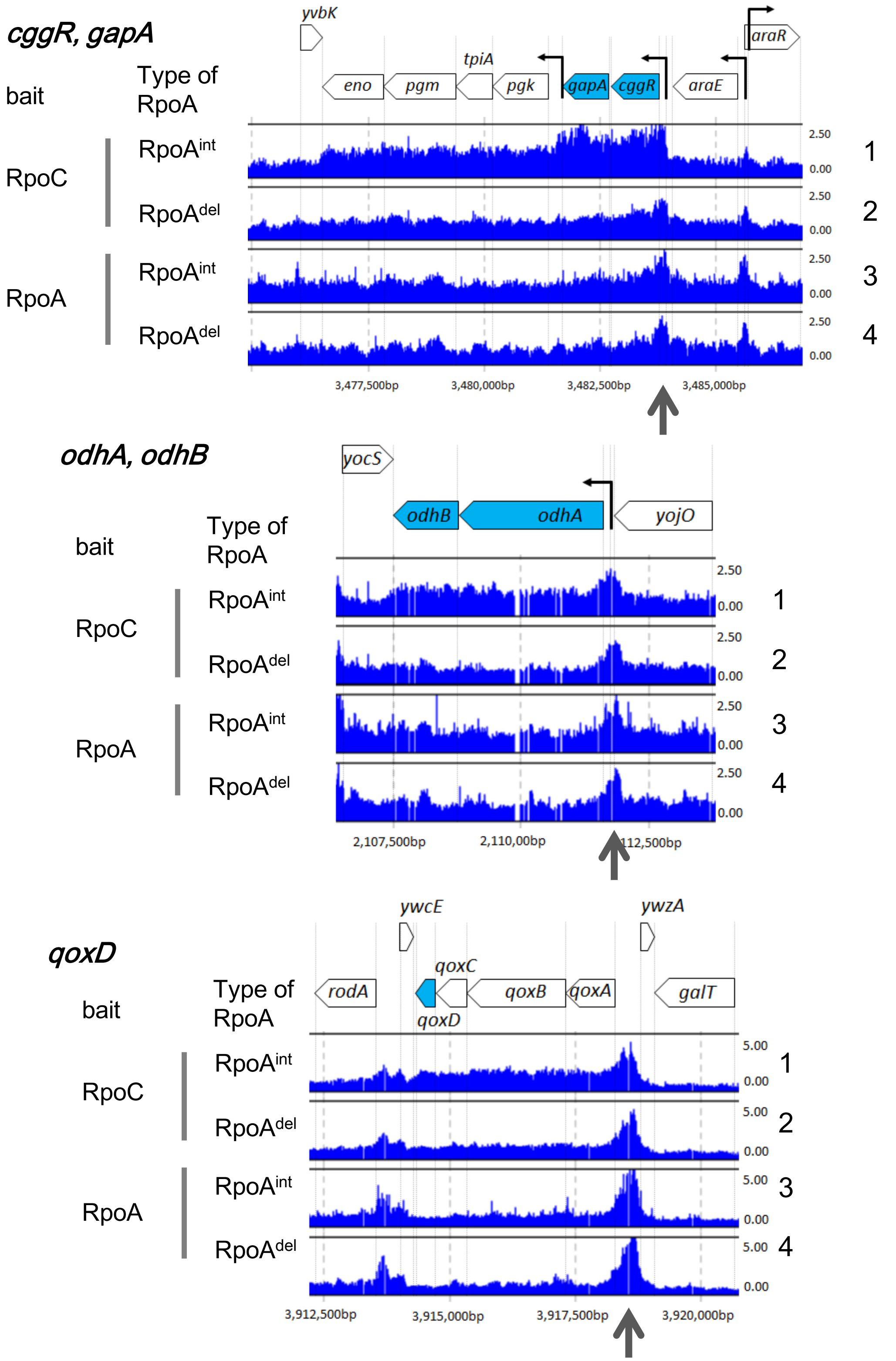

cggR, gapA
bait
Type of RpoA
yvbK
tpiA
eno
pgm
pgk
gapA
cggR
araE
araR
RpoC
RpoA
RpoAint
RpoAdel
RpoAint
RpoAdel
2.50
0.00
1
2
3
4
3,477,500bp
3,480,000bp
3,482,500bp
3,485,000bp
odhA, odhB
bait
Type of RpoA
yocS
odhB
odhA
yojO
RpoC
RpoA
RpoAint
RpoAdel
RpoAint
RpoAdel
2.50
0.00
1
2
3
4
2,107,500bp
2,110,00bp
112,500bp
qoxD
bait
Type of RpoA
ywcE
ywzA
rodA
qoxC
qoxB
qoxA
qoxD
galT
RpoC
RpoA
RpoAint
RpoAdel
RpoAint
RpoAdel
5.00
0.00
1
2
3
4
3,912,500bp
3,915,000bp
3,917,500bp
3,920,000bp

S6-8 Fig.

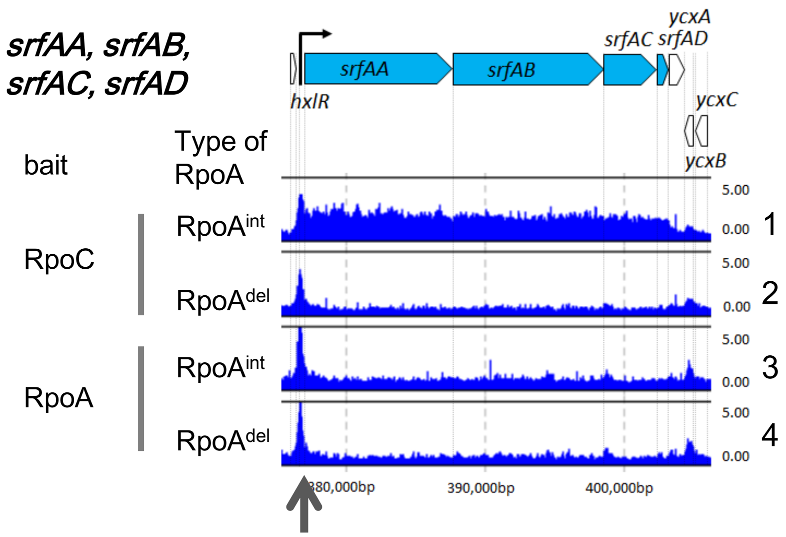

S6. Fig. RNAP binding profiles of the top 50 genes showing the highest reductions in RNAP binding. The results are shown as described for Fig. 5. The thick blue arrow at the top of the figure indicates the genes most highly reduced in RNAP binding. Arrow heads in Fig. S6-8 and -9 indicate "peaks" of RNAP observed at promoter or promoter proximal regions in *rpoA^{del}* expressing cells.
