# Supplementary material for: The Role of α-CTD in the Genome-Wide Transcriptional Regulation of the Bacillus subtilis Cells
Source: PLoS One. 2015 Jul 8;10(7):e0131588. doi: 10.1371/journal.pone.0131588 (PMC4495994; doi:10.1371/journal.pone.0131588)
Supplement: S7 Fig — (A) The scatter plot is same with Fig 3D. The dark-blue dots indicate genes most highly reduced RNAP binding (identified by ChAP-chip analysis). The sky-blue dots indicate genes whose transcription were down-regulated in rpoA del- expressing cells (identified by transcriptome analysis). The pink dots represent genes highly reduced RNAP binding and down-regulated in rpoA del- expressing cells (identified by both ChAP-chip and transcriptome analysis). (B) Enlarged view of the area containing the genes that showed the highest signal intensities in our transcriptome analysis. (PDF) [file pone.0131588.s007.pdf]

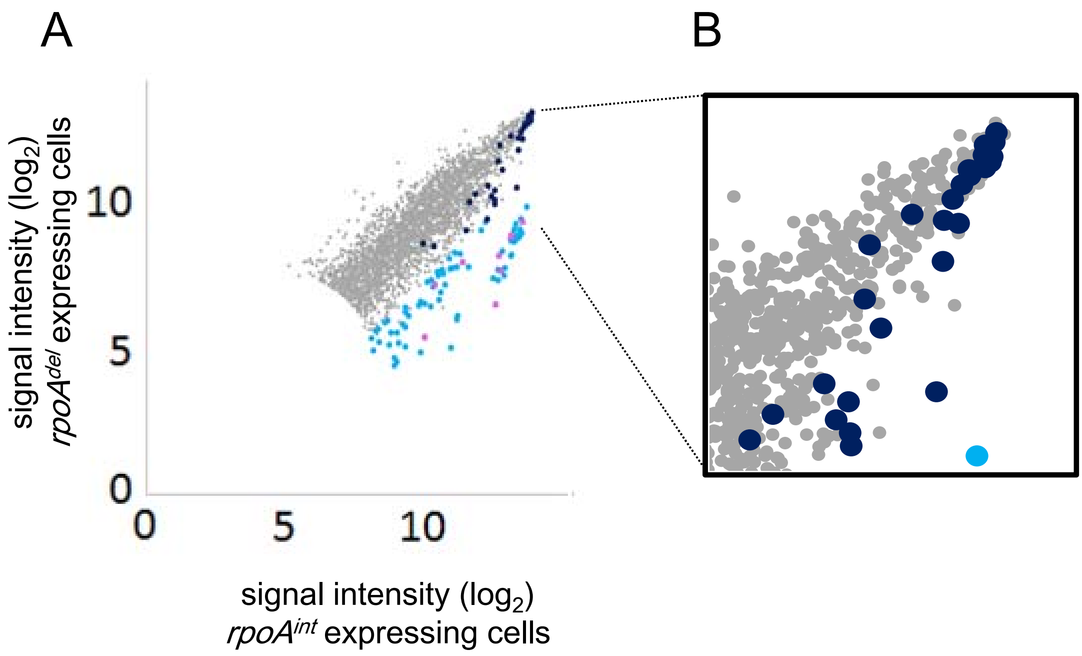

S7. Fig. The distribution of the genes most highly reduced in their RNAP binding identified by ChAP-chip analysis in the scatter plot of the transcriptional signal intensity of each gene in *rpoA*^{del}- and *rpoA*^{int}-expressing cells. (A) The scatter plot is same with Figure 3D. The dark-blue dots indicate genes most highly reduced RNAP binding (identified by ChAP-chip analysis). The sky-blue dots indicate genes whose transcription were down-regulated in *rpoA*^{del}- expressing cells (identified by transcriptome analysis). The pink dots represent genes highly reduced RNAP binding and down-regulated in *rpoA*^{del}- expressing cells (identified by both ChAP-chip and transcriptome analysis). (B) Enlarged view of the area containing the genes that showed the highest signal intensities in our transcriptome analysis.

S7 Fig.
