# Supplementary material for: The Role of α-CTD in the Genome-Wide Transcriptional Regulation of the Bacillus subtilis Cells
Source: PLoS One. 2015 Jul 8;10(7):e0131588. doi: 10.1371/journal.pone.0131588 (PMC4495994; doi:10.1371/journal.pone.0131588)
Supplement: S8 Fig — The sequences spanning from -41 to -57 are compared with the UP element consensus sequence. The conserved nucleotides are shown in red letters. The difference between each candidate sequence and the consensus sequence is shown at the right of the figure. The clpE and mtnX genes have two promoters for each gene; in these cases, the “up” notation indicates the upstream promoter. (PDF) [file pone.0131588.s008.pdf]

gene	UP element	deference
wapA	AGATAAATTTTCTAGAA	3bp
yoeB	AAAATAATTTATGCAAA	3bp
gmuB	TTATATTTATTTTATAA	4bp
rbsC	AGATTTCTTTTGATATT	5bp
$clpE^{up}$	CAAAAATTTTTGTGCAT	5bp
spo0E	ACTTATTTAATGAAAAT	5bp
maeN	TTTATTAGTTTTTTAAC	5bp
gamA	CAAATTTTCAGAAATTA	6bp
mtnA	GATTTATATTAAAAAAT	6bp
$mtnX^{up}$	ACCTGTTTACCCAAAAA	6bp
cspB	CGAAAAAAATTTCAATA	6bp
sigX	GGATATGTTAATATAAA	6bp

consensus

AAA$^{A}_{T}$$^{A}_{T}T^{A}_{T}$TTTT- - AAAA

S8. Fig. Candidate UP elements for known promoters of 12 genes found to be down-regulated and/or highly reduced RNAP binding in $rpoA^{del}$-expressing cells. The sequences spanning from -41 to -57 are compared with the UP element consensus sequence. The conserved nucleotides are shown in red letters. The difference between each candidate sequence and the consensus sequence is shown at the right of the figure. The *clpE* and *mtnX* genes have two promoters for each gene; in these cases, the “up” notation indicates the upstream promoter.

S8 Fig.
