# Supplementary material for: The Role of α-CTD in the Genome-Wide Transcriptional Regulation of the Bacillus subtilis Cells
Source: PLoS One. 2015 Jul 8;10(7):e0131588. doi: 10.1371/journal.pone.0131588 (PMC4495994; doi:10.1371/journal.pone.0131588)
Supplement: S9 Fig — Data on the transcriptional start sites (TSSs) were taken from Irnov et al. (2010) [51]. The DNA sequences upstream of each promoter (-38 to -57 bp) were acquired and numbered with respect to the TSS (bottom of bar graphs). The nucleotide frequency in each position was indicated as bar graphs. The nucleotide conservation at each position analyzed using the Weblogo program (http://weblogo.threeplusone.com/) [52] was indicated under bar graphs. The locations of the proximal and distal portions of the putative UP elements are indicated in figures of logo. We identified 25 TSSs corresponding to the genes identified as showing down-regulation (transcriptomic analysis) and highly decreased RNAP binding (ChAP-chip analysis) in rpoA del-expressing cells: cggR, cspB, cwlO, deoC, glpD, glpF, odhA, rapA, rpmGA, rpsT, srfAA, trpP, ypzK, comQ, pel, rapC, spo0E, wapA, xpt, yrhP, ywsB, iseA, yisT, yuiF and yxbC. (A) The nucleotide frequency and conservation in the DNA sequences upstream of 25 promoters (-38 to -57 bp) for the genes down-regulated and highly decreased RNAP binding in rpoA del-expressing cells. (B) The nucleotide frequency and conservation in the DNA sequences upstream of the other tested genes. (PDF) [file pone.0131588.s009.pdf]

A For the genes down-regulated and highly decreased RNAP binding in *rpoA*[del]-expressing cells (25 genes)

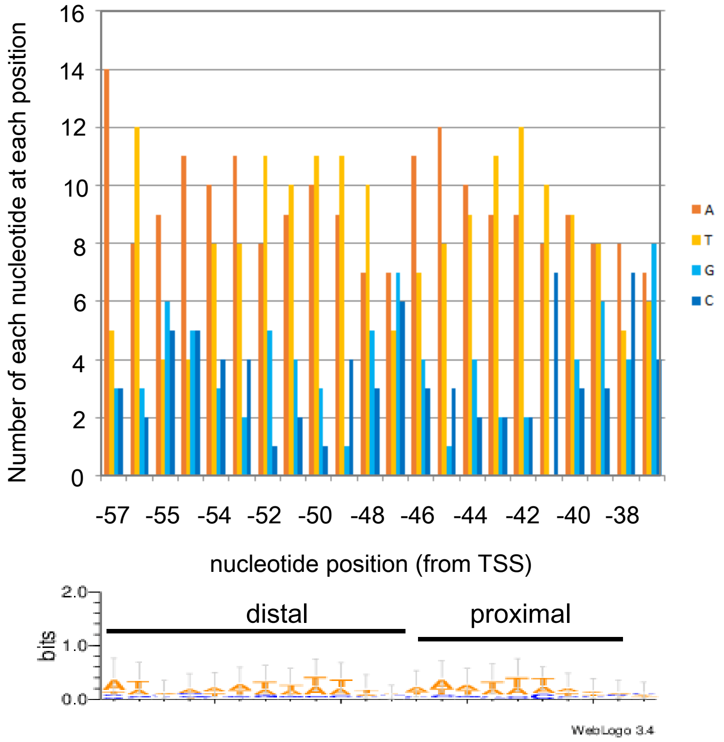

B For the other genes (575 genes)

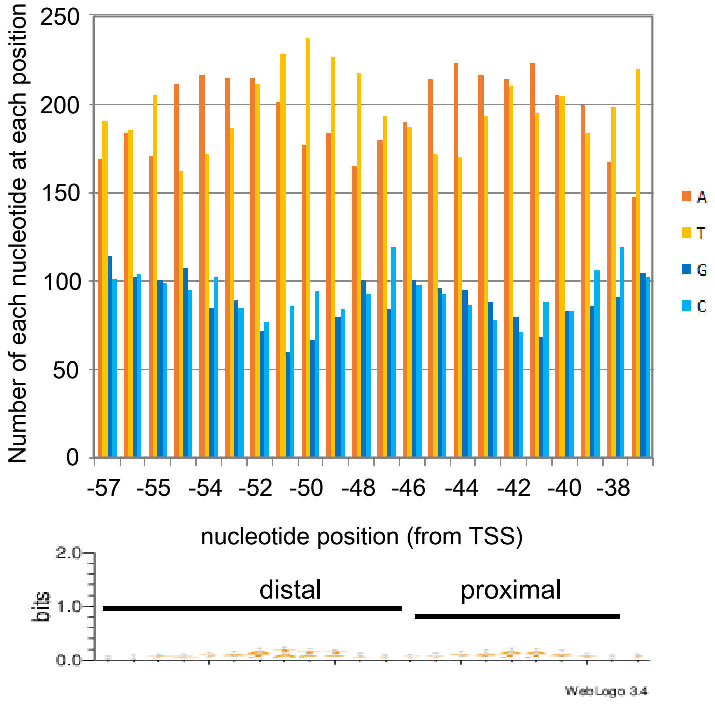

S9 Fig.

S9. Fig. The analysis of upstream regions of the -35 elements of promoters for genes that are down-regulated and/or highly reduced RNAP binding in *rpoA^del^*-expressing cells (SMS09) compared with *rpoA^int^*-expressing cells (SMS08). Data on the transcriptional start sites (TSSs) were taken from Irnov et al. (2010) [49]. The DNA sequences upstream of each promoter (-38 to -57 bp) were acquired and numbered with respect to the TSS (bottom of bar graphs). The nucleotide frequency in each position was indicated as bar graphs. The nucleotide conservation at each position analyzed using the Weblogo program (http://weblogo.threeplusone.com/) [50] was indicated under bar graphs. The locations of the proximal and distal portions of the putative UP elements are indicated in figures of logo. We identified 25 TSSs corresponding to the genes identified as showing down-regulation (transcriptomic analysis) and highly decreased RNAP binding (ChAP-chip analysis) in *rpoA^del^*-expressing cells: *cggR, cspB, cwlO, deoC, glpD, glpF, odhA, rapA, rpmGA, rpsT, srfAA, trpP, ypzK, comQ, pel, rapC, spo0E, wapA, xpt, yrhP, ywsB*, *iseA*, *yisT*, *yuiF* and *yxbC*. (A) The nucleotide frequency and conservation in the DNA sequences upstream of 25 promoters (-38 to -57 bp) for the genes down-regulated and highly decreased RNAP binding in *rpoA^del^*-expressing cells. (B) The nucleotide frequency and conservation in the DNA sequences upstream of the other tested genes.

S9 Fig.
