# Supplementary material for: The Role of α-CTD in the Genome-Wide Transcriptional Regulation of the Bacillus subtilis Cells
Source: PLoS One. 2015 Jul 8;10(7):e0131588. doi: 10.1371/journal.pone.0131588 (PMC4495994; doi:10.1371/journal.pone.0131588)
Supplement: S10 Fig — RpoA int -His, RpoA del -His and RpoC-His were used as bait for the purification of RNAP complexes. The gene arrangement on the B. subtilis chromosome is shown by thick arrows at the top of the figure. Colors: sky blue indicates genes that are down-regulated in rpoA del-expressing cells compared with rpoA int-expressing cells, as determined by transcriptomic analysis; green indicates the top 50 genes showing the greatest reductions in RNAP binding among rpoA del-expressing cells versus rpoA int-expressing cells; and dark blue indicates the genes that showed both transcriptomic down-regulation and high-level reductions in RNAP binding in rpoA del-expressing cells. (PDF) [file pone.0131588.s010.pdf]

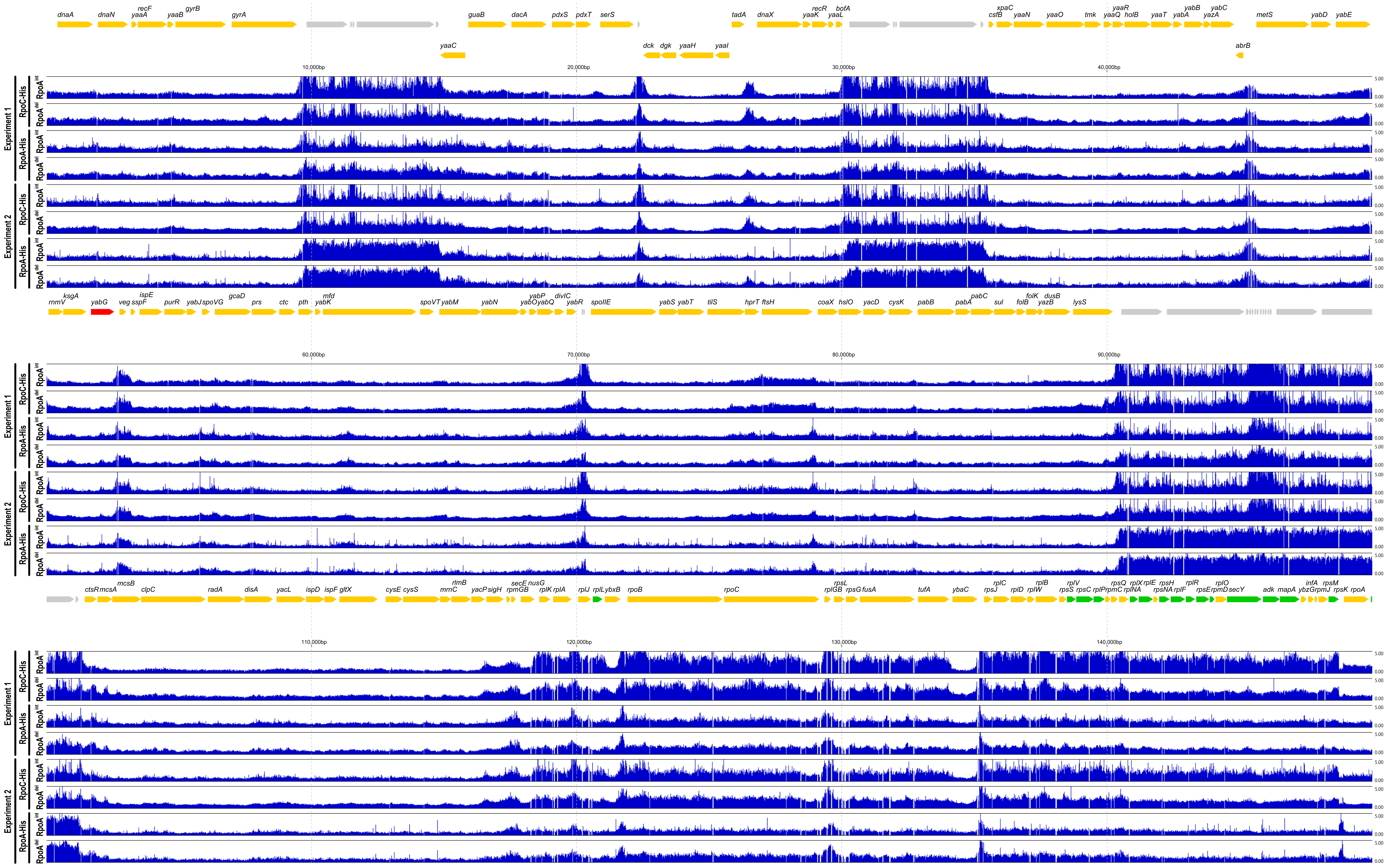

dnaA dnaN recF yaaA yaaB gyrB gyrA guaB dacA pdxS pdxT serS tadA dnaX recR yaaK bofA yaaL xpaC csfB yaaN yaaO tmk yaaR yaaQ holB yaaT yabB yabA yabC yazA metS yabD yabE
yaaC dck dgk yaaH yaaI abrB
10,000bp
20,000bp
30,000bp
40,000bp
Experiment 1
Experiment 2
RpoC-His
RpoA-His
RpoA^int
RpoA^del
5.00
0.00
rrnV ksgA yabG veg sspF ispE purR yabJ spoVG gcaD prs ctc pth mfd yabK spoVT yabM yabN yabP yabO yabQ divIC yabR spoIIE yabS yabT tilS hprT ftsH coaX hslO yacD cysK pabB pabA pabC sul folK folB dusB yazB lysS
60,000bp
70,000bp
80,000bp
90,000bp
ctsR mcsA mcsB clpC radA disA yacL ispD ispF gltX cysE cysS mrnC rlmB yacP sigH secE rpmGB nusG rplK rplA rplJ rplL ybxB rpoB rpoC rpsL rplGB rpsG fusA tufA ybaC rpsJ rplC rplD rplB rplW rplV rpsS rpsC rpsQ rplP rpmC rplX rplNA rplE rpsH rpsNA rplF rplR rpsE rplO rpmD secY adk mapA infA ybzG rpmJ rpsM rpsK rpoA
110,000bp
120,000bp
130,000bp
140,000bp

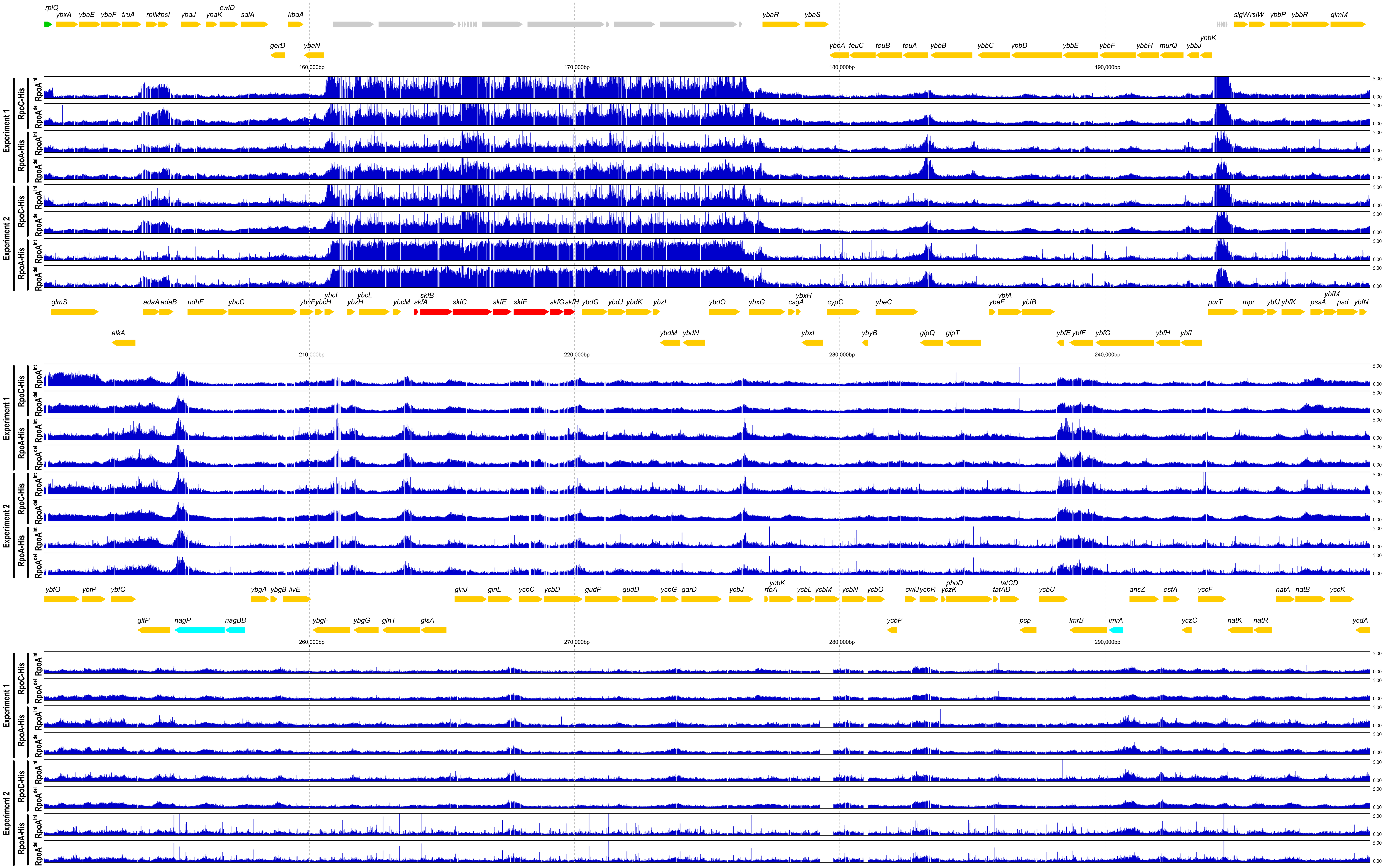

rplQ ybxA ybaE ybaF truA rplM rpsI ybaJ ybaK cwlD salA kbaA
gerD ybaN
ybaR ybaS
sigW rsiW ybbP ybbR glmM
ybbA feuC feuB feuA ybbB ybbC ybbD ybbE ybbF ybbH murQ ybbJ ybbK
160,000bp
170,000bp
180,000bp
190,000bp
Experiment 1
Experiment 2
RpoC-His
RpoA-His
RpoA^int
RpoA^del
5.00
0.00
glmS adaA adaB ndhF ybcC ybcF ybcH ybcI ybzH ybcL ybcM skfA skfB skfC skfE skfF skfG skfH ybdG ybdJ ybdK ybzI ybdO ybxG csgA ybxH cypC ybeC ybeF ybfA ybfB purT mpr ybfJ ybfK pssA psd ybfM ybfN
alkA ybdM ybdN ybxI ybyB glpQ glpT ybfE ybfF ybfG ybfH ybfI
210,000bp
220,000bp
230,000bp
240,000bp
ybfO ybfP ybfQ ybgA ybgB ilvE glnJ glnL ycbC ycbD gudP gudD ycbG garD ycbJ rtpA ycbK ycbL ycbM ycbN ycbO cwlJ ycbR yczK phoD tatAD tatCD ycbU ansZ estA yccF natA natB yccK
gltP nagP nagBB ybgF ybgG glnT glsA ycbP pcp lmrB lmrA yczC natK natR ycdA
260,000bp
270,000bp
280,000bp
290,000bp

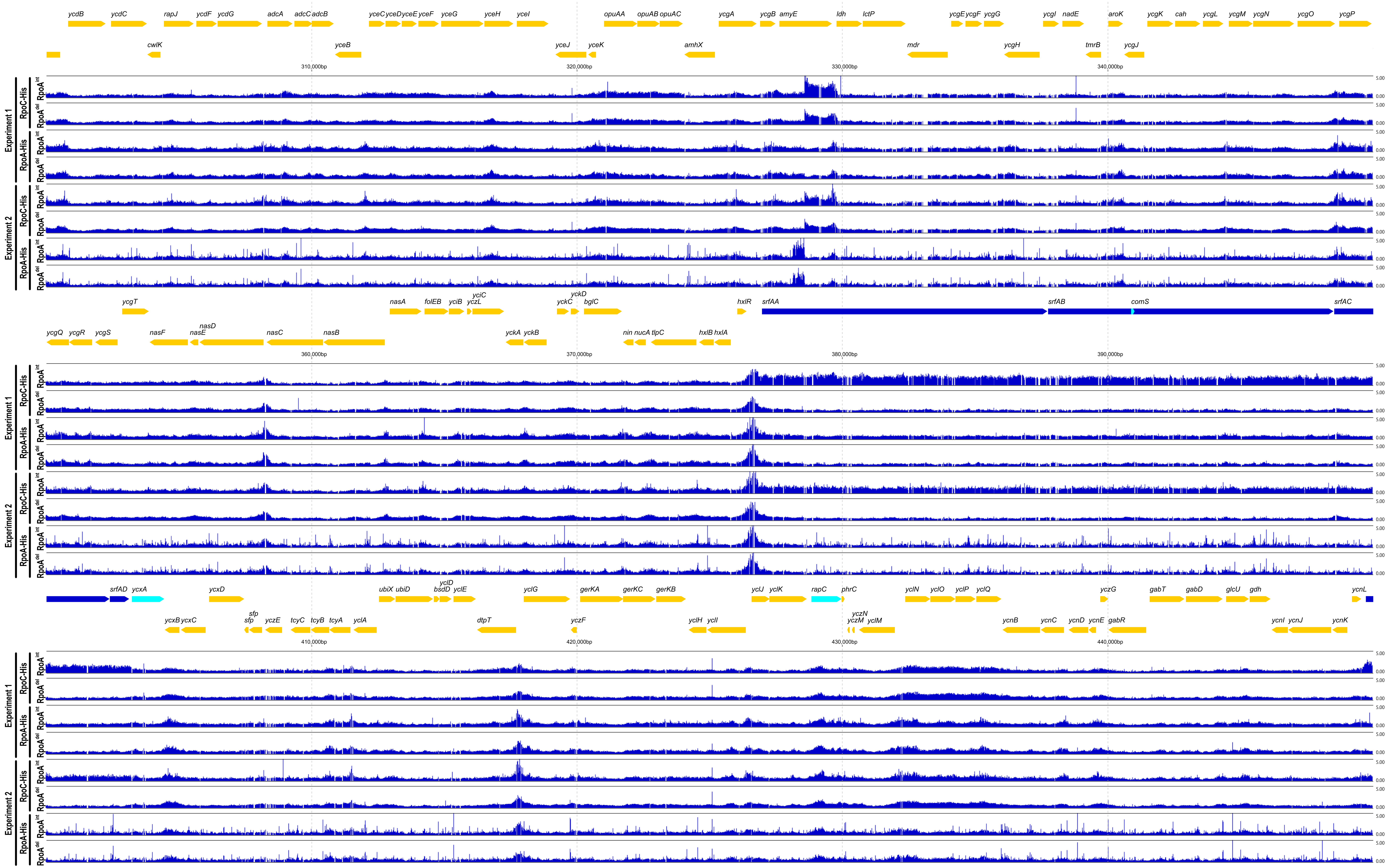
ycdB ycdC rapJ ycdF ycdG adcA adcC adcB yceC yceD yceE yceF yceG yceH yceI opuAA opuAB opuAC ycgA ycgB amyE ldh lctP ycgE ycgF ycgG ycgI nadE aroK ycgK cah ycgL ycgM ycgN ycgO ycgP
cwlK yceB yceJ yceK amhX mdr ycgH tmrB ycgJ
310,000bp 320,000bp 330,000bp 340,000bp
ycgT nasA folEB yciB yciC yczL yckC yckD bglC hxlR srfAA srfAB comS srfAC
ycgQ ycgR ycgS nasF nasE nasD nasC nasB yckA yckB nin nucA tlpC hxlB hxlA
360,000bp 370,000bp 380,000bp 390,000bp
srfAD ycxA ycxD ubiX ubiD yclD bsdD yclE yclG gerKA gerKC gerKB yclJ yclK rapC phrC yclN yclO yclP yclQ yczG gabT gabD glcU gdh ycnL
ycxB ycxC sfp sfp yczE tcyC tcyB tcyA yclA dtpT yczF yclH yclI yczN yczM yclM ycnB ycnC ycnD ycnE gabR ycnI ycnJ ycnK
410,000bp 420,000bp 430,000bp 440,000bp
Experiment 1
Experiment 2
RpoC-His
RpoA-His
RpoA^int
RpoA^del
5.00
0.00

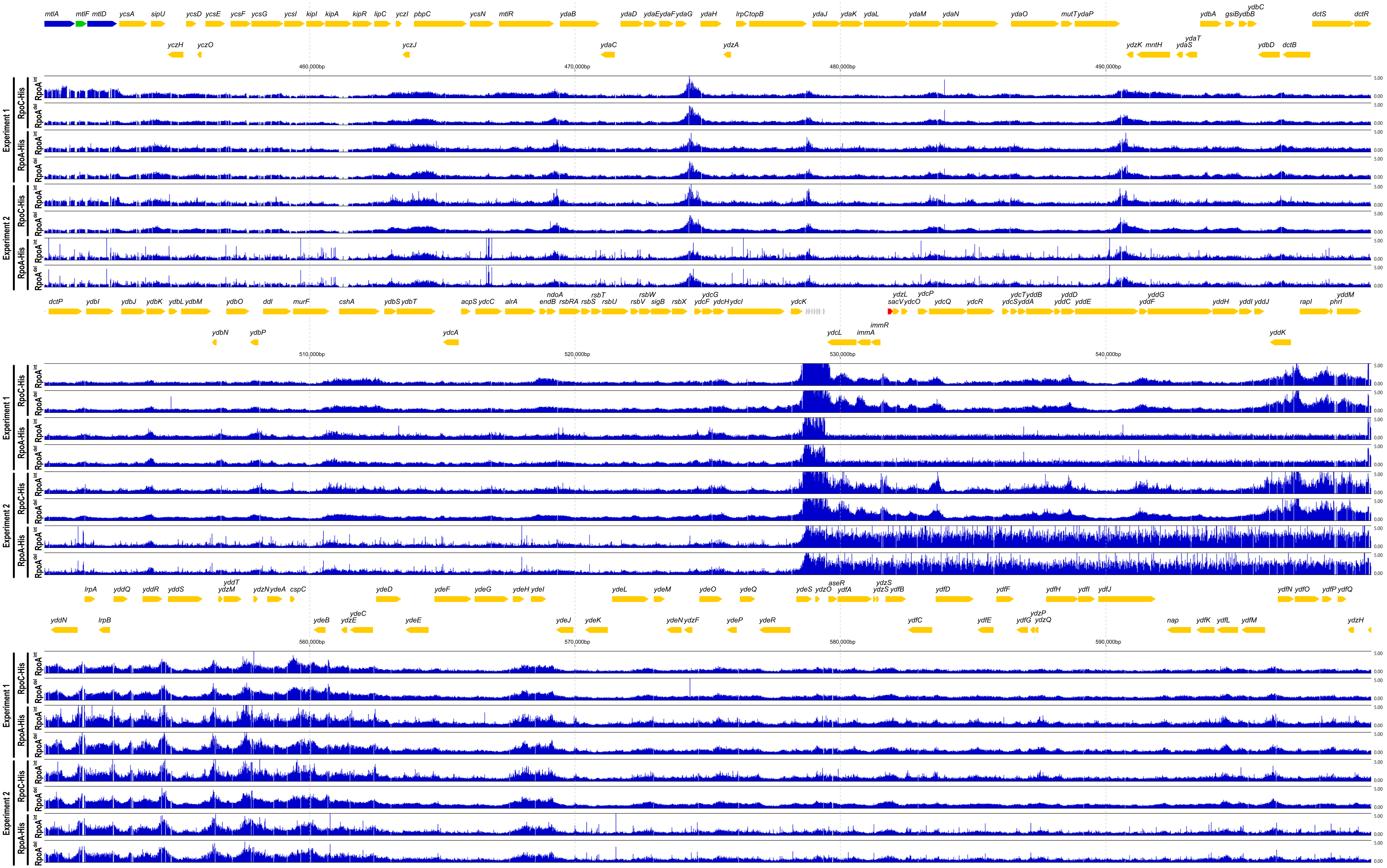
mtlA mtlF mtlD ycsA sipU ycsD ycsE ycsF ycsG ycsI kipI kipA kipR lipC yczI pbpC ycsN mtlR ydaB ydaD ydaE ydaF ydaG ydaH lrpC topB ydaJ ydaK ydaL ydaM ydaN ydaO mutT ydaP ydbA gsiB ydbB ydbC dctS dctR
yczH yczO yczJ ydaC ydzA ydzK mntH ydaT ydaS ydbD dctB
460,000bp
470,000bp
480,000bp
490,000bp
Experiment 1
Experiment 2
RpoC-His
RpoA-His
RpoA^int
RpoA^del
5.00
0.00
dctP ydbI ydbJ ydbK ydbL ydbM ydbO ddl murF cshA ydbS ydbT acpS ydcC alrA ndoA endB rsbRA rsbS rsbT rsbU rsbV rsbW sigB rsbX ydcG ydcF ydcH ydcI ydcK ydzL sacV ydcO ydcP ydcQ ydcR ydcT yddB ydcS yddA yddD yddC yddE yddG yddF yddH yddI yddJ rapI phrI yddM
ydbN ydbP ydcA ydcL immA immR yddK
510,000bp
520,000bp
530,000bp
540,000bp
lrpA yddQ yddR yddS yddT ydzM ydzN ydeA cspC ydeD ydeF ydeG ydeH ydeI ydeL ydeM ydeO ydeQ ydeS ydzO aseR ydfA ydzS ydzS ydfB ydfD ydfF ydfH ydfI ydfJ ydfN ydfO ydfP ydfQ
yddN lrpB ydeB ydeC ydzE ydeE ydeJ ydeK ydeN ydzF ydeP ydeR ydfC ydfE ydzP ydfG ydzQ nap ydfK ydfL ydfM ydzH
560,000bp
570,000bp
580,000bp
590,000bp

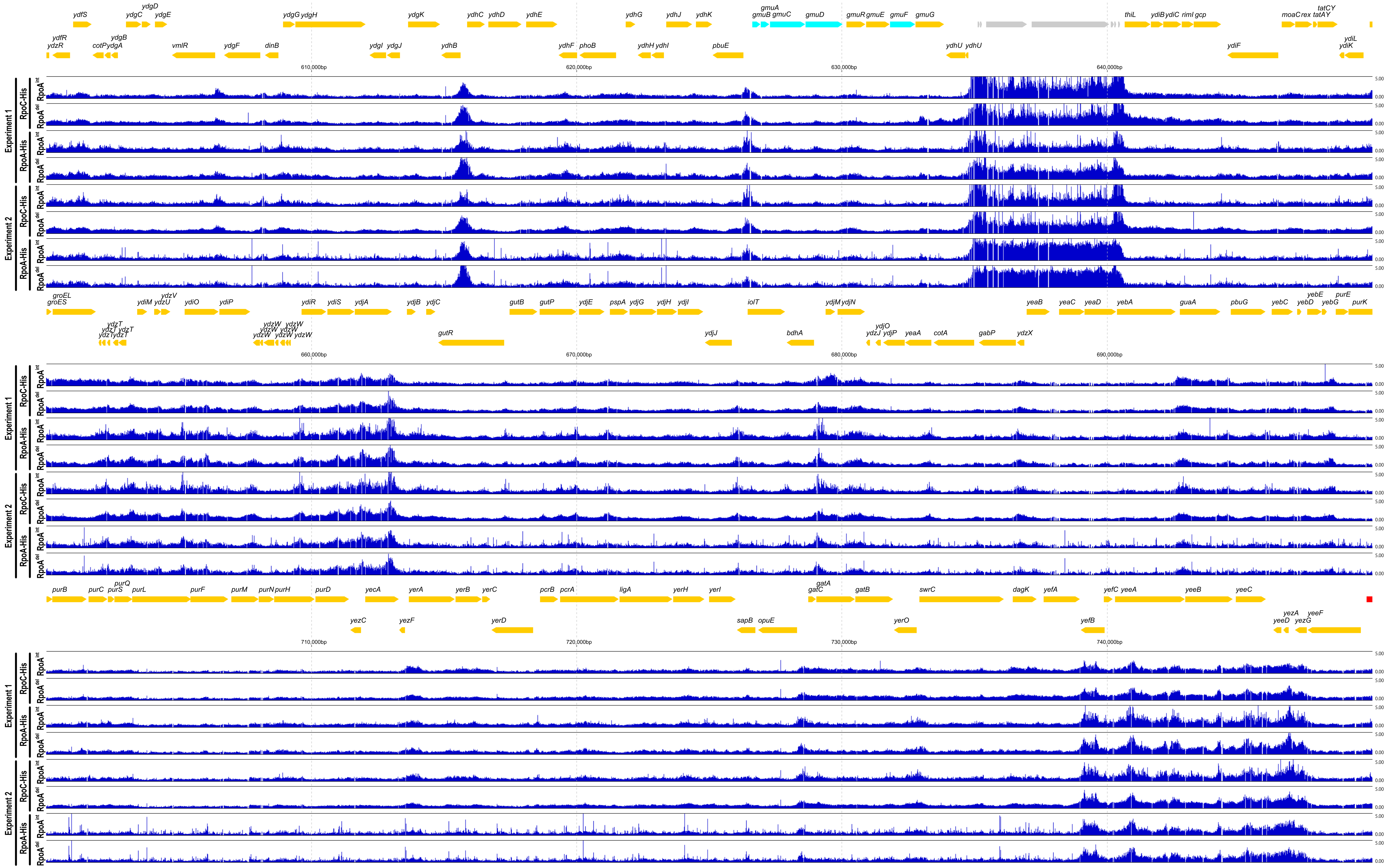
ydgD
ydfS ydgC ydgE ydgG ydgH ydgK ydhC ydhD ydhE ydhG ydhJ ydhK
gmuA
gmuB gmuC gmuD gmuR gmuE gmuF gmuG thiL ydiB ydiC rimI gcp moaC rex
tatCY
tatAY
ydfR ydgB ydiL
ydzR cotP ydgA vmlR ydgF dinB ydgI ydgJ ydhB ydhF phoB ydhH ydhI pbuE ydhU ydhU ydiF ydiK
610,000bp
620,000bp
630,000bp
640,000bp
Experiment 1
Experiment 2
RpoC-His
RpoA-His
RpoA^int
RpoA^del
5.00
0.00
groEL
groES ydiM ydzU ydzV ydiO ydiP ydiR ydiS ydjA ydjB ydjC gutB gutP ydjE pspA ydjG ydjH ydjI iolT ydjM ydjN yeaB yeaC yeaD yebA guaA pbuG yebC yebD yebE yebG purE purK
ydzT ydzT ydzT ydzT ydzT
ydzW ydzW ydzW ydzW ydzW ydzW ydzW
gutR ydjJ bdhA ydjO ydzJ ydjP yeaA cotA gabP ydzX
660,000bp
670,000bp
680,000bp
690,000bp
purQ
purB purC purS purL purF purM purN purH purD yecA yerA yerB yerC pcrB pcrA ligA yerH yerI
gatA
gatC gatB swrC dagK yefA yefC yeeA yeeB yeeC
yezC yezF yerD sapB opuE yerO yefB yezA yeeF yeeD yezG
710,000bp
720,000bp
730,000bp
740,000bp

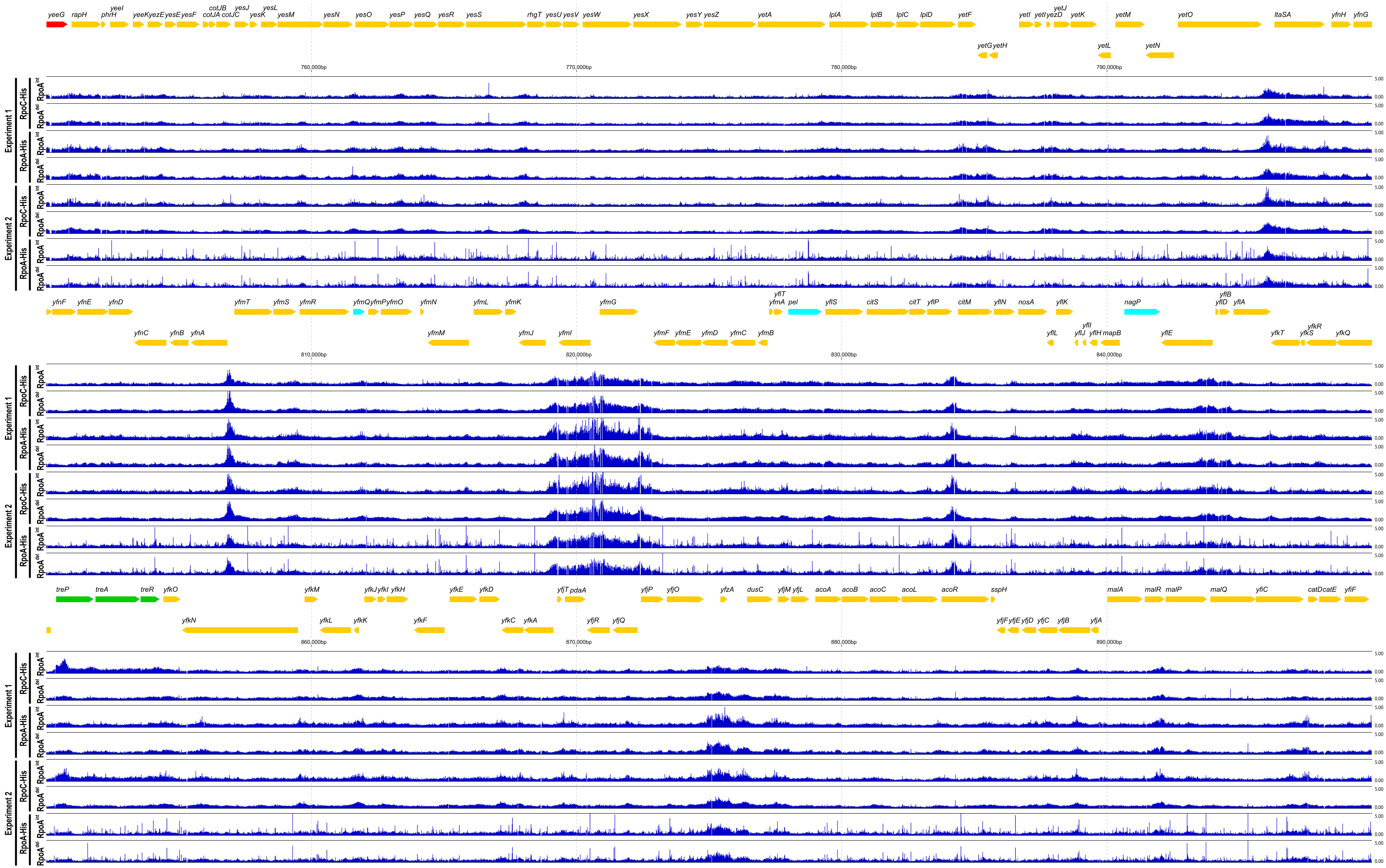
yeeG rapH phrH yeeI yeeKyezEyesEyesF cotJB cotJA cotJC yesJ yesK yesL yesM yesN yesO yesP yesQ yesR yesS rhgT yesU yesV yesW yesX yesY yesZ yetA lplA lplB lplC lplD yetF yetI yetI yezD yetJ yetK yetM yetO ltaSA yfnH yfnG
yetG yetH yetL yetN
760,000bp 770,000bp 780,000bp 790,000bp
Experiment 1
Experiment 2
RpoC-His
RpoA-His
RpoA int
RpoA del
5.00
0.00
yfnF yfnE yfnD yfmT yfmS yfmR yfmQyfmPyfmO yfmN yfmL yfmK yfmG yflT yfmA pel yflS citS citT yflP citM yflN nosA yflK nagP yflB yflD yflA
yfnC yfnB yfnA yfmM yfmJ yfmI yfmF yfmE yfmD yfmC yfmB yflL yflI yflJ yflH mapB yflE yfkT yfkR yfkS yfkQ
810,000bp 820,000bp 830,000bp 840,000bp
treP treA treR yfkO yfkM yfkJ yfkI yfkH yfkE yfkD yfjT pdaA yfjP yfjO yfzA dusC yfjM yfjL acoA acoB acoC acoL acoR sspH malA malR malP malQ yfiC catDcatE yfiF
yfkN yfkL yfkK yfkF yfkC yfkA yfjR yfjQ yfjF yfjE yfjD yfjC yfjB yfjA
860,000bp 870,000bp 880,000bp 890,000bp

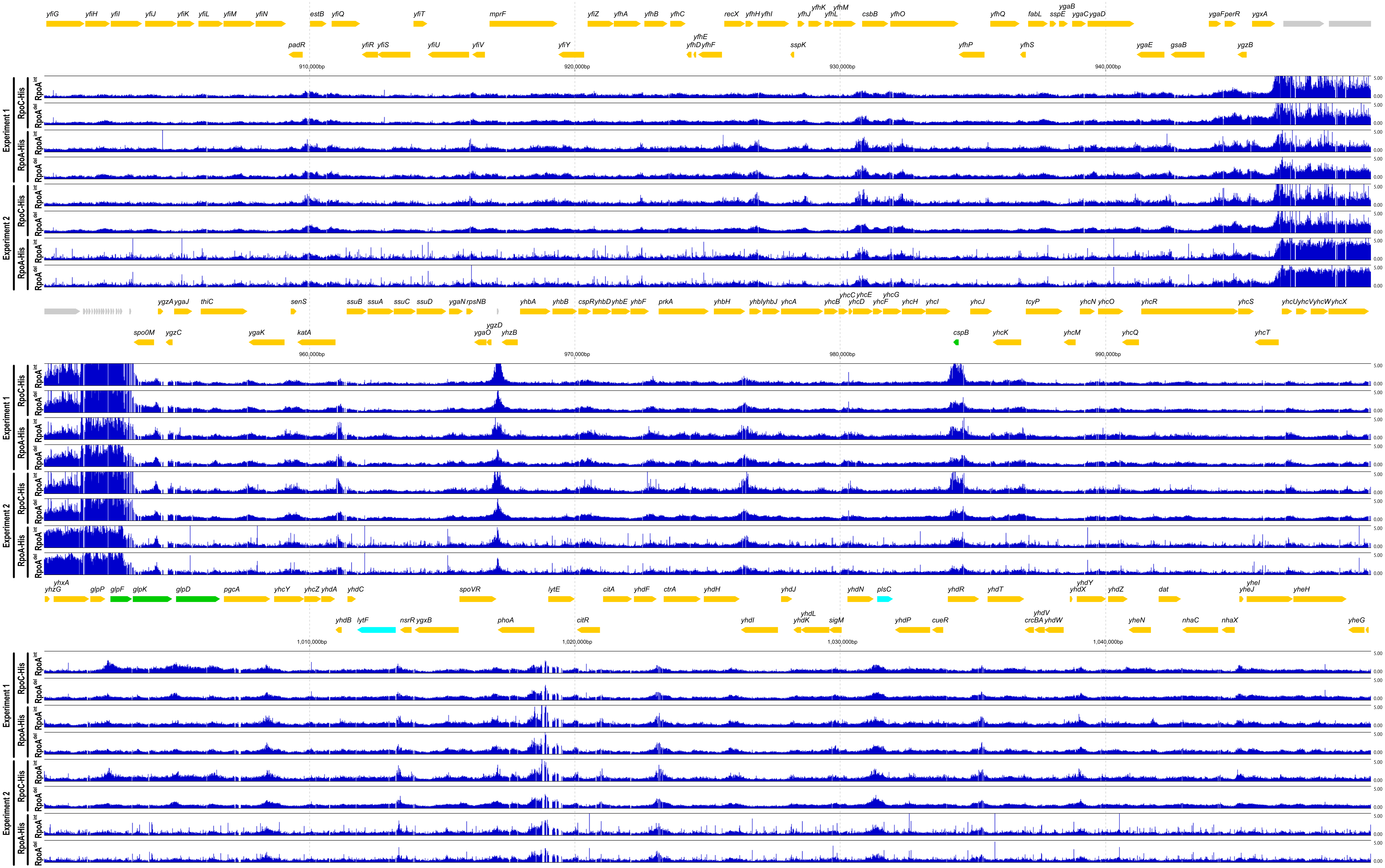

910,000bp
920,000bp
930,000bp
940,000bp
960,000bp
970,000bp
980,000bp
990,000bp
1,010,000bp
1,020,000bp
1,030,000bp
1,040,000bp
Experiment 1
Experiment 2
RpoC-His
RpoA-His
RpoA^int
RpoA^del
5.00
0.00

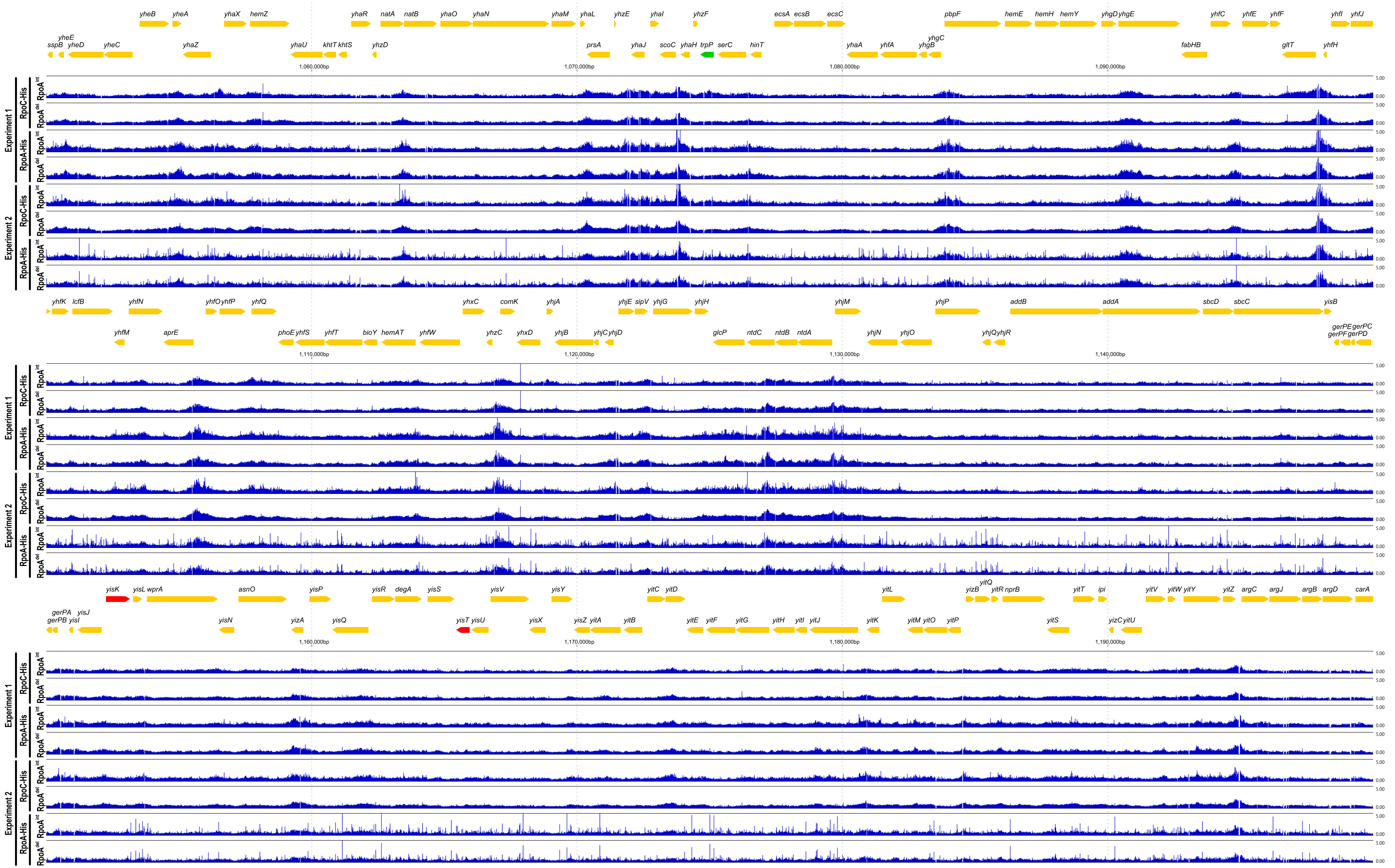
yheB yheA yhaX hemZ yhaR natA natB yhaO yhaN yhaM yhaL yhzE yhaI yhzF ecsA ecsB ecsC pbpF hemE hemH hemY yhgD yhgE yhfC yhfE yhfF yhfI yhfJ
yheE sspB yheD yheC yhaZ yhaU khtT khtS yhzD prsA yhaJ scoC yhaH trpP serC hinT yhaA yhfA yhgC yhgB fabHB gltT yhfH
1,060,000bp 1,070,000bp 1,080,000bp 1,090,000bp
yhfK lcfB yhfN yhfO yhfP yhfQ yhxC comK yhjA yhjE sipV yhjG yhjH yhjM yhjP addB addA sbcD sbcC yisB
yhfM aprE phoE yhfS yhfT bioY hemAT yhfW yhzC yhxD yhjB yhjC yhjD glcP ntdC ntdB ntdA yhjN yhjO yhjQ yhjR gerPE gerPC gerPF gerPD
1,110,000bp 1,120,000bp 1,130,000bp 1,140,000bp
yisK yisL wprA asnO yisP yisR degA yisS yisV yisY yitC yitD yitL yitQ yizB yitR nprB yitT ipi yitV yitW yitY yitZ argC argJ argB argD carA
gerPA yisJ gerPB yisI yisN yizA yisQ yisT yisU yisX yisZ yitA yitB yitE yitF yitG yitH yitI yitJ yitK yitM yitO yitP yitS yizC yitU
1,160,000bp 1,170,000bp 1,180,000bp 1,190,000bp
Experiment 1
Experiment 2
RpoC-His
RpoA-His
RpoA^int
RpoA^del
5.00
0.00

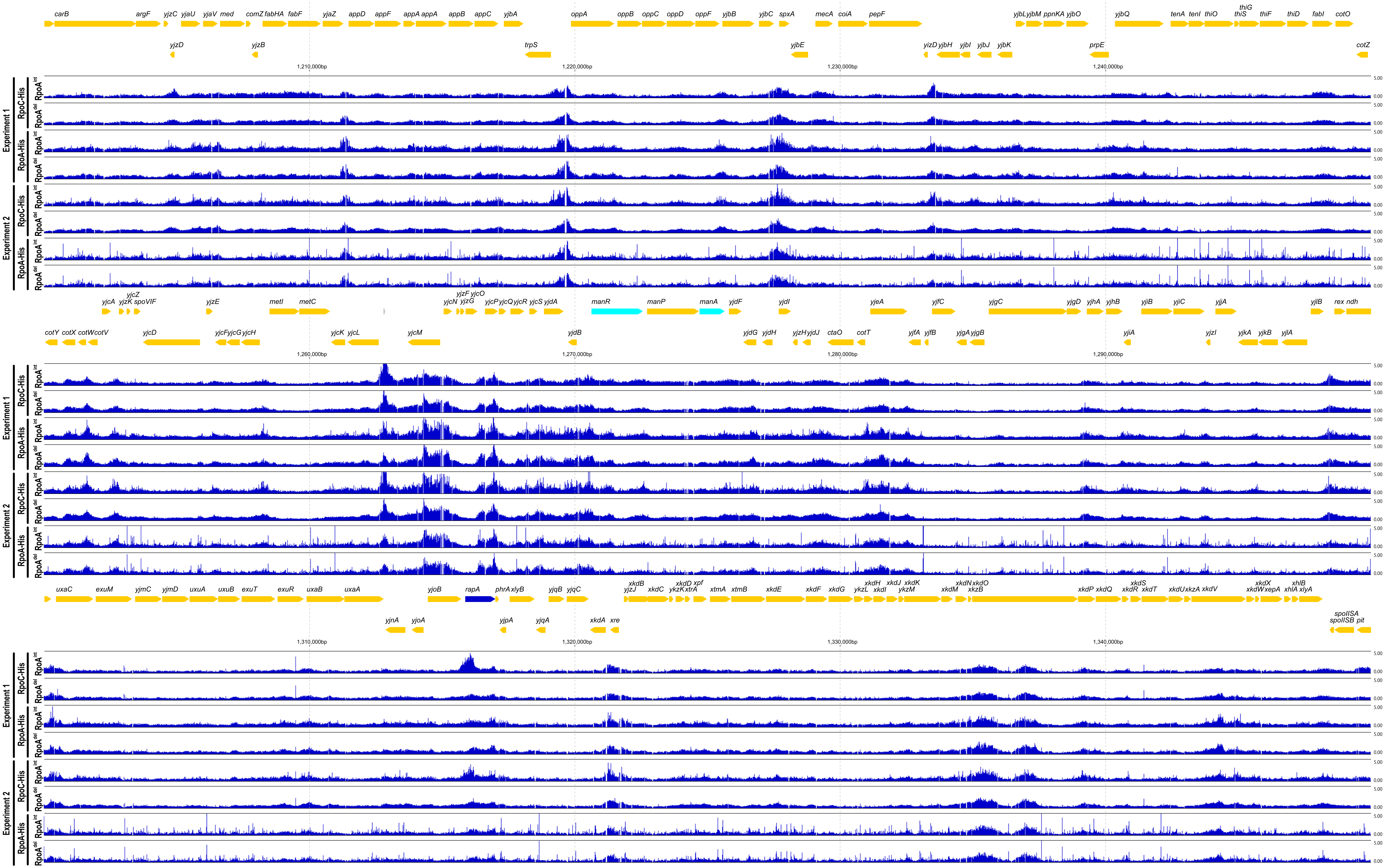
carB argF yjzC yjaU yjaV med comZ fabHA fabF yjaZ appD appF appA appA appB appC yjbA oppA oppB oppC oppD oppF yjbB yjbC spxA mecA coiA pepF yjbL yjbM ppnKA yjbO yjbQ tenA tenI thiO thiG thiS thiF thiD fabI cotO
yjzD yjzB trpS yjbE yizD yjbH yjbI yjbJ yjbK prpE cotZ
1,210,000bp
1,220,000bp
1,230,000bp
1,240,000bp
yjcZ yjcA yjzK spoVIF yjzE metI metC yjzF yjcO yjcN yjzG yjcP yjcQ yjcR yjcS yjdA manR manP manA yjdF yjdI yjeA yjfC yjgC yjgD yjhA yjhB yjiB yjiC yjjA yjlB rex ndh
cotY cotX cotW cotV yjcD yjcF yjcG yjcH yjcK yjcL yjcM yjdB yjdG yjdH yjzH yjdJ ctaO cotT yjfA yjfB yjgA yjgB yjiA yjzI yjkA yjkB yjlA
1,260,000bp
1,270,000bp
1,280,000bp
1,290,000bp
uxaC exuM yjmC yjmD uxuA uxuB exuT exuR uxaB uxaA yjoB rapA phrA xlyB yjqB yjqC xkdB yjzJ xkdC xkdD xpf ykzK xtrA xtmA xtmB xkdE xkdF xkdG xkdH ykzL xkdI xkdJ xkdK ykzM xkdN xkdO xkdM xkzB xkdP xkdQ xkdS xkdR xkdT xkdU xkzA xkdV xkdX xkdW xepA xhlB xhlA xlyA
yjnA yjoA yjpA yjqA xkdA xre spoIISA spoIISB pit
1,310,000bp
1,320,000bp
1,330,000bp
1,340,000bp
Experiment 1
Experiment 2
RpoC-His
RpoA-His
RpoA[int]
RpoA[del]
5.00
0.00

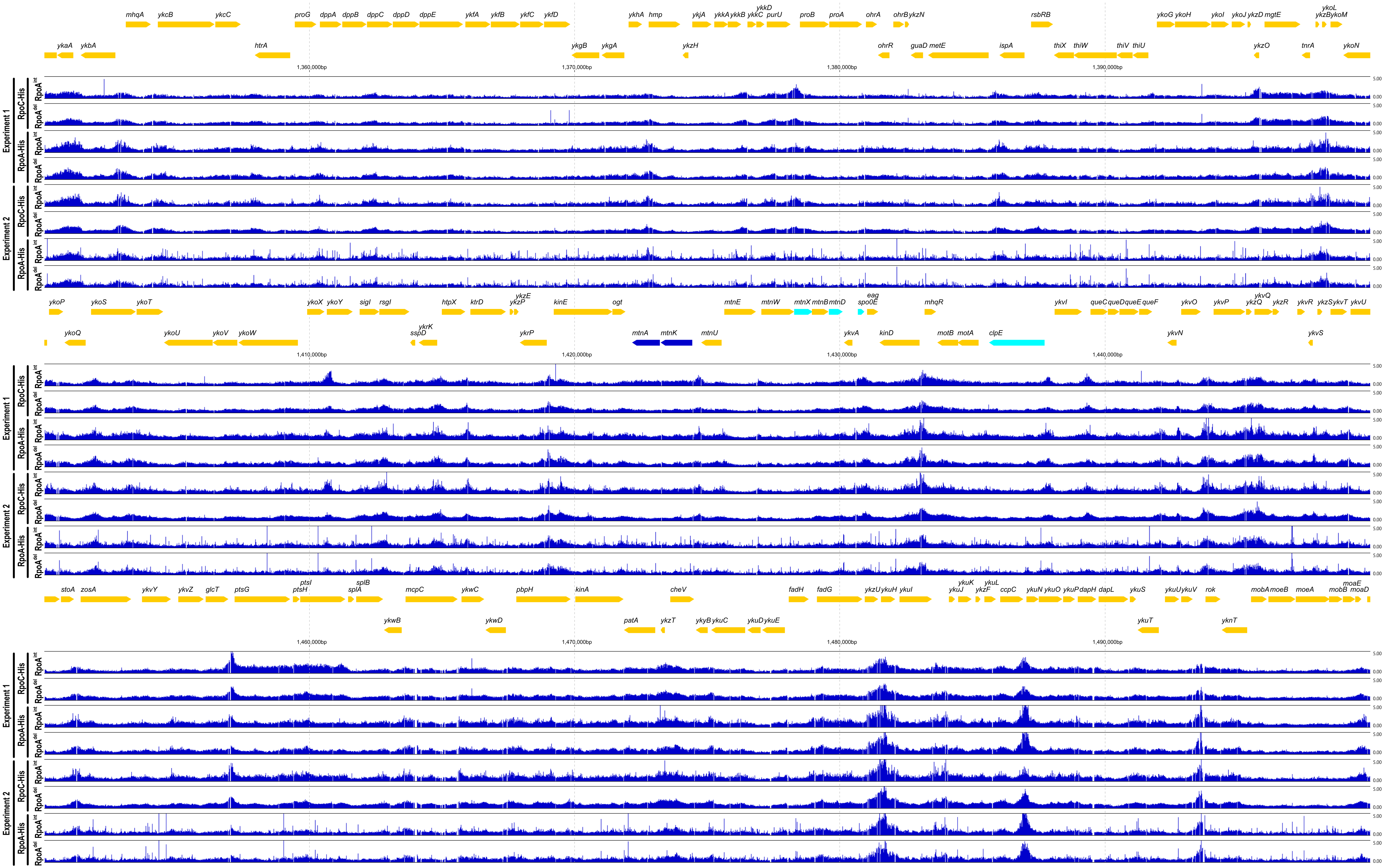

mhqA ykcB ykcC proG dppA dppB dppC dppD dppE ykfA ykfB ykfC ykfD ykhA hmp ykjA ykkA ykkB ykkD ykkC purU proB proA ohrA ohrB ykzN rsbRB ykoG ykoH ykoI ykoJ ykzD mgtE ykoL ykzB ykoM
ykaA ykbA htrA ykgB ykgA ykzH ohrR guaD metE ispA thiX thiW thiV thiU ykzO tnrA ykoN
1,360,000bp
1,370,000bp
1,380,000bp
1,390,000bp
Experiment 1
Experiment 2
RpoC-His
RpoA-His
RpoA int
RpoA del
5.00
0.00
ykoP ykoS ykoT ykoX ykoY sigI rsgI htpX ktrD ykzE ykzP kinE ogt mtnE mtnW mtnX mtnB mtnD eag spo0E mhqR ykvI queC queD queE queF ykvO ykvP ykvQ ykzQ ykzR ykvR ykzS ykvT ykvU
ykoQ ykoU ykoV ykoW ykrK sspD ykrP mtnA mtnK mtnU ykvA kinD motB motA clpE ykvN ykvS
1,410,000bp
1,420,000bp
1,430,000bp
1,440,000bp
stoA zosA ykvY ykvZ glcT ptsG ptsI ptsH splB splA mcpC ykwC pbpH kinA cheV fadH fadG ykzU ykuH ykuI ykuK ykuJ ykuL ykzF ccpC ykuN ykuO ykuP dapH dapL ykuS ykuU ykuV rok mobA moeB moeA moaE mobB moaD
ykwB ykwD patA ykzT ykyB ykuC ykuD ykuE ykuT yknT
1,460,000bp
1,470,000bp
1,480,000bp
1,490,000bp

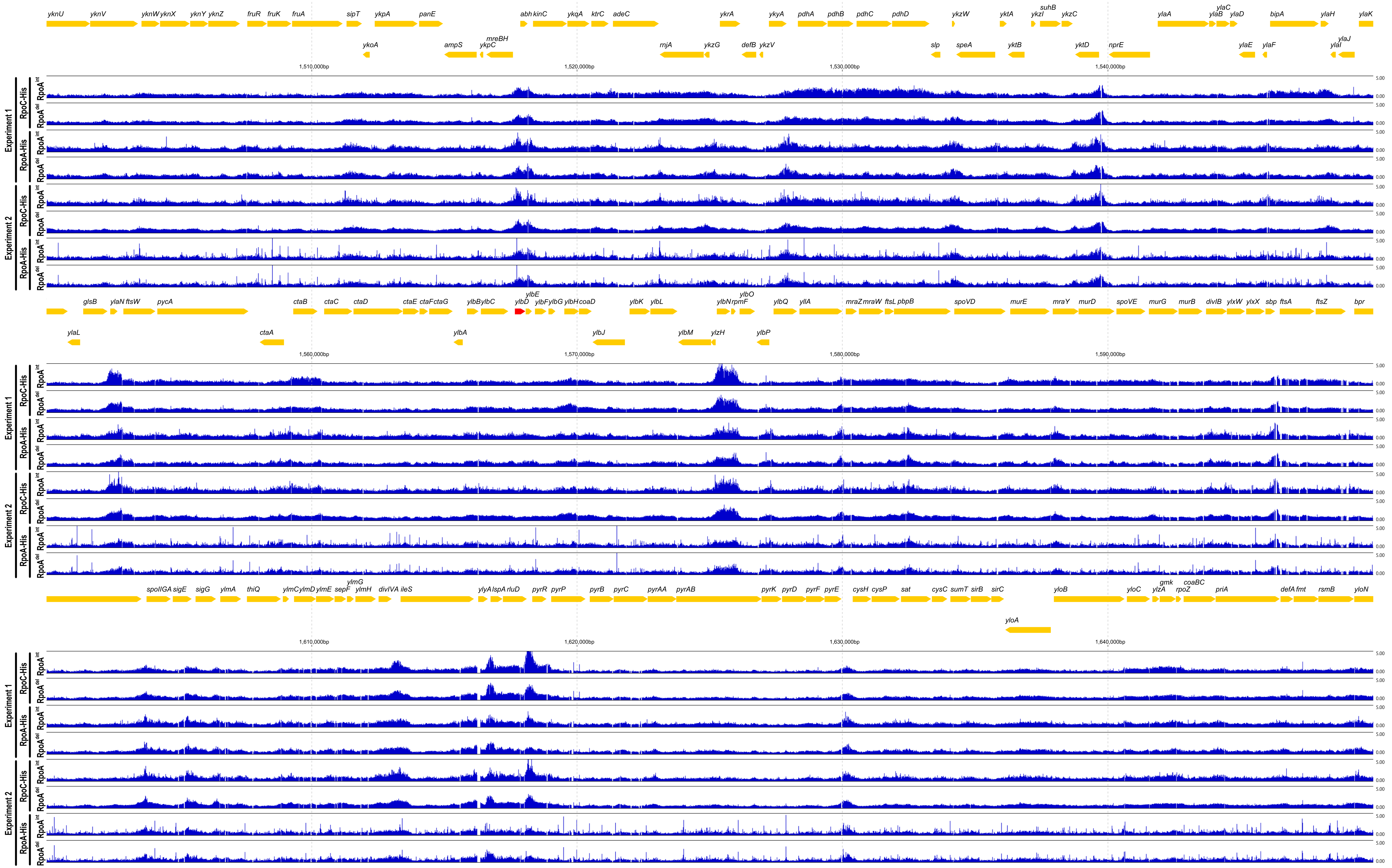

yknU yknV yknW yknX yknY yknZ fruR fruK fruA sipT ykpA panE abh kinC ykqA ktrC adeC ykrA ykyA pdhA pdhB pdhC pdhD ykzW yktA suhB ykzI ykzC ylaA ylaC ylaB ylaD bipA ylaH ylaK
ykoA ampS mreBH ykpC rnjA ykzG defB ykzV slp speA yktB yktD nprE ylaE ylaF ylaJ ylaI
1,510,000bp 1,520,000bp 1,530,000bp 1,540,000bp
glsB ylaN ftsW pycA ctaB ctaC ctaD ctaE ctaF ctaG ylbB ylbC ylbE ylbD ylbF ylbG ylbH coaD ylbK ylbL ylbO ylbN rpmF ylbQ yllA mraZ mraW ftsL pbpB spoVD murE mraY murD spoVE murG murB divIB ylxW ylxX sbp ftsA ftsZ bpr
ylaL ctaA ylbA ylbJ ylbM ylzH ylbP
1,560,000bp 1,570,000bp 1,580,000bp 1,590,000bp
spoIIGA sigE sigG ylmA thiQ ylmC ylmD ylmE sepF ylmG ylmH divIVA ileS ylyA lspA rluD pyrR pyrP pyrB pyrC pyrAA pyrAB pyrK pyrD pyrF pyrE cysH cysP sat cysC sumT sirB sirC yloB yloC gmk ylzA coaBC rpoZ priA defA fmt rsmB yloN
yloA
1,610,000bp 1,620,000bp 1,630,000bp 1,640,000bp
Experiment 1
Experiment 2
RpoC-His
RpoA-His
RpoA^int
RpoA^del
5.00
0.00

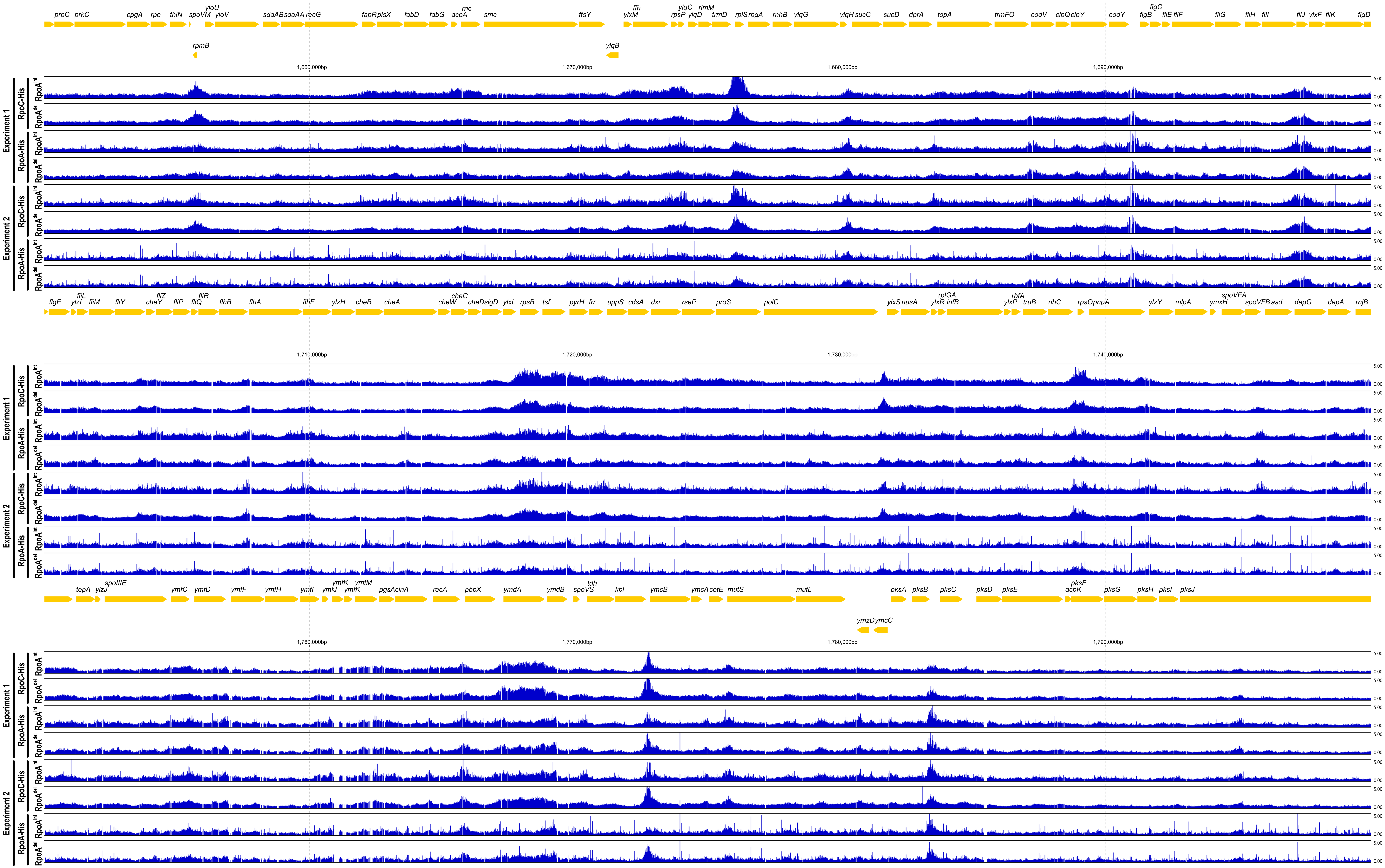

prpC prkC cpgA rpe thiN yloU spoVM yloV sdaAB sdaAA recG fapR plsX fabD fabG rnc acpA smc ftsY ffh ylxM ylqC rimM rpsP ylqD trmD rplS rbgA rnhB ylqG ylqH sucC sucD dprA topA trmFO codV clpQ clpY codY flgC flgB fliE fliF fliG fliH fliI fliJ ylxF fliK flgD
rpmB ylqB
1,660,000bp 1,670,000bp 1,680,000bp 1,690,000bp
Experiment 1
Experiment 2
RpoC-His
RpoA-His
RpoA[int]
RpoA[del]
5.00
0.00
fliL ylzI fliM fliY fliZ cheY fliP fliR fliQ flhB flhA flhF ylxH cheB cheA cheC cheW cheD sigD ylxL rpsB tsf pyrH frr uppS cdsA dxr rseP proS polC ylxS nusA rplGA ylxR infB rbfA ylxP truB ribC rpsO pnpA ylxY mlpA spoVFA ymxH spoVFB asd dapG dapA rnjB flgE
1,710,000bp 1,720,000bp 1,730,000bp 1,740,000bp
tepA ylzJ spoIIIE ymfC ymfD ymfF ymfH ymfI ymfK ymfJ ymfK ymfM pgsA cinA recA pbpX ymdA ymdB tdh spoVS kbl ymcB ymcA cotE mutS mutL pksA pksB pksC pksD pksE pksF acpK pksG pksH pksI pksJ
ymzD ymcC
1,760,000bp 1,770,000bp 1,780,000bp 1,790,000bp

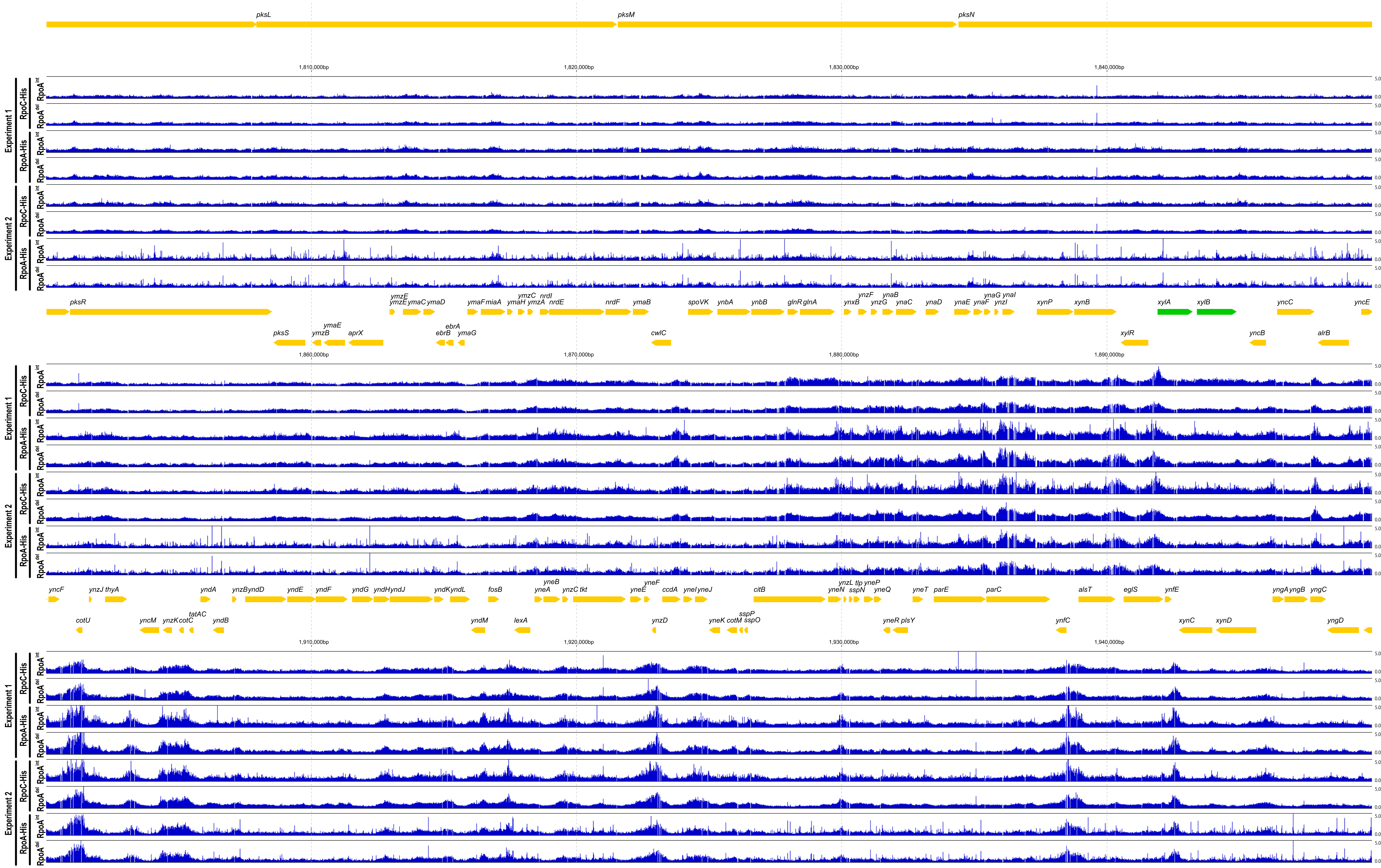
pksL
pksM
pksN
1,810,000bp
1,820,000bp
1,830,000bp
1,840,000bp
Experiment 1
Experiment 2
RpoC-His
RpoA-His
RpoA int
RpoA del
5.00
0.00
pksR
ymzE
ymzE ymaC ymaD
ymaF miaA
ymzC nrdI
ymaH ymzA nrdE
nrdF
ymaB
spoVK
ynbA
ynbB
glnR glnA
ynzF ynaB
ynxB ynzG ynaC
ynaD
ynaG ynaI
ynaE ynaF ynzI
xynP
xynB
xylA
xylB
yncC
yncE
pksS
ymaE
ymzB
aprX
ebrA
ebrB ymaG
cwlC
xylR
yncB
alrB
1,860,000bp
1,870,000bp
1,880,000bp
1,890,000bp
yncF
ynzJ thyA
yndA
ynzB yndD
yndE
yndF
yndG
yndH yndJ
yndK yndL
fosB
yneB
yneA
ynzC tkt
yneF
yneE
ccdA
yneI yneJ
citB
ynzL tlp yneP
yneN sspN
yneQ
yneT
parE
parC
alsT
eglS
ynfE
yngA yngB
yngC
cotU
yncM
ynzK cotC
tatAC
yndB
yndM
lexA
ynzD
sspP
yneK cotM sspO
yneR plsY
ynfC
xynC
xynD
yngD
1,910,000bp
1,920,000bp
1,930,000bp
1,940,000bp

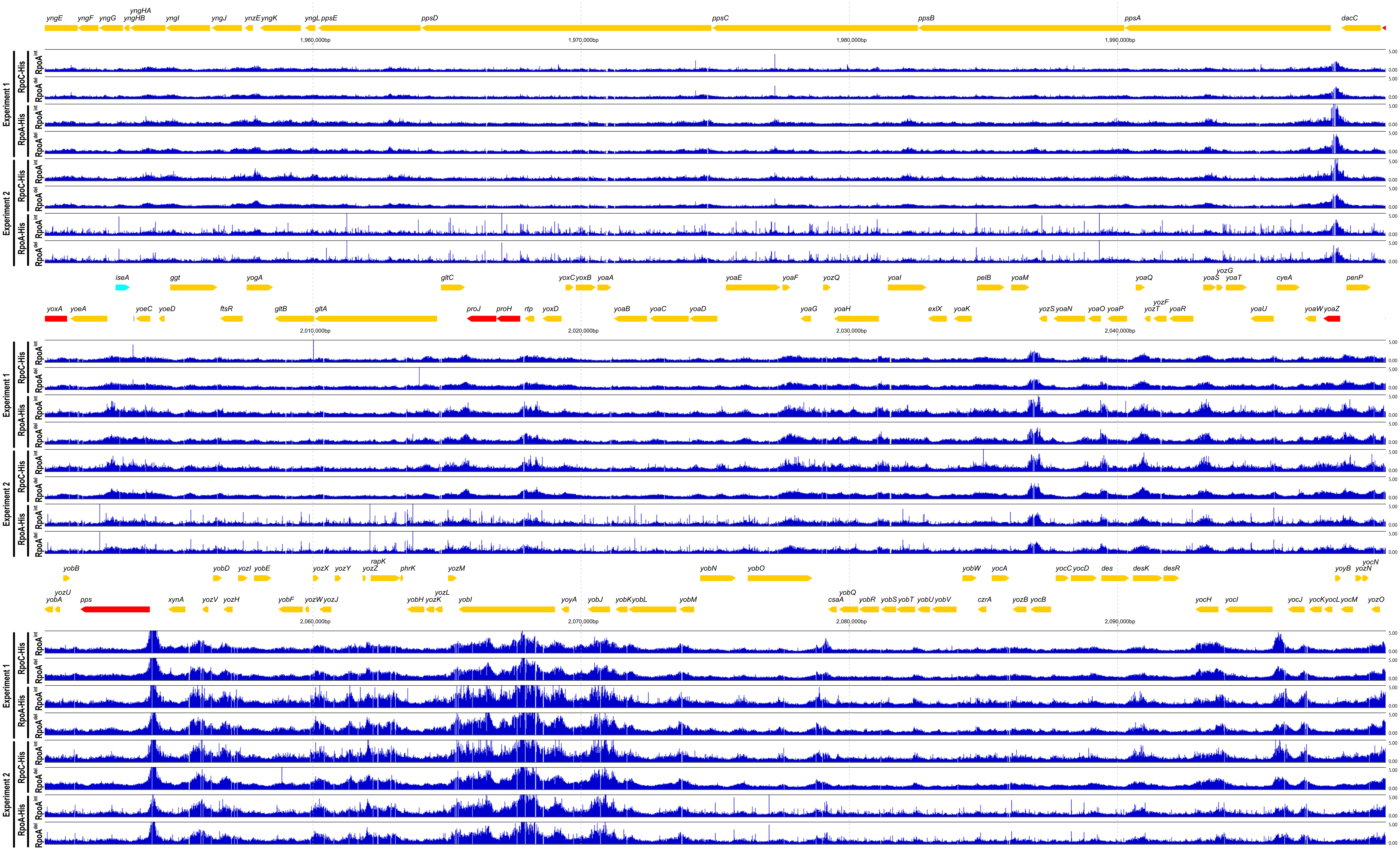
yngE yngF yngG yngHA yngHB yngI yngJ ynzE yngK yngL ppsE ppsD ppsC ppsB ppsA dacC
1,960,000bp 1,970,000bp 1,980,000bp 1,990,000bp
Experiment 1
Experiment 2
RpoC-His
RpoA-His
RpoA^int
RpoA^del
5.00
0.00
iseA ggt yogA gltC yoxC yoxB yoaA yoaE yoaF yozQ yoaI pelB yoaM yoaQ yozG yoaS yoaT cyeA penP
yoxA yoeA yoeC yoeD ftsR gltB gltA proJ proH rtp yoxD yoaB yoaC yoaD yoaG yoaH exlX yoaK yozS yoaN yoaO yoaP yozF yozT yoaR yoaU yoaW yoaZ
2,010,000bp 2,020,000bp 2,030,000bp 2,040,000bp
yobB yobD yozI yobE yozX yozY rapK yozZ phrK yozM yobN yobO yobW yocA yocC yocD des desK desR yoyB yocN yozN
yozU yobA pps xynA yozV yozH yobF yozW yozJ yozL yobH yozK yobI yoyA yobJ yobK yobL yobM yobQ csaA yobR yobS yobT yobU yobV czrA yozB yocB yocH yocI yocJ yocK yocL yocM yozO
2,060,000bp 2,070,000bp 2,080,000bp 2,090,000bp

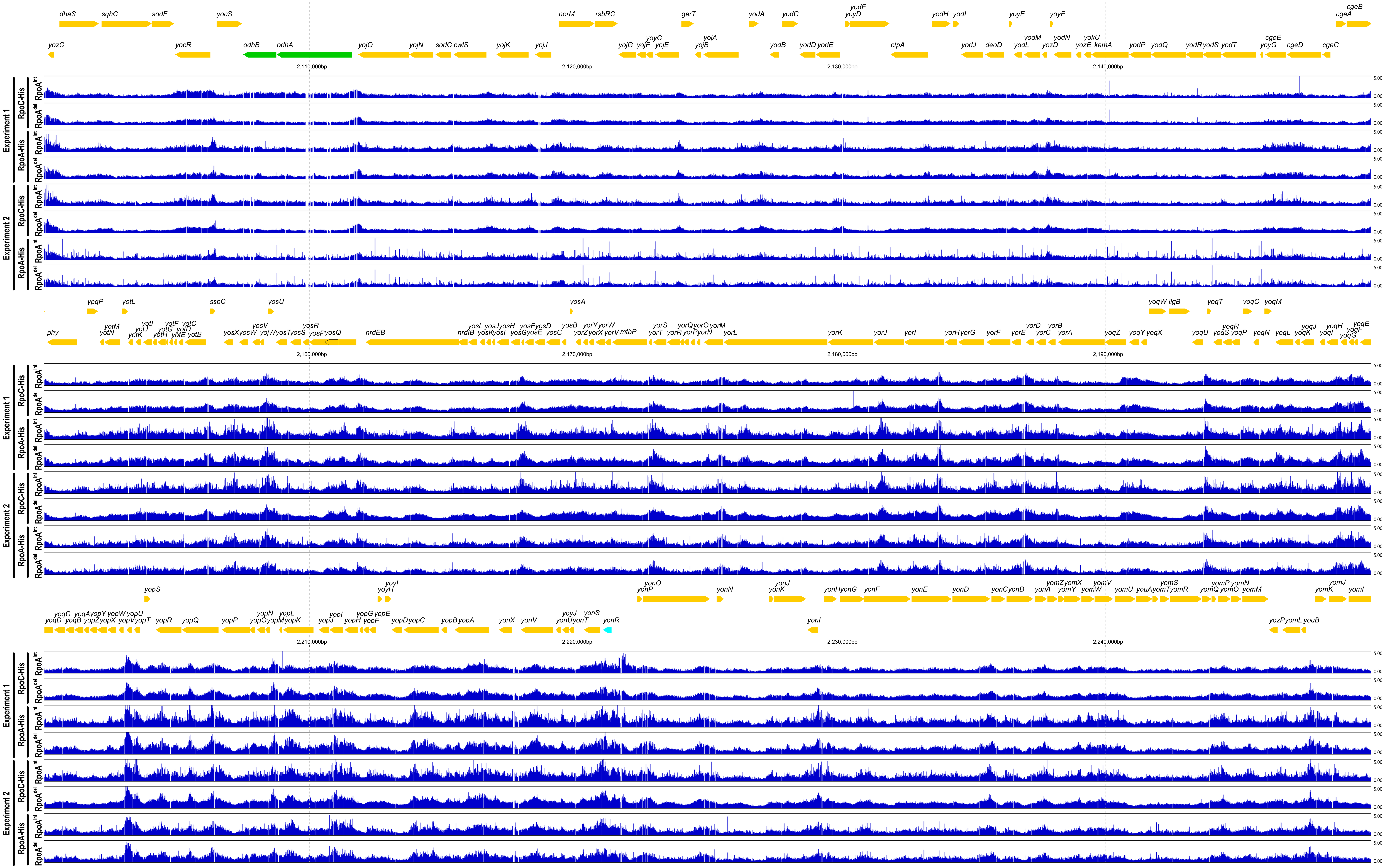
dhaS sqhC sodF yocS norM rsbRC gerT yodA yodC yodF yoyD yodH yodI yoyE yoyF cgeB cgeA
yozC yocR odhB odhA yojO yojN sodC cwlS yojK yojJ yojG yojF yoyC yojE yojA yojB yodB yodD yodE ctpA yodJ deoQ yodM yodL yodN yozD yokU yozE kamA yodP yodQ yodR yodS yodT cgeE yoyG cgeD cgeC
2,110,000bp
2,120,000bp
2,130,000bp
2,140,000bp
Experiment 1
Experiment 2
RpoC-His
RpoA-His
RpoA^int
RpoA^del
5.00
0.00
ypqP yotL sspC yosU yosA yoqW ligB yoqT yoqO yoqM
phy yotM yotN yotI yotJ yotK yotF yotG yotH yotC yotD yotE yotB yosX yosV yosW yojW yosT yosS yosR yosP yosQ nrdEB yosL nrdIB yosJ yosK yosH yosI yosF yosG yosD yosE yosC yosB yorY yorW yorZ yorX yorV mtbP yorS yorT yorQ yorR yorO yorP yorN yorM yorL yorK yorJ yorI yorH yorG yorF yorD yorE yorC yorB yorA yoqZ yoqY yoqX yoqU yoqR yoqS yoqP yoqN yoqL yoqJ yoqK yoqH yoqI yoqE yoqF yoqG
2,160,000bp
2,170,000bp
2,180,000bp
2,190,000bp
yopS yoyI yoyH yonO yonP yonN yonJ yonK yonH yonG yonF yonE yonD yonC yonB yomZ yomX yonA yomY yomV yomW yomU youA yomT yomS yomR yomP yomN yomQ yomO yomM yomJ yomK yomI
yoqC yoqD yoqB yoqA yopY yopZ yopX yopW yopV yopU yopT yopR yopQ yopP yopN yopO yopM yopL yopK yopI yopJ yopH yopG yopF yopE yopD yopC yopB yopA yonX yonV yoyJ yonU yonT yonS yonR yonI yozP yomL youB
2,210,000bp
2,220,000bp
2,230,000bp
2,240,000bp

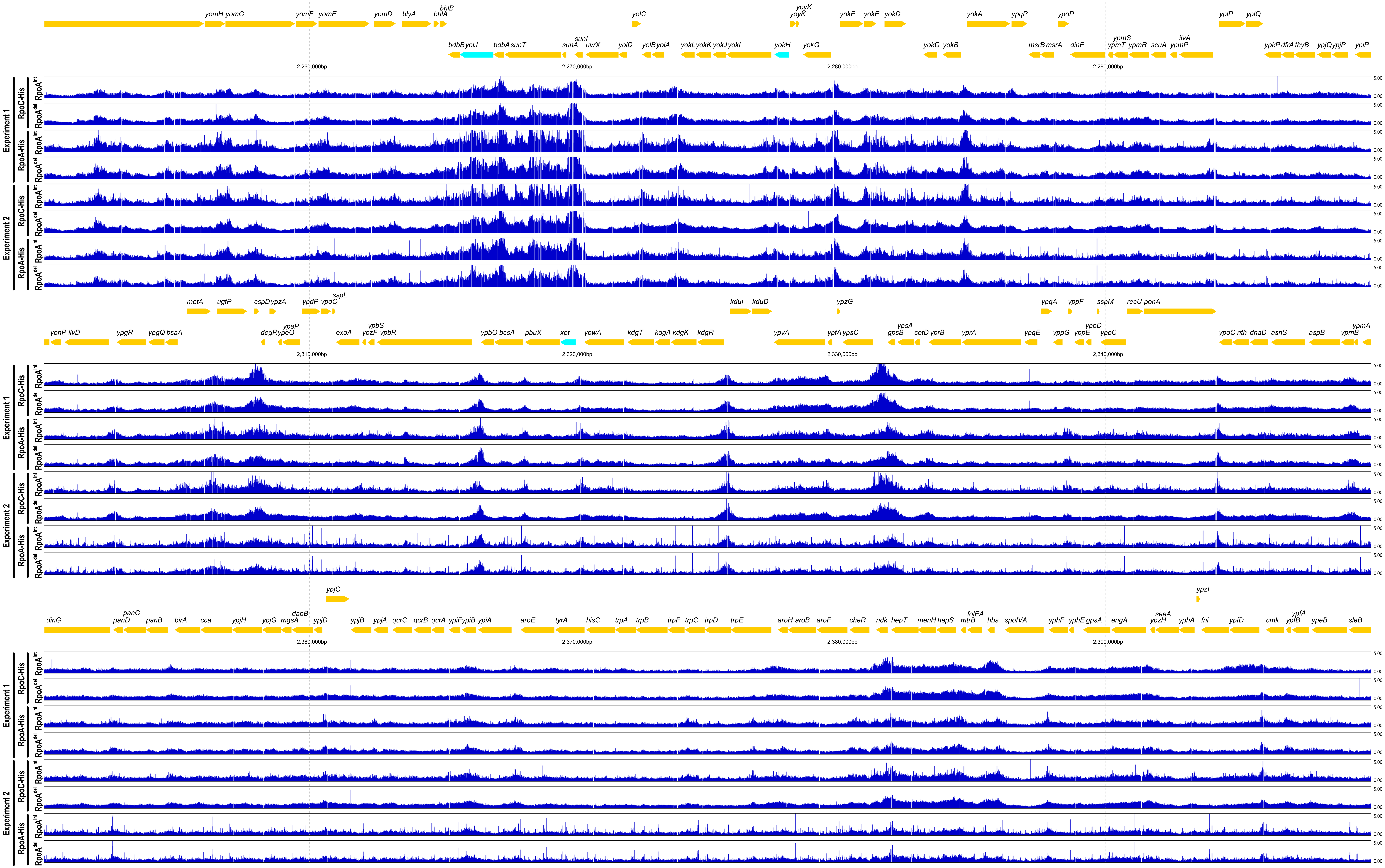

yomH yomG yomF yomE yomD blyA bhlB bhlA yolC yoyK yoyK yokF yokE yokD yokA ypqP ypoP yplP yplQ
bdbB yolJ bdbA sunT sunI sunA uvrX yolD yolB yolA yokL yokK yokJ yokI yokH yokG yokC yokB msrB msrA dinF ypmS ypmT ypmR scuA ilvA ypmP ypkP dfrA thyB ypjQ ypjP ypiP
2,260,000bp
2,270,000bp
2,280,000bp
2,290,000bp
metA ugtP cspD ypzA ypdP ypdQ sspL kduI kduD ypzG ypqA yppF sspM recU ponA
yphP ilvD ypgR ypgQ bsaA degR ypeP ypeQ exoA ypbS ypzF ypbR ypbQ bcsA pbuX xpt ypwA kdgT kdgA kdgK kdgR ypvA yptA ypsC ypsA gpsB cotD yprB yprA ypqE yppG yppD yppE yppC ypoC nth dnaD asnS aspB ypmA ypmB
2,310,000bp
2,320,000bp
2,330,000bp
2,340,000bp
ypjC ypzI
dinG panC panD panB birA cca ypjH ypjG dapB mgsA ypjD ypjB ypjA qcrC qcrB qcrA ypiF ypiB ypiA aroE tyrA hisC trpA trpB trpF trpC trpD trpE aroH aroB aroF cheR ndk hepT menH hepS folEA mtrB hbs spoIVA yphF yphE gpsA engA seaA ypzH yphA fni ypfD cmk ypfA ypfB ypeB sleB
2,360,000bp
2,370,000bp
2,380,000bp
2,390,000bp
Experiment 1
Experiment 2
RpoC-His
RpoA-His
RpoA^int
RpoA^del
5.00
0.00

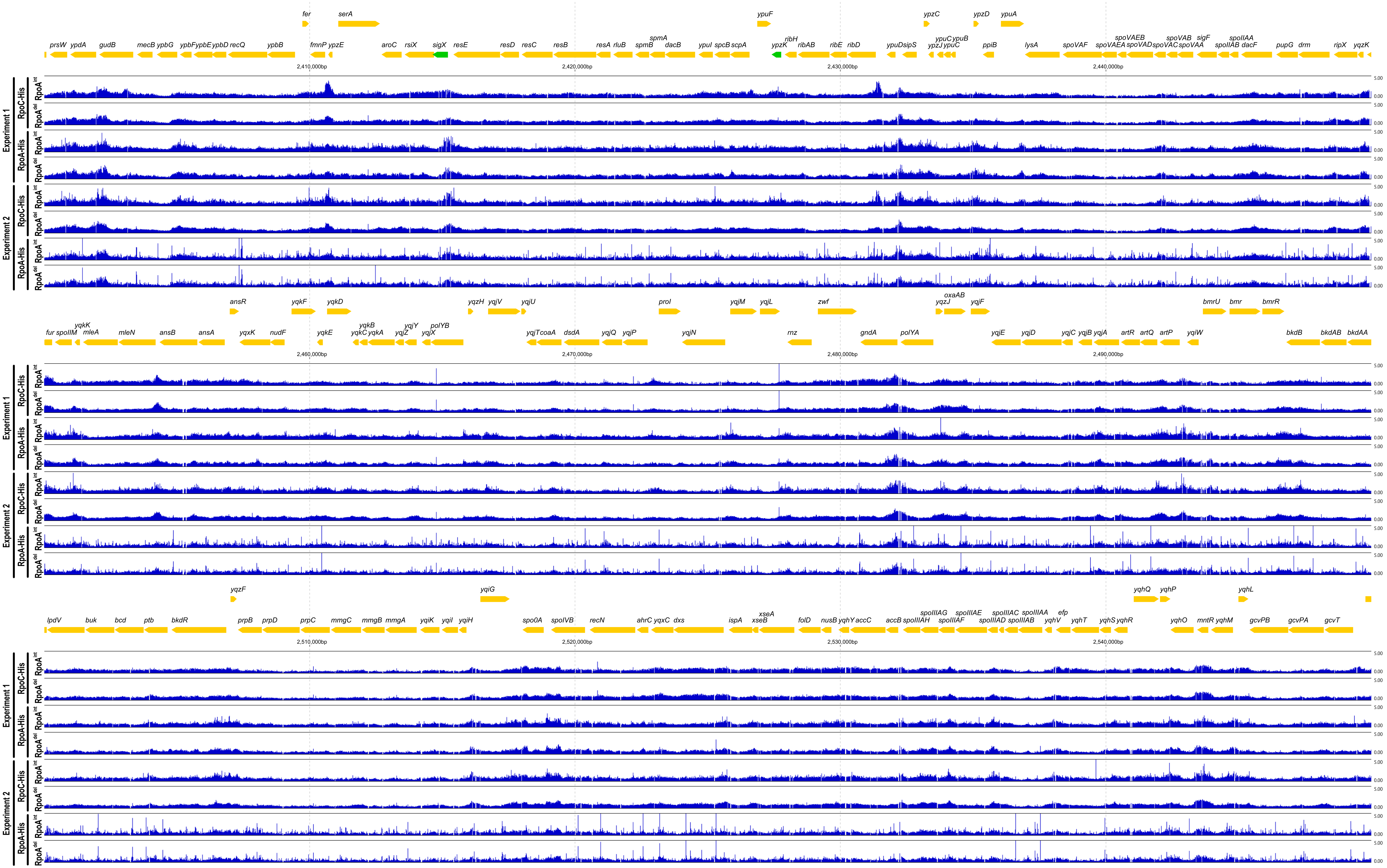

fer serA ypuF ypzC ypzD ypuA
prsW ypdA gudB mecB ypbG ypbF ypbE ypbD recQ ypbB fmnP ypzE aroC rsiX sigX resE resD resC resB resA rluB spmA spmB dacB ypuI spcB scpA ribH ypzK ribAB ribE ribD ypuD sipS ypuC ypuB ypzJ ypuC ppiB lysA spoVAF spoVAEB spoVAEA spoVAD spoVAB spoVAC spoVAA sigF spoIIAA spoIIAB dacF pupG drm ripX yqzK
2,410,000bp
2,420,000bp
2,430,000bp
2,440,000bp
ansR yqkF yqkD yqzH yqjV yqjU proI yqjM yqjL zwf oxaAB yqzJ yqjF bmrU bmr bmrR
fur spoIIM yqkK mleA mleN ansB ansA yqxK nudF yqkE yqkB yqkC yqkA yqjY yqjZ polYB yqjX yqjT coaA dsdA yqjQ yqjP yqjN rnz gndA polYA yqjE yqjD yqjC yqjB yqjA artR artQ artP yqiW bkdB bkdAB bkdAA
2,460,000bp
2,470,000bp
2,480,000bp
2,490,000bp
yqzF yqiG yqhQ yqhP yqhL
lpdV buk bcd ptb bkdR prpB prpD prpC mmgC mmgB mmgA yqiK yqiI yqiH spo0A spoIVB recN ahrC yqxC dxs ispA xseA xseB folD nusB yqhY accC accB spoIIIAH spoIIIAG spoIIIAF spoIIIAE spoIIIAD spoIIIAC spoIIIAB spoIIIAA yqhV efp yqhT yqhS yqhR yqhO mntR yqhM gcvPB gcvPA gcvT
2,510,000bp
2,520,000bp
2,530,000bp
2,540,000bp
Experiment 1
Experiment 2
RpoC-His
RpoA-His
RpoA^int
RpoA^del
5.00
0.00

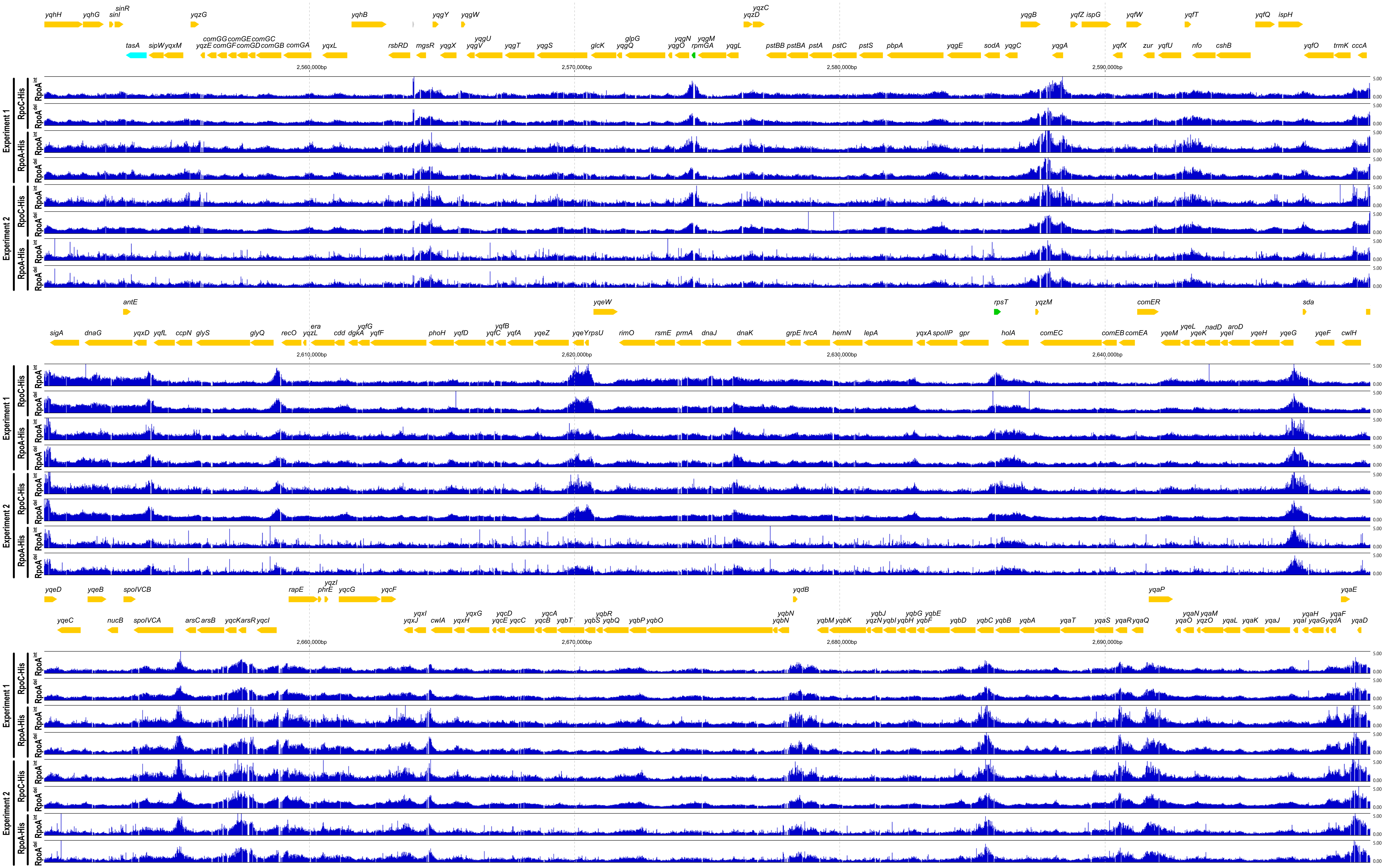

yqhH yqhG sinR sinI yqzG yqhB yqgY yqgW yqzC yqzD yqgB yqfZ ispG yqfW yqfT yqfQ ispH
tasA sipW yqxM comGG comGE comGC yqzE comGF comGD comGB comGA yqxL rsbRD mgsR yqgX yqgU yqgV yqgT yqgS glcK glpG yqgQ yqgN yqgO yqgM rpmGA yqgL pstBB pstBA pstA pstC pstS pbpA yqgE sodA yqgC yqgA yqfX zur yqfU nfo cshB yqfO trmK cccA
2,560,000bp
2,570,000bp
2,580,000bp
2,590,000bp
Experiment 1
Experiment 2
RpoC-His
RpoA-His
RpoA^int
RpoA^del
5.00
0.00
antE yqeW rpsT yqzM comER sda
sigA dnaG yqxD yqfL ccpN glyS glyQ recO era yqzL cdd dgkA yqfG yqfF phoH yqfD yqfB yqfC yqfA yqeZ yqeY rpsU rimO rsmE prmA dnaJ dnaK grpE hrcA hemN lepA yqxA spoIIP gpr holA comEC comEB comEA yqeM yqeL yqeK nadD yqeI aroD yqeH yqeG yqeF cwlH
2,610,000bp
2,620,000bp
2,630,000bp
2,640,000bp
yqeD yqeB spoIVCB rapE yqzI phrE yqcG yqcF yqdB yqaP yqaE
yqeC nucB spoIVCA arsC arsB yqcK arsR yqcI yqxI yqxJ cwlA yqxG yqxH yqcD yqcE yqcC yqcA yqcB yqbT yqbR yqbS yqbQ yqbP yqbO yqbN yqbM yqbK yqbJ yqzN yqbI yqbG yqbH yqbE yqbF yqbD yqbC yqbB yqbA yqaT yqaS yqaR yqaQ yqaN yqaM yqaO yqzO yqaL yqaK yqaJ yqaH yqaI yqaG yqaF yqdA yqaD
2,660,000bp
2,670,000bp
2,680,000bp
2,690,000bp

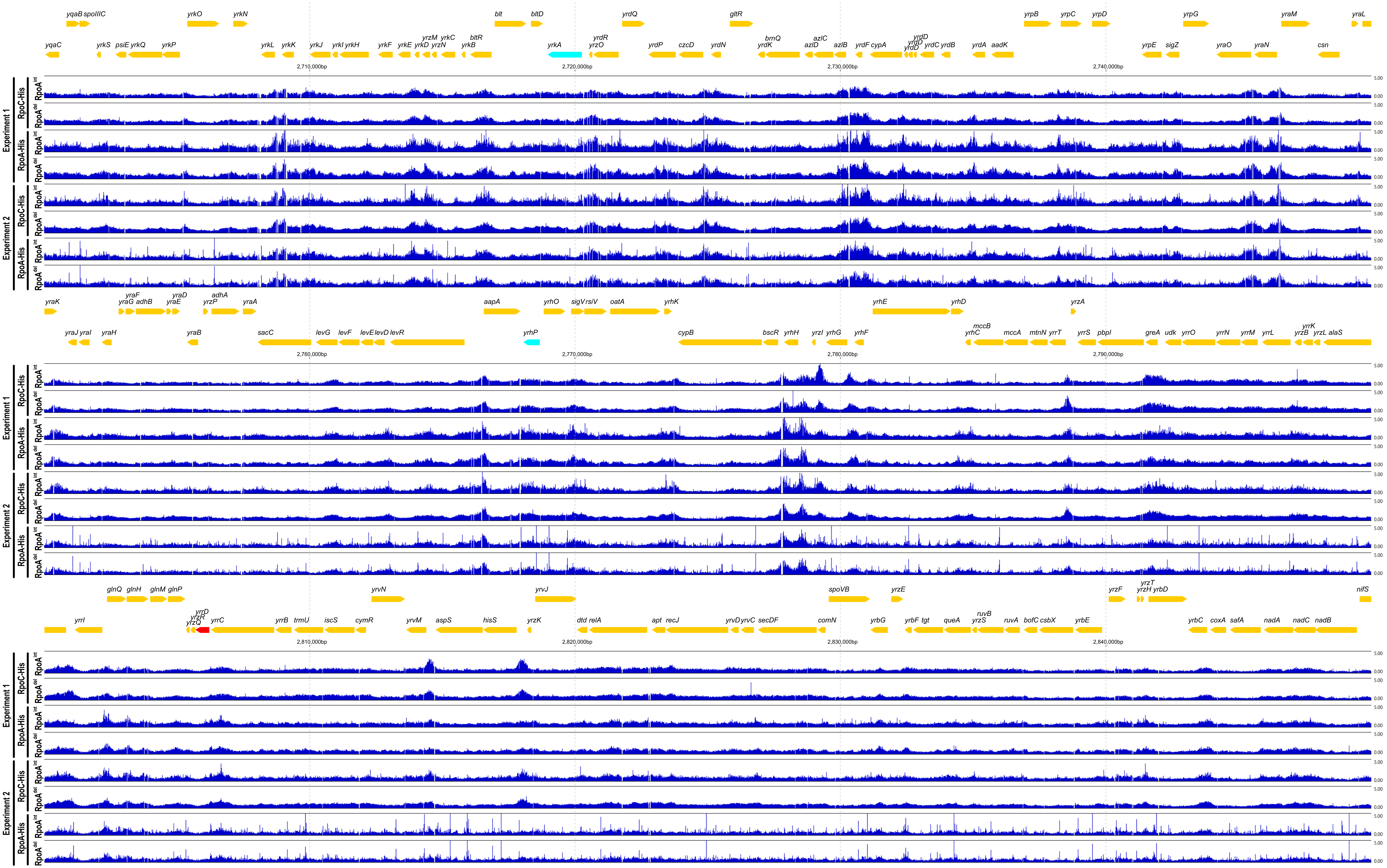
yqaB spoIIIC
yrkO
yrkN
blt
bltD
yrdQ
gltR
yrpB
yrpC
yrpD
yrpG
yraM
yraL
yqaC
yrkS
psiE yrkQ
yrkP
yrkL
yrkK
yrkJ
yrkI yrkH
yrkF
yrkE yrkD
yrzM yrkC
yrzN
bltR
yrkB
yrkA
yrdR
yrzO
yrdP
czcD
yrdN
brnQ
yrdK
azlC
azlD
azlB
yrdF cypA
yrdD
yrdC yrdB
yrdA
aadK
yrpE
sigZ
yraO
yraN
csn
2,710,000bp
2,720,000bp
2,730,000bp
2,740,000bp
Experiment 1
Experiment 2
RpoC-His
RpoA-His
RpoA[int]
RpoA[del]
5.00
0.00
yraK
yraF
yraG adhB
yraD
yraE
adhA
yrzP
yraA
aapA
yrhO
sigV rsiV
oatA
yrhK
yrhE
yrhD
yrzA
yraJ yraI
yraH
yraB
sacC
levG
levF
levE levD levR
yrhP
cypB
bscR
yrhH
yrzI yrhG
yrhF
mccB
yrhC
mccA
mtnN yrrT
yrrS
pbpI
greA
udk
yrrO
yrrN
yrrM
yrrL
yrrK
yrzB
yrzL alaS
2,760,000bp
2,770,000bp
2,780,000bp
2,790,000bp
glnQ
glnH
glnM glnP
yrvN
yrvJ
spoVB
yrzE
yrzF
yrzT
yrzH yrbD
nifS
yrrI
yrrD
yrzR
yrzQ
yrrC
yrrB
trmU
iscS
cymR
yrvM
aspS
hisS
yrzK
dtd relA
apt
recJ
yrvD yrvC
secDF
comN
yrbG
yrbF
tgt
queA
ruvB
yrzS
ruvA
bofC csbX
yrbE
yrbC
coxA
safA
nadA
nadC
nadB
2,810,000bp
2,820,000bp
2,830,000bp
2,840,000bp

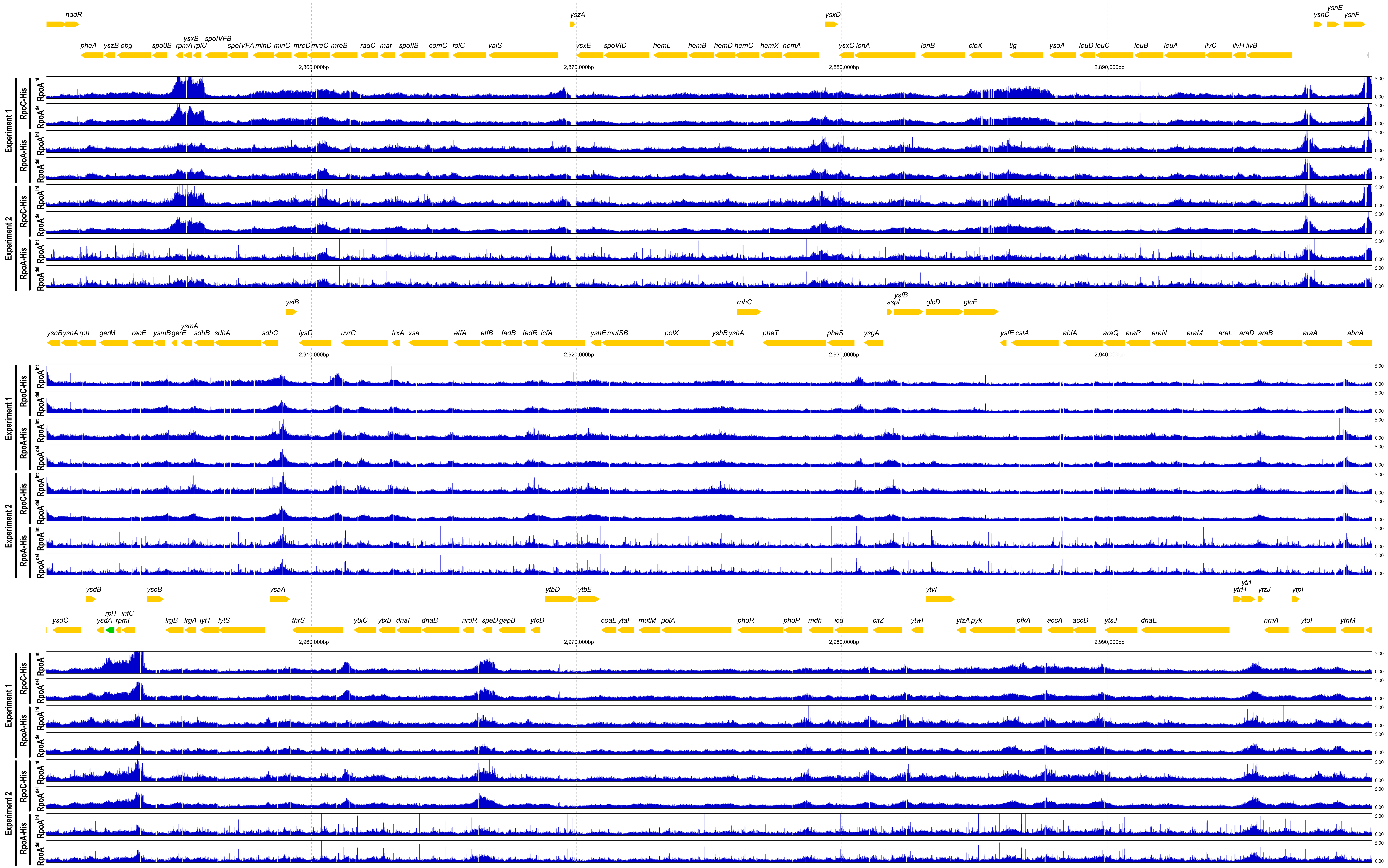
nadR
yszA
ysxD
ysnE
ysnD ysnF
pheA yszB obg spo0B ysxB rpmA rplU spoIVFB spoIVFA minD minC mreD mreC mreB radC maf spoIIB comC folC valS
ysxE spoVID hemL hemB hemD hemC hemX hemA
ysxC lonA lonB clpX tig ysoA leuD leuC leuB leuA ilvC ilvH ilvB
2,860,000bp
2,870,000bp
2,880,000bp
2,890,000bp
Experiment 1
Experiment 2
RpoC-His
RpoA-His
RpoA int
RpoA del
5.00
0.00
yslB
rhhC
ysfB
sspI glcD glcF
ysnB ysnA rph germ racE ysmB gerE ysmA sdhB sdhA sdhC lysC uvrC trxA xsa etfA etfB fadB fadR lcfA
yshE mutSB polX yshB yshA pheT pheS ysgA
ysfE cstA abfA araQ araP araN araM araL araD araB araA abnA
2,910,000bp
2,920,000bp
2,930,000bp
2,940,000bp
ysdB yscB ysaA ytbD ytbE ytvI
ytrI ytrH ytzJ ytpI
ysdC rplT ysdA infC rpmI lrgB lrgA lytT lytS thrS ytxC ytxB dnaI dnaB nrdR speD gapB ytcD
coaE ytaF mutM polA phoR phoP mdh icd citZ ytwI
ytzA pyk pfkA accA accD ytsJ dnaE nrnA ytoI ytnM
2,960,000bp
2,970,000bp
2,980,000bp
2,990,000bp

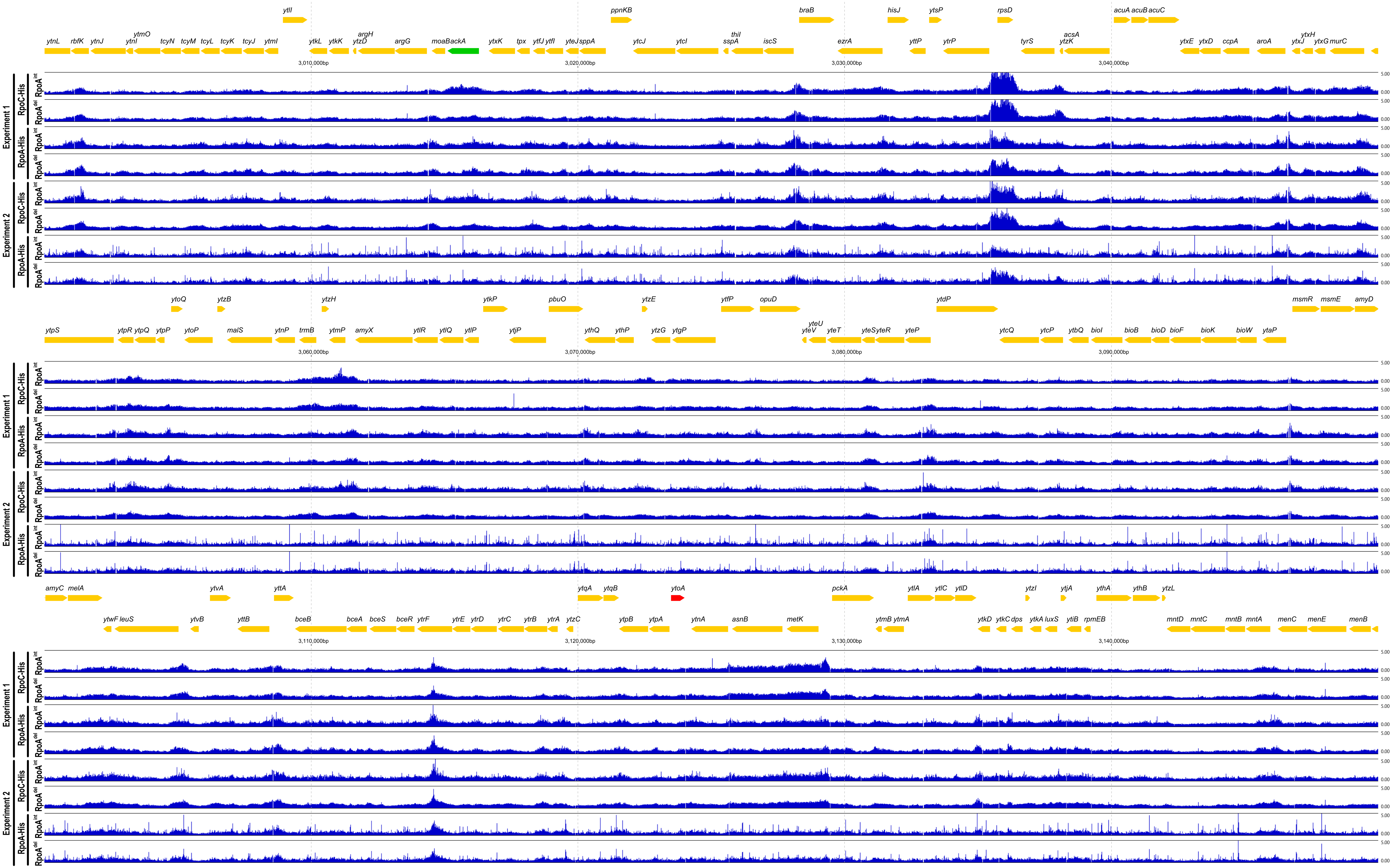

ytlI ppnKB braB hisJ ytsP rpsD acuA acuB acuC
ytnL rbfK ytnJ ytmO ytnI tcyN tcyM tcyL tcyK tcyJ ytmI ytkL ytkK argH ytzD argG moaBackA ytxK tpx ytfJ ytfI yteJ sppA ytcJ ytcI thiI sspA iscS ezrA yttP ytrP tyrS acsA ytzK ytxE ytxD ccpA aroA ytxH ytxJ ytxG murC
3,010,000bp
3,020,000bp
3,030,000bp
3,040,000bp
ytoQ ytzB ytzH ytkP pbuO ytzE ytfP opuD ytdP msmR msmE amyD
ytpS ytpR ytpQ ytpP ytoP malS ytnP trmB ytmP amyX ytlR ytlQ ytlP ytjP ythQ ythP ytzG ytgP yteU yteV yteT yteSyteR yteP ytcQ ytcP ytbQ bioI bioB bioD bioF bioK bioW ytaP
3,060,000bp
3,070,000bp
3,080,000bp
3,090,000bp
amyC melA ytvA yttA ytqA ytqB ytoA pckA ytlA ytlC ytlD ytzI ytjA ythA ythB ytzL
ytwF leuS ytvB yttB bceB bceA bceS bceR ytrF ytrE ytrD ytrC ytrB ytrA ytzC ytpB ytpA ytnA asnB metK ytmB ytmA ytkD ytkC dps ytkA luxS ytiB rpmEB mntD mntC mntB mntA menC menE menB
3,110,000bp
3,120,000bp
3,130,000bp
3,140,000bp
Experiment 1
Experiment 2
RpoC-His
RpoA-His
RpoA^int
RpoA^del
5.00
0.00

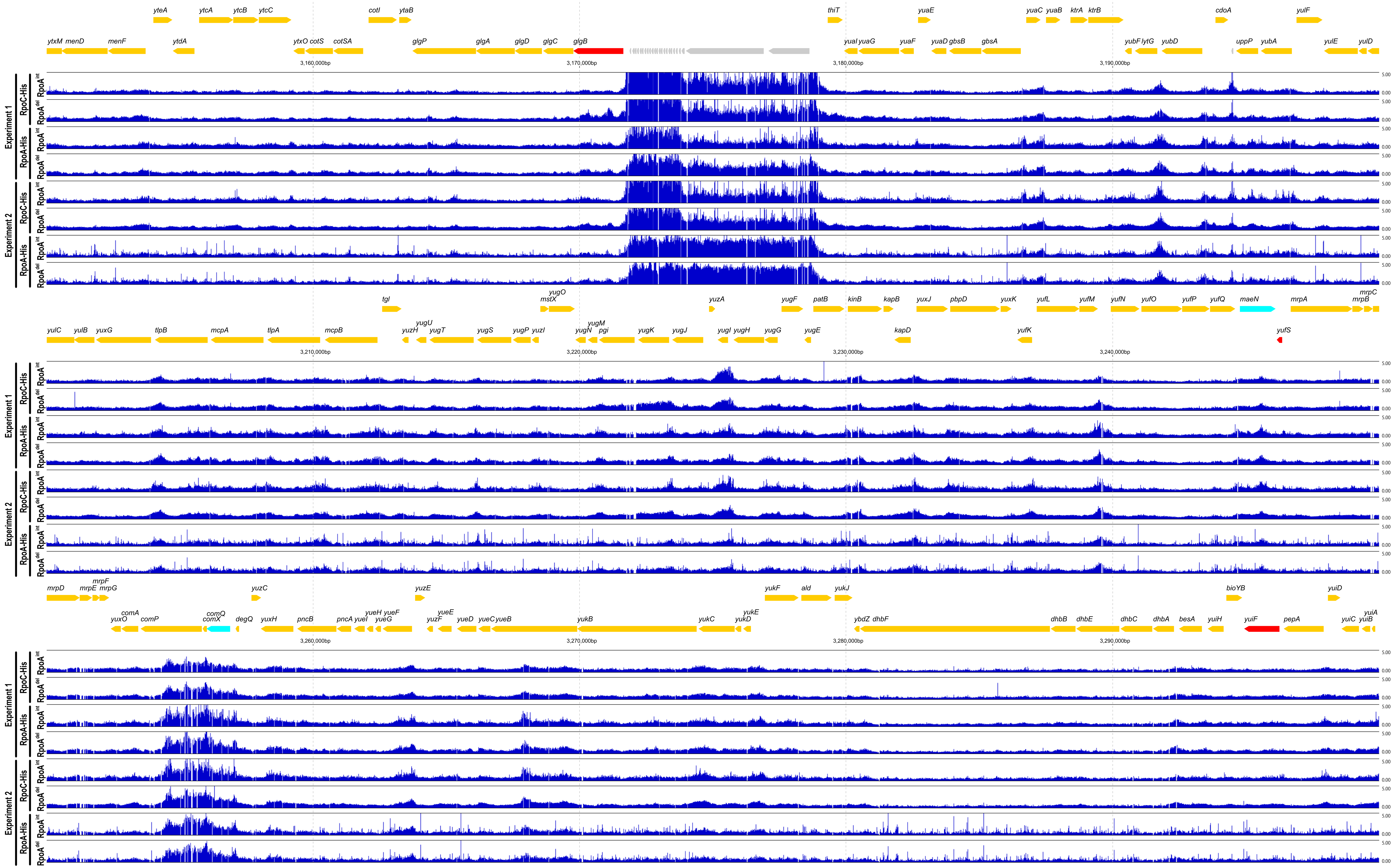
yteA ytcA ytcB ytcC cotI ytaB thiT yuaE yuaC yuaB ktrA ktrB cdoA yulF
ytxM menD menF ytdA ytxO cotS cotSA glgP glgA glgD glgC glgB yuaI yuaG yuaF yuaD gbsB gbsA yubF lytG yubD uppP yubA yulE yulD
3,160,000bp 3,170,000bp 3,180,000bp 3,190,000bp
tgl yugO mstX yuzA yugF patB kinB kapB yuxJ pbpD yuxK yufL yufM yufN yufO yufP yufQ maeN mrpA mrpC mrpB
yulC yulB yuxG tlpB mcpA tlpA mcpB yugU yuzH yugT yugS yugP yuzI yugM yugN pgi yugK yugJ yugI yugH yugG yugE kapD yufK yufS
3,210,000bp 3,220,000bp 3,230,000bp 3,240,000bp
mrpD mrpF mrpE mrpG yuzC yuzE yukF ald yukJ bioYB yuiD
comA yuxO comP comQ comX degQ yuxH pncB pncA yueI yueH yueF yueG yueE yuzF yueD yueC yueB yukB yukC yukE yukD ybdZ dhbF dhbB dhbE dhbC dhbA besA yuiH yuiF pepA yuiA yuiC yuiB
3,260,000bp 3,270,000bp 3,280,000bp 3,290,000bp
Experiment 1
Experiment 2
RpoC-His
RpoA-His
RpoA^int
RpoA^del
5.00
0.00

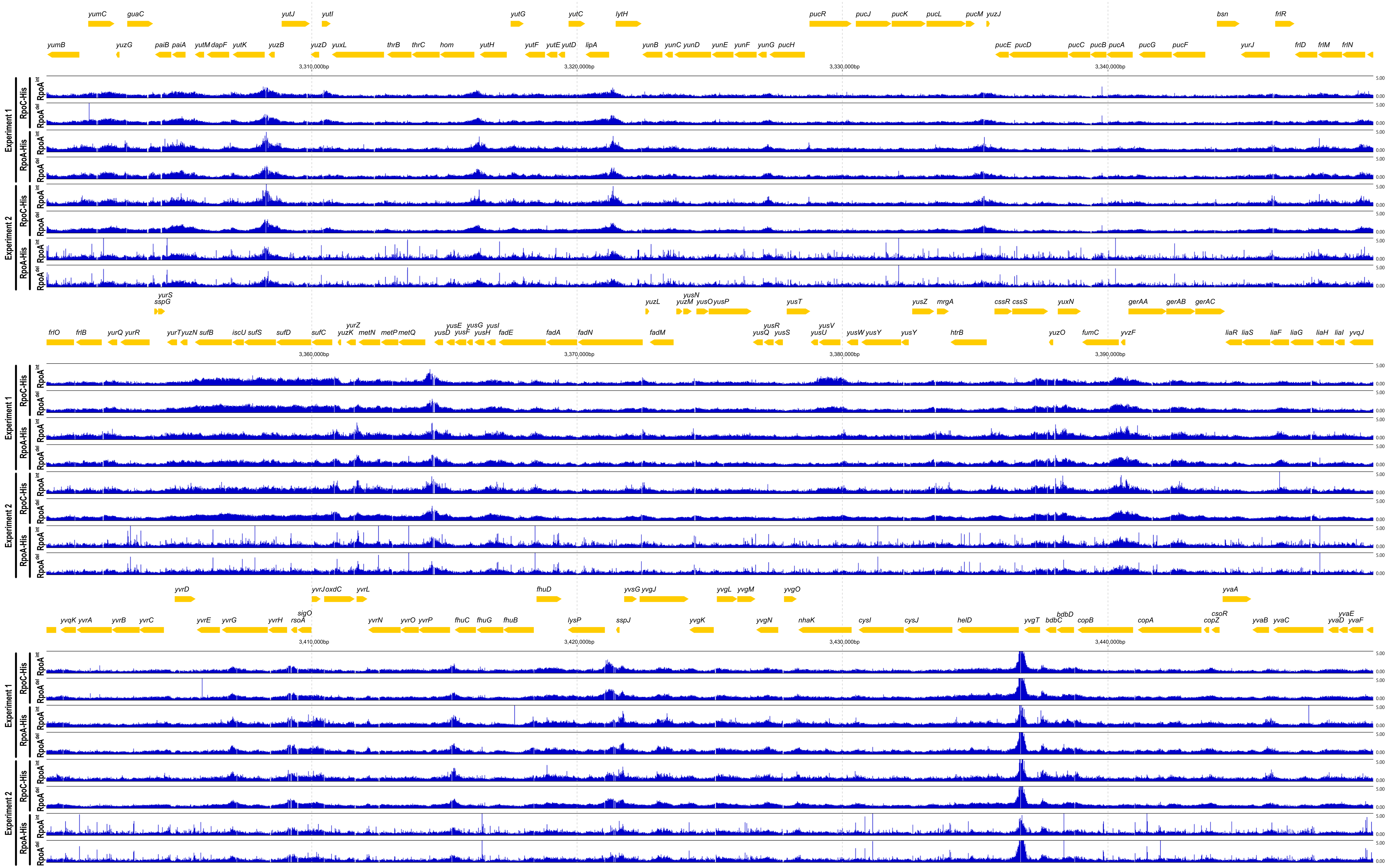
yumC guaC yutJ yutI yutG yutC lytH pucR pucJ pucK pucL pucM yuzJ bsn frlR
yumB yuzG paiB paiA yutM dapF yutK yuzB yuzD yuxL thrB thrC hom yutH yutF yutE yutD lipA yunB yunC yunD yunE yunF yunG pucH pucE pucD pucC pucB pucA pucG pucF yurJ frlD frlM frlN
3,310,000bp
3,320,000bp
3,330,000bp
3,340,000bp
Experiment 1
Experiment 2
RpoC-His
RpoA-His
RpoA^int
RpoA^del
5.00
0.00
yurS sspG yuzL yusN yuzM yusO yusP yusT yusZ mrgA cssR cssS yuxN gerAA gerAB gerAC
frlO frlB yurQ yurR yurT yuzN sufB iscU sufS sufD sufC yurZ yuzK metN metP metQ yusE yusD yusF yusG yusH yusI fadE fadA fadN fadM yusR yusQ yusS yusV yusU yusW yusY yusY htrB yuzO fumC yvzF liaR liaS liaF liaG liaH liaI yvqJ
3,360,000bp
3,370,000bp
3,380,000bp
3,390,000bp
yvrD yvrJ oxdC yvrL fhuD yvsG yvgJ yvgL yvgM yvgO yvaA
yvqK yvrA yvrB yvrC yvrE yvrG yvrH sigO rsoA yvrN yvrO yvrP fhuC fhuG fhuB lysP sspJ yvgK yvgN nhaK cysI cysJ helD yvgT bdbD bdbC copB copA csoR copZ yvaB yvaC yvaE yvaD yvaF
3,410,000bp
3,420,000bp
3,430,000bp
3,440,000bp

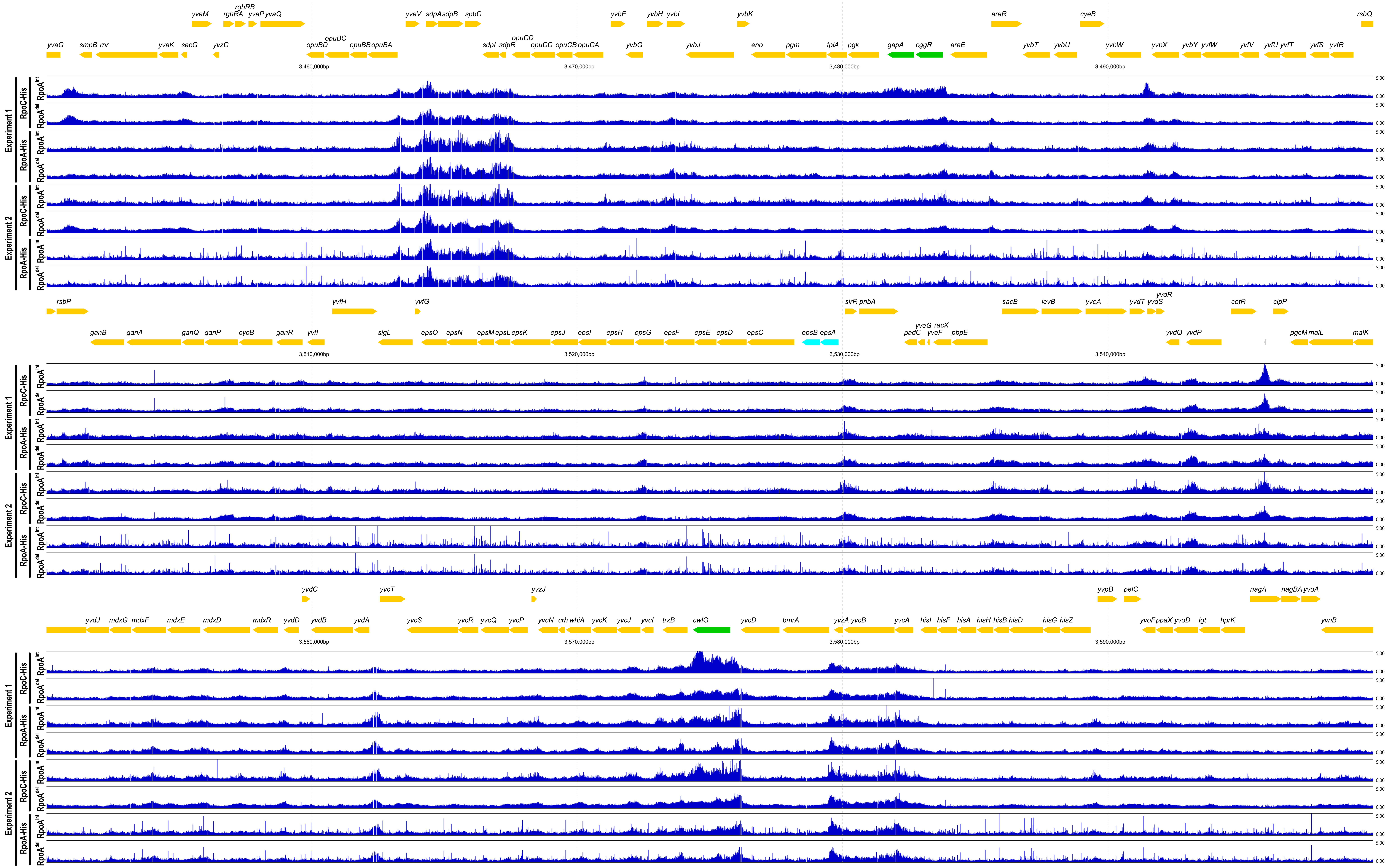
rghRB
yvaM rghRA yvaP yvaQ yvaV sdpA sdpB spbC yvbF yvbH yvbI yvbK araR cyeB rsbQ
opuBC opuCD
yvaG smpB rnr yvaK secG yvzC opuBD opuBB opuBA sdpI sdpR opuCC opuCB opuCA yvbG yvbJ eno pgm tpiA pgk gapA cggR araE yvbT yvbU yvbW yvbX yvbY yvfW yvfV yvfU yvfT yvfS yvfR
3,460,000bp 3,470,000bp 3,480,000bp 3,490,000bp
Experiment 1
Experiment 2
RpoC-His
RpoA-His
RpoA^int
RpoA^del
5.00
0.00
rsbP yvfH yvfG slrR pnbA sacB levB yveA yvdT yvdS yvdR cotR clpP
yveG racX
ganB ganA ganQ ganP cycB ganR yvfI sigL epsO epsN epsM epsL epsK epsJ epsI epsH epsG epsF epsE epsD epsC epsB epsA padC yveF pbpE yvdQ yvdP pgcM malL malK
3,510,000bp 3,520,000bp 3,530,000bp 3,540,000bp
yvdC yvcT yvzJ yvpB pelC nagA nagBA yvoA
yvdJ mdxG mdxF mdxE mdxD mdxR yvdD yvdB yvdA yvcS yvcR yvcQ yvcP yvcN crh whiA yvcK yvcJ yvcI trxB cwlO yvcD bmrA yvzA yvcB yvcA hisI hisF hisA hisH hisB hisD hisG hisZ yvoF ppaX yvoD lgt hprK yvnB
3,560,000bp 3,570,000bp 3,580,000bp 3,590,000bp

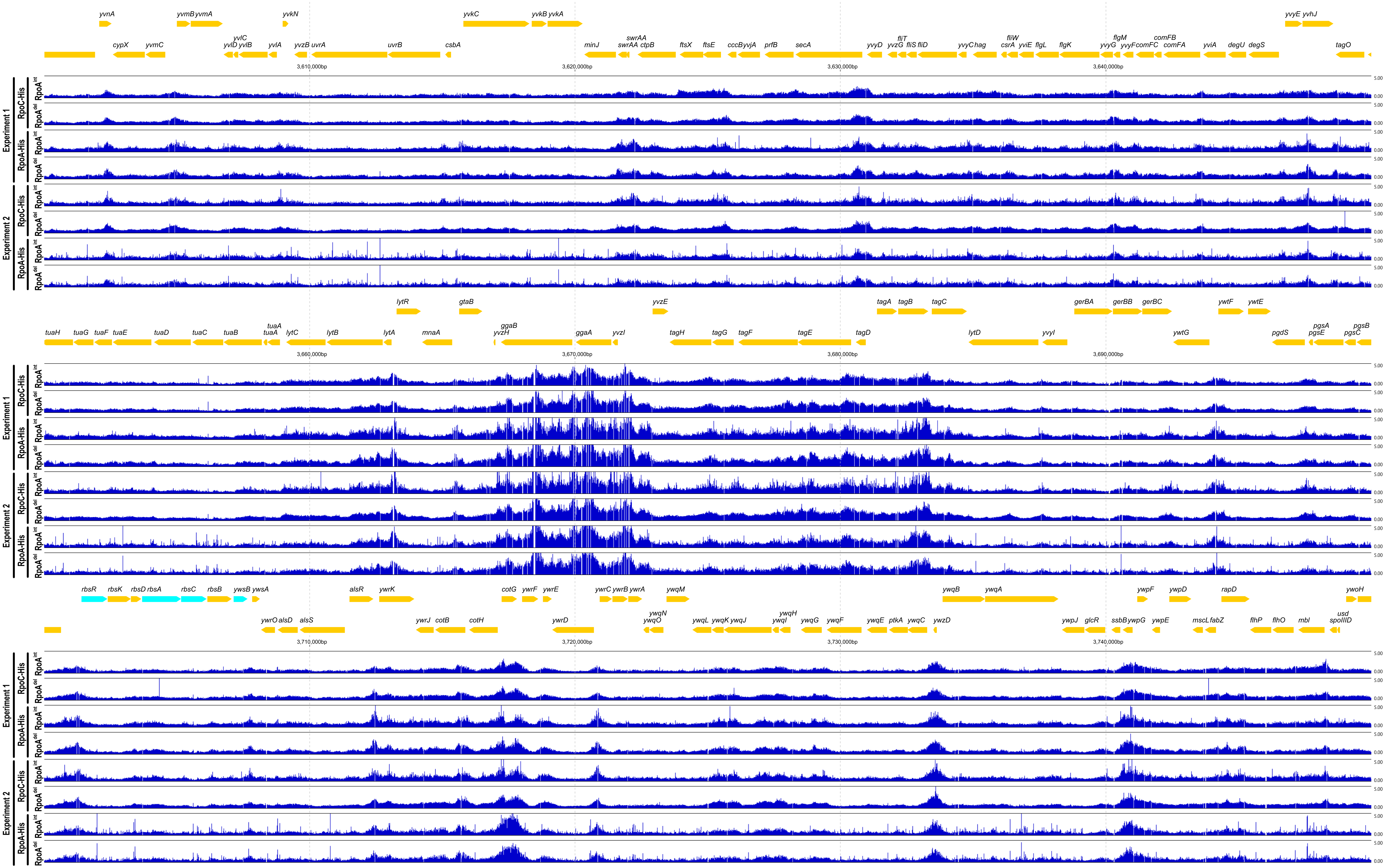

yvnA yvmB yvmA yvkN yvkC yvkB yvkA yvyE yvhJ
cypX yvmC yvlC yvlD yvlB yvlA yvzB uvrA uvrB csbA minJ swrAA swrAA ctpB ftsX ftsE cccB yvjA prfB secA yvyD fliT yvzG fliS fliD yvyC hag fliW csrA yviE flgL flgK flgM yvyG yvyF comFB comFC comFA yviA degU degS tagO
3,610,000bp
3,620,000bp
3,630,000bp
3,640,000bp
lytR gtaB yvzE tagA tagB tagC gerBA gerBB gerBC ywtF ywtE
tuaH tuaG tuaF tuaE tuaD tuaC tuaB tuaA tuaA lytC lytB lytA mnaA ggaB yvzH ggaA yvzI tagH tagG tagF tagE tagD lytD yvyI ywtG pgdS pgsA pgsE pgsB pgsC
3,660,000bp
3,670,000bp
3,680,000bp
3,690,000bp
rbsR rbsK rbsD rbsA rbsC rbsB ywsB ywsA alsR ywrK cotG ywrF ywrE ywrC ywrB ywrA ywqM ywqB ywqA ywpF ywpD rapD ywoH
ywrO alsD alsS ywrJ cotB cotH ywrD ywqN ywqO ywqL ywqK ywqJ ywqH ywqI ywqG ywqF ywqE ptkA ywqC ywzD ywpJ glcR ssbB ywpG ywpE mscL fabZ flhP flhO mbl usd spoIIID
3,710,000bp
3,720,000bp
3,730,000bp
3,740,000bp
Experiment 1
Experiment 2
RpoC-His
RpoA-His
RpoA^int
RpoA^del
5.00
0.00

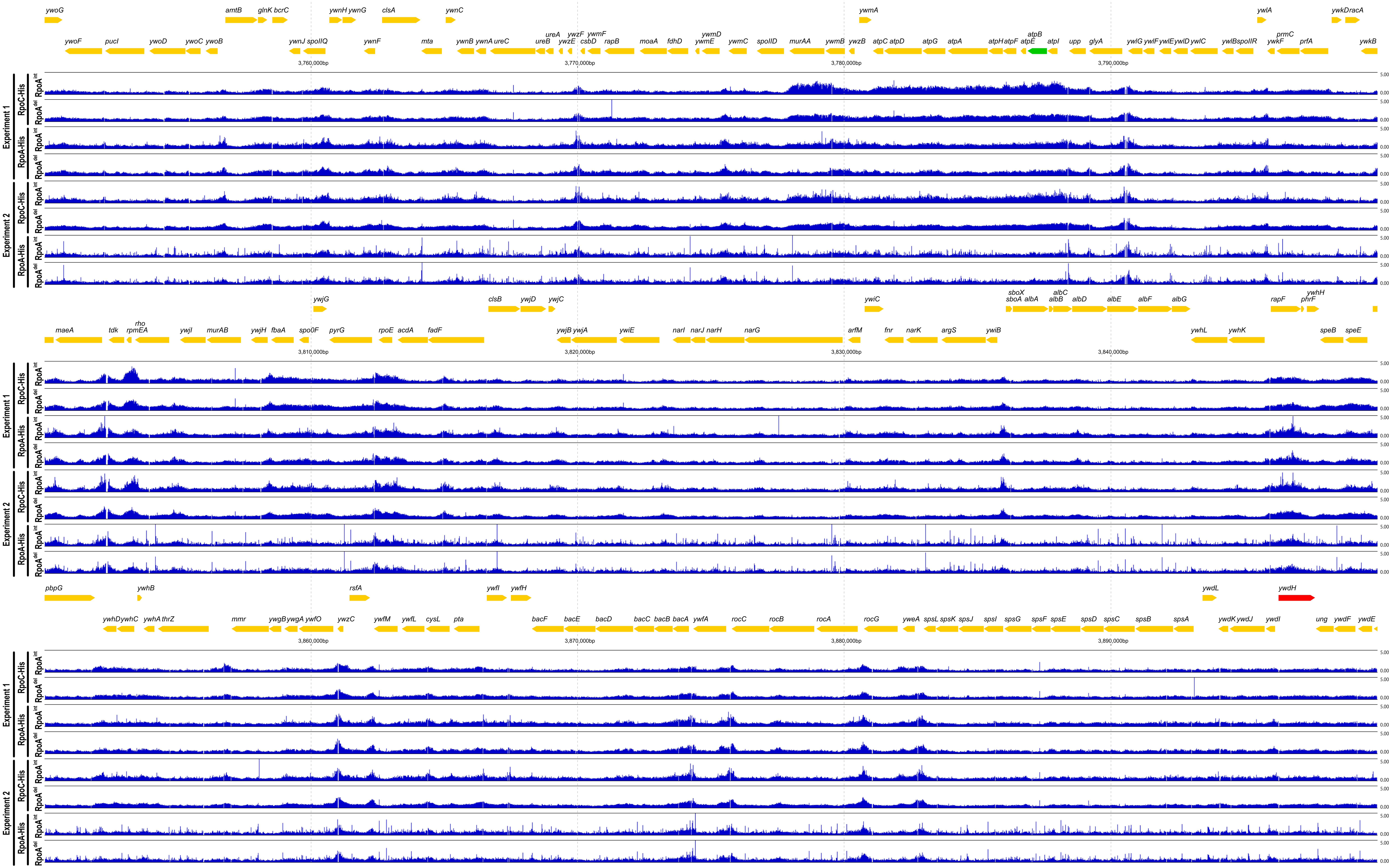
ywoG
amtB
glnK bcrC
ywnH ywnG
clsA
ywnC
ywmA
ywlA
ywkDracA
ywoF
pucI
ywoD
ywoC ywoB
ywnJ spoIIQ
ywnF
mta
ywnB ywnA ureC
ureA
ureB
ywzF ywmF
ywzE csbD
rapB
moaA
fdhD
ywmD
ywmE
ywmC
spoIID
murAA
ywmB
ywzB
atpC atpD
atpG
atpA
atpB
atpH atpF atpE
atpI
upp
glyA
ywlG ywlF ywlE ywlD ywlC
ywlB spoIIR
prmC
ywkF
prfA
ywkB
3,760,000bp
3,770,000bp
3,780,000bp
3,790,000bp
Experiment 1
Experiment 2
RpoC-His
RpoA-His
RpoA^int
RpoA^del
5.00
0.00
ywjG
clsB
ywjD
ywjC
ywiC
sboX
sboA albA
albC
albB
albD
albE
albF
albG
rapF
ywhH
phrF
maeA
tdk
rho
rpmEA
ywjI
murAB
ywjH
fbaA
spo0F
pyrG
rpoE
acdA
fadF
ywjB ywjA
ywiE
narI
narJ narH
narG
arfM
fnr
narK
argS
ywiB
ywhL
ywhK
speB
speE
3,810,000bp
3,820,000bp
3,830,000bp
3,840,000bp
pbpG
ywhB
rsfA
ywfI
ywfH
ywdL
ywdH
ywhD ywhC
ywhA thrZ
mmr
ywgB ywgA ywfO
ywzC
ywfM
ywfL
cysL
pta
bacF
bacE
bacD
bacC bacB bacA
ywfA
rocC
rocB
rocA
rocG
yweA
spsL spsK spsJ
spsI
spsG
spsF
spsE
spsD
spsC
spsB
spsA
ywdK ywdJ
ywdI
ung
ywdF
ywdE
3,860,000bp
3,870,000bp
3,880,000bp
3,890,000bp

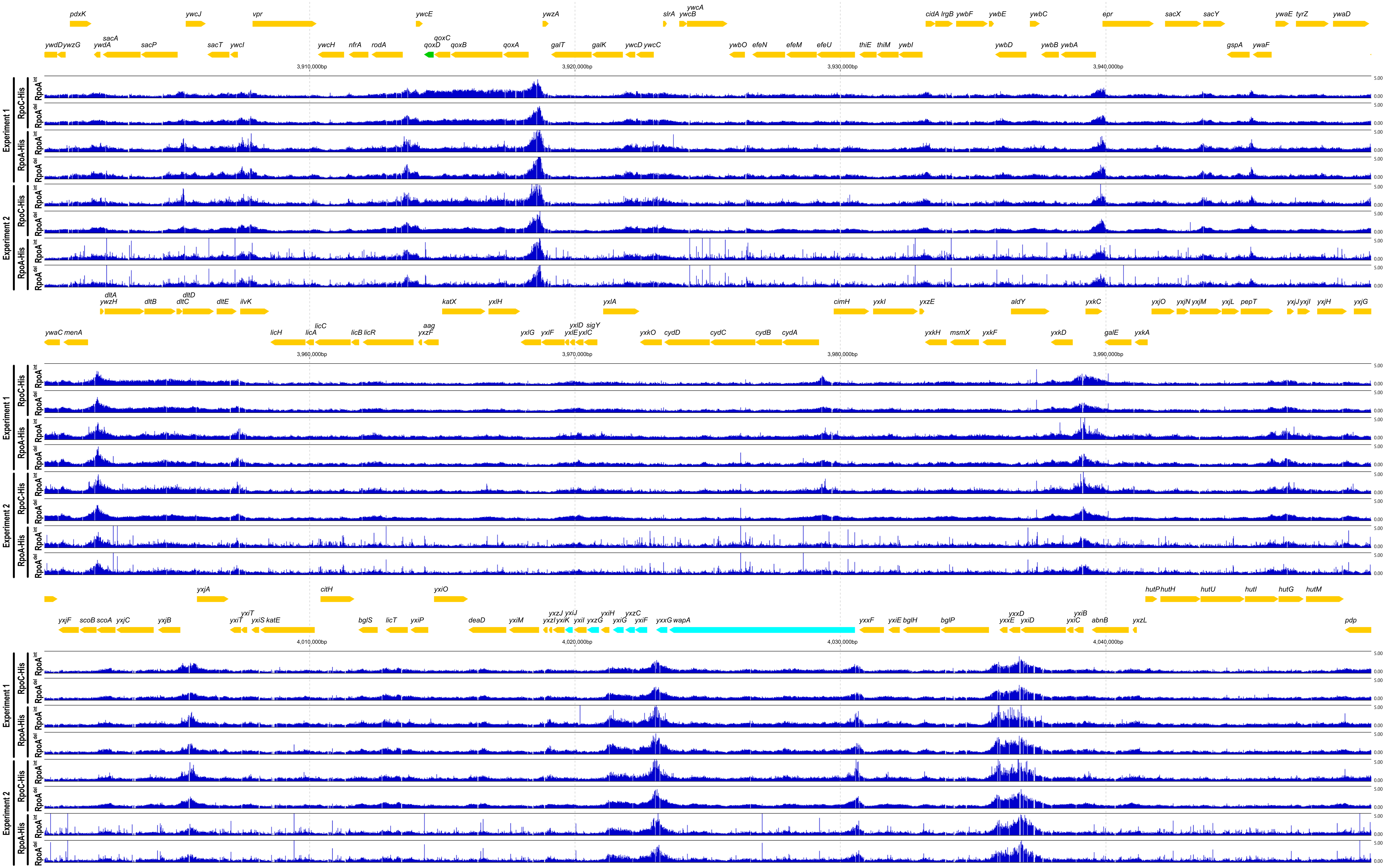

pdxK ywcJ vpr ywcE ywzA slrA ywcA ywcB cidA lrgB ywbF ywbE ywbC epr sacX sacY ywaE tyrZ ywaD
ywdD ywzG sacA ywdA sacP sacT ywcI ywcH nfrA rodA qoxC qoxD qoxB qoxA galT galK ywcD ywcC ywbO efeN efeM efeU thiE thiM ywbI ywbD ywbB ywbA gspA ywaF
3,910,000bp
3,920,000bp
3,930,000bp
3,940,000bp
dltA ywzH dltB dltD dltC dltE ilvK katX yxlH yxlA cimH yxkI yxzE aldY yxkC yxjO yxjN yxjM yxjL pepT yxjJ yxjI yxjH yxjG
ywaC menA licH licC licA licB licR aag yxzF yxlG yxlF yxlD sigY yxlE yxlC yxkO cydD cydC cydB cydA yxkH msmX yxkF yxkD galE yxkA
3,960,000bp
3,970,000bp
3,980,000bp
3,990,000bp
yxjA citH yxiO hutP hutH hutU hutI hutG hutM
yxjF scoB scoA yxjC yxjB yxiT yxiT yxiS katE bglS licT yxiP deaD yxiM yxzJ yxiJ yxzI yxiK yxiI yxzG yxiH yxiG yxzC yxiF yxxG wapA yxxF yxiE bglH bglP yxxD yxxE yxiD yxiB yxiC abnB yxzL pdp
4,010,000bp
4,020,000bp
4,030,000bp
4,040,000bp
Experiment 1
Experiment 2
RpoC-His
RpoA-His
RpoA^int
RpoA^del
5.00
0.00

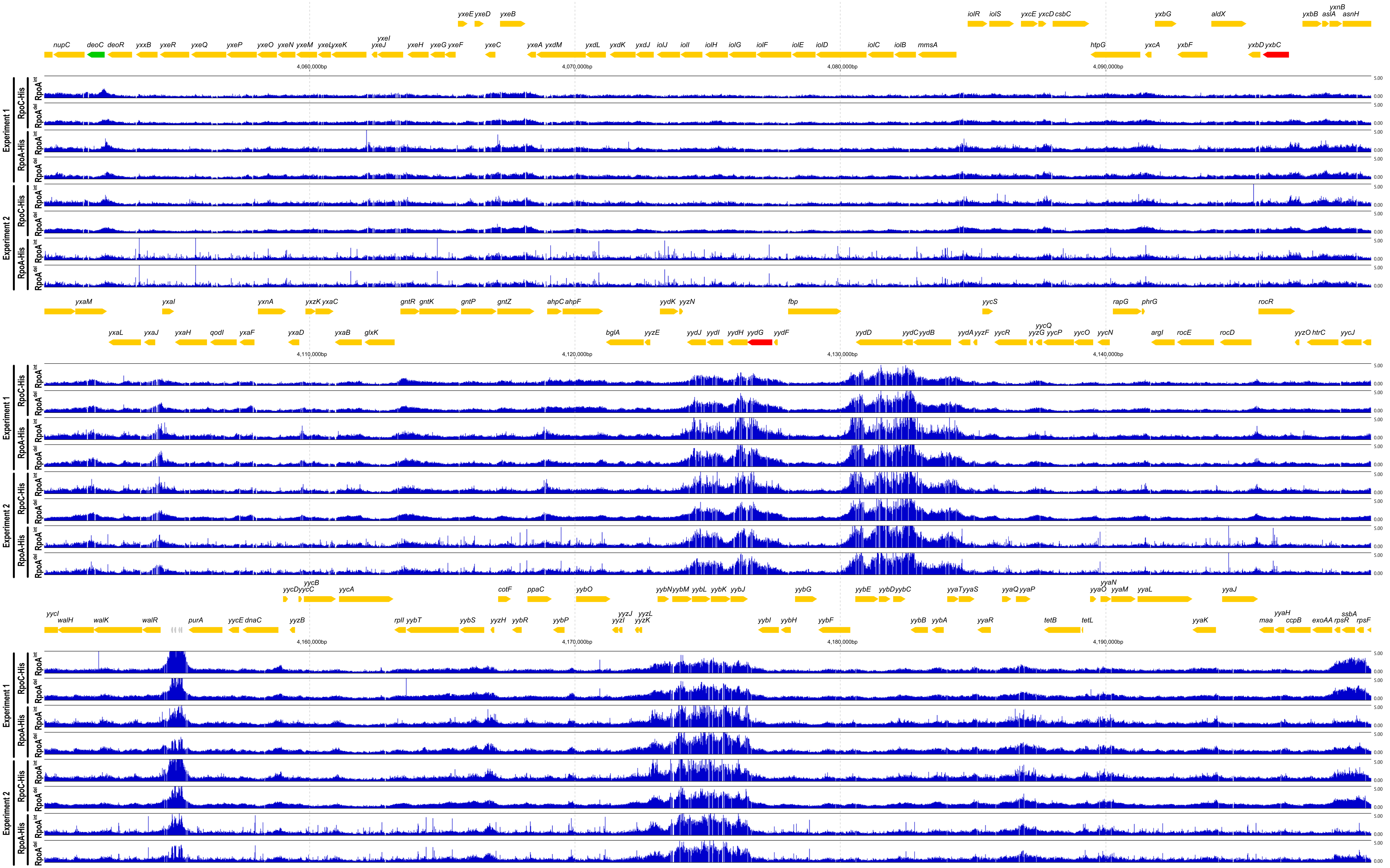

yxeE yxeD yxeB iolR iolS yxcE yxcD csbC yxbG aldX yxnB yxbB asIA asnH
nupC deoC deoR yxxB yxeR yxeQ yxeP yxeO yxeN yxeM yxeL yxeK yxeI yxeJ yxeH yxeG yxeF yxeC yxeA yxdM yxdL yxdK yxdJ iolJ iolI iolH iolG iolF iolE iolD iolC iolB mmsA htpG yxcA yxbF yxbD yxbC
4,060,000bp
4,070,000bp
4,080,000bp
4,090,000bp
Experiment 1
Experiment 2
RpoC-His
RpoA-His
RpoA^int
RpoA^del
5.00
0.00
yxaM yxaI yxnA yxzK yxaC gntR gntK gntP gntZ ahpC ahpF yydK yyzN fbp yycS rapG phrG rocR
yxaL yxaJ yxaH qodI yxaF yxaD yxaB glxK bglA yyzE yydJ yydI yydH yydG yydF yydD yydC yydB yydA yyzF yycR yycQ yyzG yycP yycO yycN argI rocE rocD yyzO htrC yycJ
4,110,000bp
4,120,000bp
4,130,000bp
4,140,000bp
Experiment 1
Experiment 2
RpoC-His
RpoA-His
RpoA^int
RpoA^del
yycB yycD yycC yycA cotF ppaC yybO yybN yybM yybL yybK yybJ yybG yybE yybD yybC yyaT yyaS yyaQ yyaP yyaN yyaO yyaM yyaL yyaJ
yycI walH walK walR purA yycE dnaC yyzB rplI yybT yybS yyzH yybR yybP yyzJ yyzI yyzL yyzK yybI yybH yybF yybB yybA yyaR tetB tetL yyaK yyaH maa ccpB exoAA rpsR ssbA rpsF
4,160,000bp
4,170,000bp
4,180,000bp
4,190,000bp
Experiment 1
Experiment 2
RpoC-His
RpoA-His
RpoA^int
RpoA^del

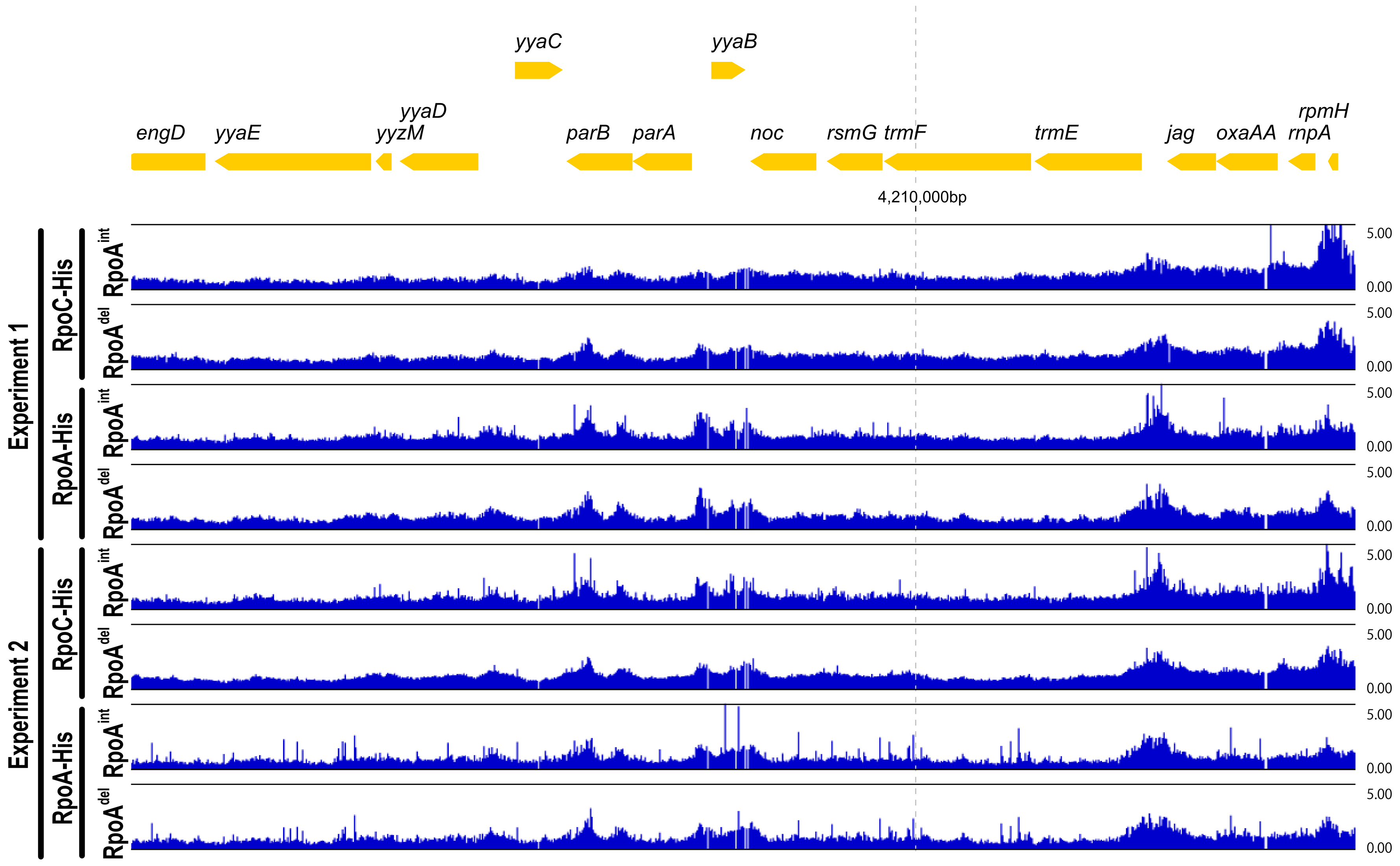
yyaC
yyaB
yyaD
rpmH
engD
yyaE
yyzM
parB
parA
noc
rsmG
trmF
trmE
jag
oxaAA
rnpA
4,210,000bp
Experiment 1
Experiment 2
RpoC-His
RpoA-His
RpoA^int
RpoA^del
5.00
0.00

S10. Fig. Genome-wide RNAP binding profiles determined by ChAP-chip analysis. RpoA^{int}-His, RpoA^{del}-His and RpoC-His were used as bait for the purification of RNAP complexes. The gene arrangement on the *B. subtilis* chromosome is shown by thick arrows at the top of the figure. Colors: sky blue indicates genes that are down-regulated in *rpoA*^{del}-expressing cells compared with *rpoA*^{int}-expressing cells, as determined by transcriptomic analysis; green indicates the top 50 genes showing the greatest reductions in RNAP binding among *rpoA*^{del}-expressing cells versus *rpoA*^{int}-expressing cells; and dark blue indicates the genes that showed both transcriptomic down-regulation and high-level reductions in RNAP binding in *rpoA*^{del}-expressing cells.
