# Supplementary material for: The Role of α-CTD in the Genome-Wide Transcriptional Regulation of the Bacillus subtilis Cells
Source: PLoS One. 2015 Jul 8;10(7):e0131588. doi: 10.1371/journal.pone.0131588 (PMC4495994; doi:10.1371/journal.pone.0131588)
Supplement: S11 Fig — The pathway for phosphorylation in the presence of glucose is indicated by red arrows, while that for phosphorylation in the absence of glucose is indicated by black arrows. The phosphoenolpyruvate-carbohydrate phosphotransferase system (PTS) is shown in the middle of the figure, with EI (enzyme I), HPr (histidine protein) and EII (enzyme II) shown in the blue square. The phosphorylated forms of HPr and PRD (the PTS-regulatory domain) containing activator are indicated with the circled-P notation. Unphosphorylated HPr is phosphorylated by HPr kinase (HPrK) in the presence of glucose, or by EI in the absence of glucose. The genes that showed mRNA down-regulation and major reductions in RNAP binding in rpoA del-expressing cells and are known to be repressed by CcpA and activated by ManR and MtlR are indicated at the bottom left (the expression of those genes are repressed by CcpA) and the bottom right (the expression of those genes are activated by PRD containing activators, MtlR and ManR) of the figure. (PDF) [file pone.0131588.s011.pdf]

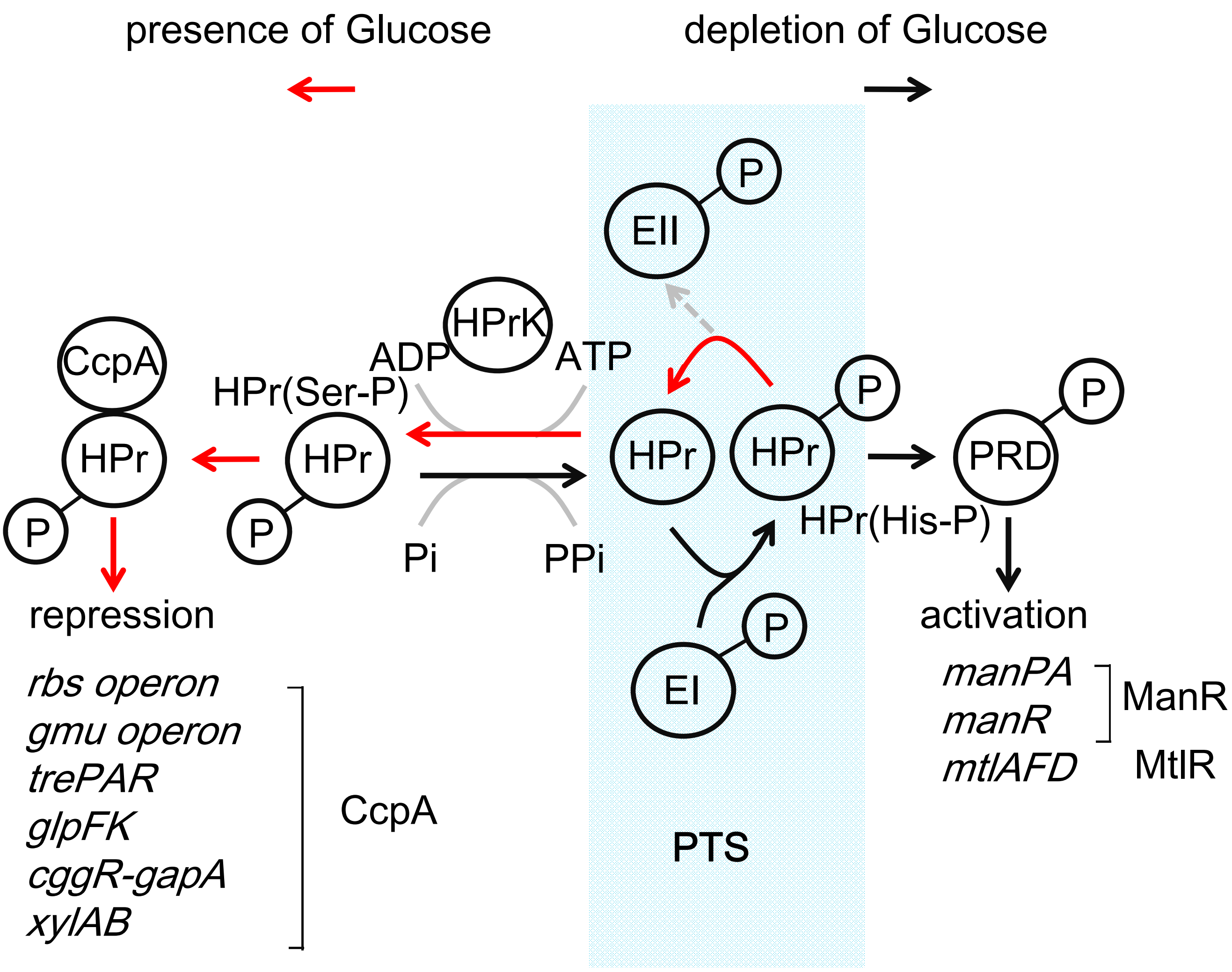

presence of Glucose
depletion of Glucose
EII
P
HPrK
ADP
ATP
CcpA
HPr(Ser-P)
HPr
P
Pi
PPi
PRD
HPr(His-P)
EI
repression
activation
rbs operon
gmu operon
trePAR
glpFK
cggR-gapA
xylAB
CcpA
PTS
manPA
manR
mtlAFD
ManR
MtlR

S11 Fig.

S11. Fig. The expression of genes (TUs) down-regulated or highly decreased in RNAP binding in *rpoA*^{del}-expressing cells are regulated by the carbon catabolite repression in *B. subtilis*. The pathway for phosphorylation in the presence of glucose is indicated by red arrows, while that for phosphorylation in the absence of glucose is indicated by black arrows. The phosphoenolpyruvate-carbohydrate phosphotransferase system (PTS) is shown in the middle of the figure, with EI (enzyme I), HPr (histidine protein) and EII (enzyme II) shown in the blue square. The phosphorylated forms of HPr and PRD (the PTS-regulatory domain) containing activator are indicated with the circled-P notation. Unphosphorylated HPr is phosphorylated by HPr kinase (HPrK) in the presence of glucose, or by EI in the absence of glucose. The genes that showed mRNA down-regulation and major reductions in RNAP binding in *rpoA*^{del}-expressing cells and are known to be repressed by CcpA and activated by ManR and MtlR are indicated at the bottom left (the expression of those genes are repressed by CcpA) and the bottom right (the expression of those genes are activated by PRD containing activators, MtlR and ManR) of the figure.

S11 Fig.
