# Supplementary material for: The Role of α-CTD in the Genome-Wide Transcriptional Regulation of the Bacillus subtilis Cells
Source: PLoS One. 2015 Jul 8;10(7):e0131588. doi: 10.1371/journal.pone.0131588 (PMC4495994; doi:10.1371/journal.pone.0131588)
Supplement: S12 Fig — Schematic representation of the primers used to amplify B. subtilis chromosomal and plasmid sequences (fragments A to P). Black bars indicate the complementary primer sequences used for recombinant PCR. (PDF) [file pone.0131588.s012.pdf]

SMS01

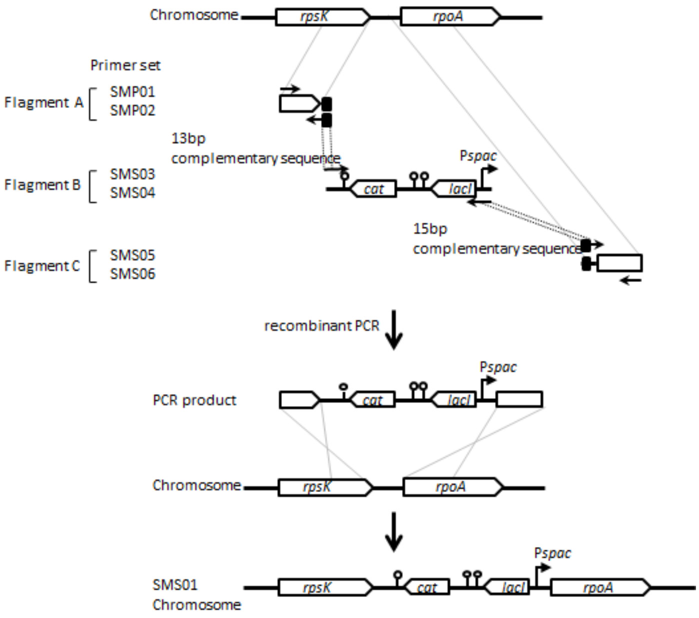

SMS02

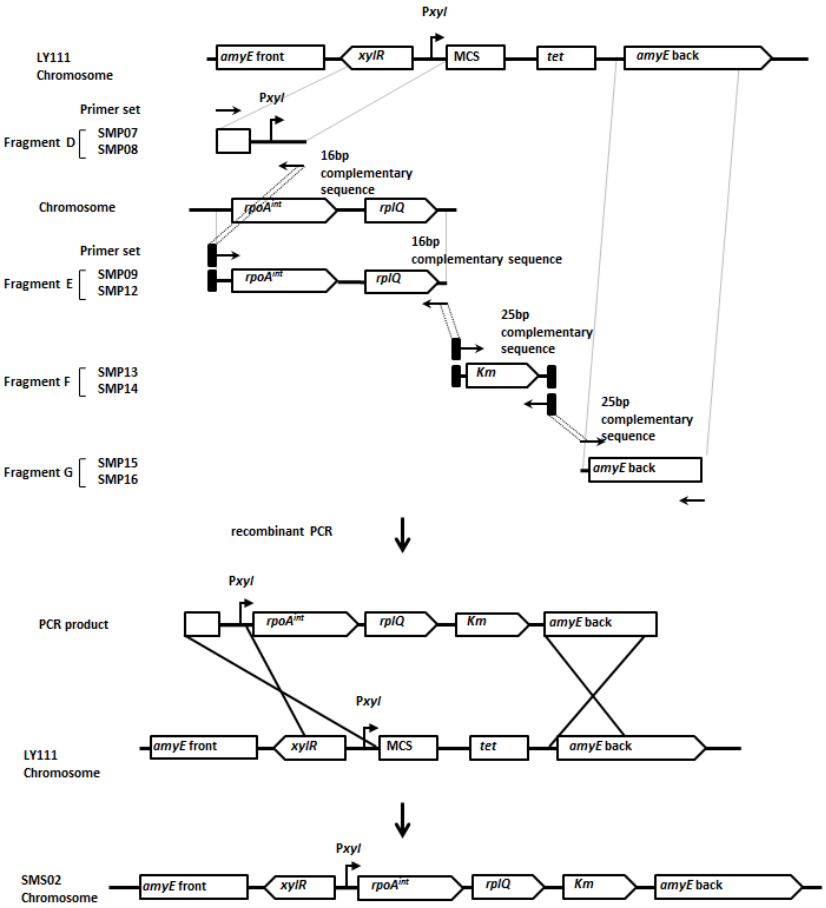

S12-1 Fig

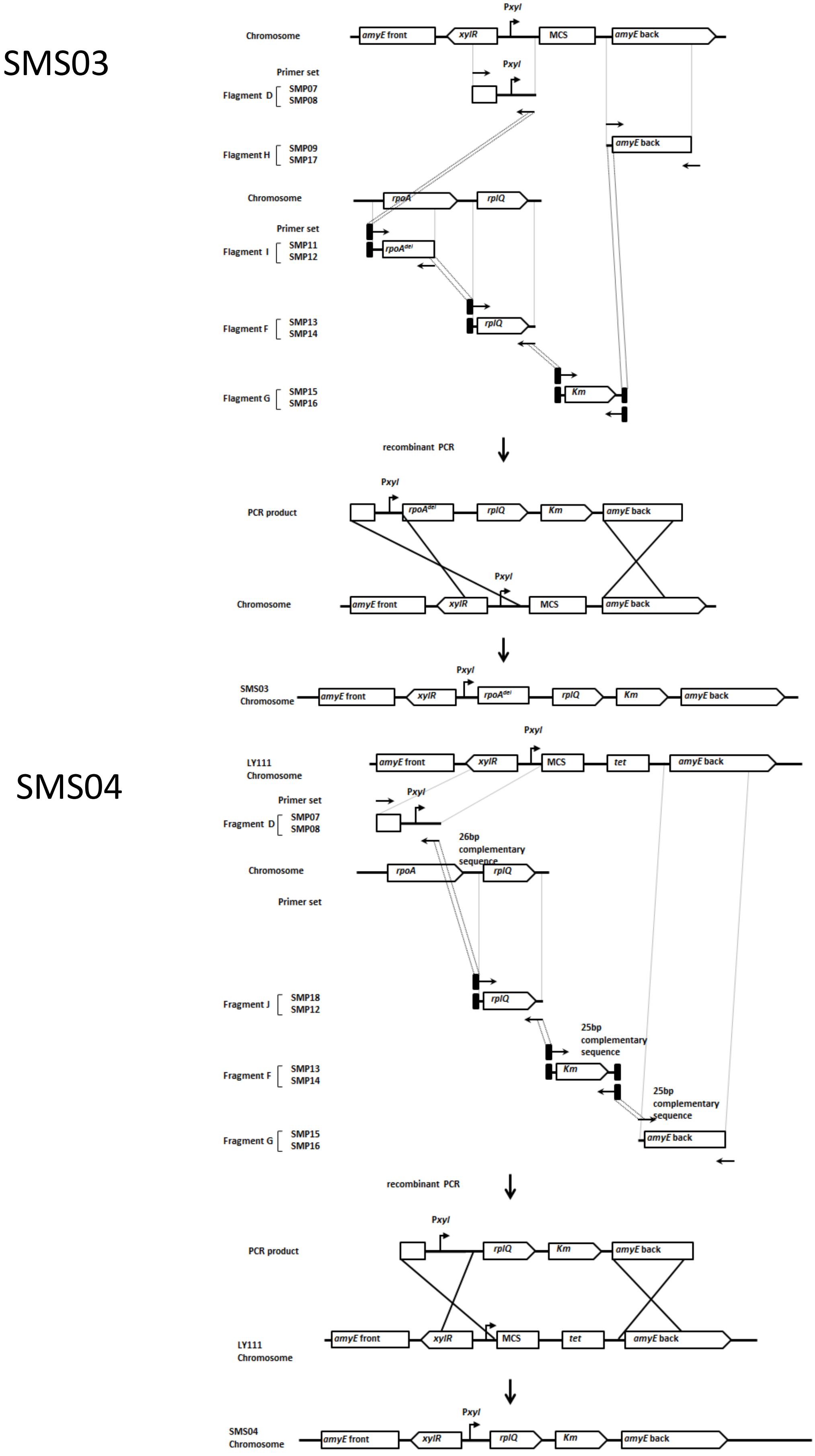
SMS03
Chromosome
amyE front
xylR
Pxyl
MCS
amyE back
Primer set
Flagment D
SMP07
SMP08
Flagment H
SMP09
SMP17
rpoA
rplQ
Flagment I
SMP11
SMP12
rpoA^del
Flagment F
SMP13
SMP14
Flagment G
SMP15
SMP16
Km
recombinant PCR
PCR product
SMS03 Chromosome
SMS04
LY111 Chromosome
tet
Fragment D
26bp complementary sequence
Fragment J
SMP18
SMP12
25bp complementary sequence
Fragment F
Fragment G
SMS04 Chromosome

S12-2 Fig.

SMS14

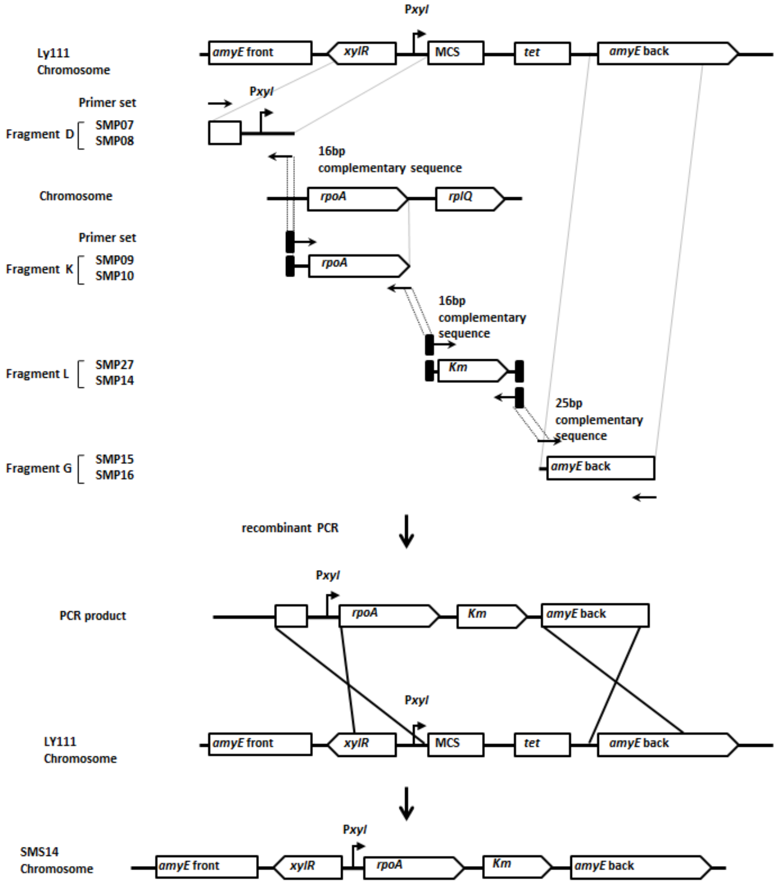

SMS15

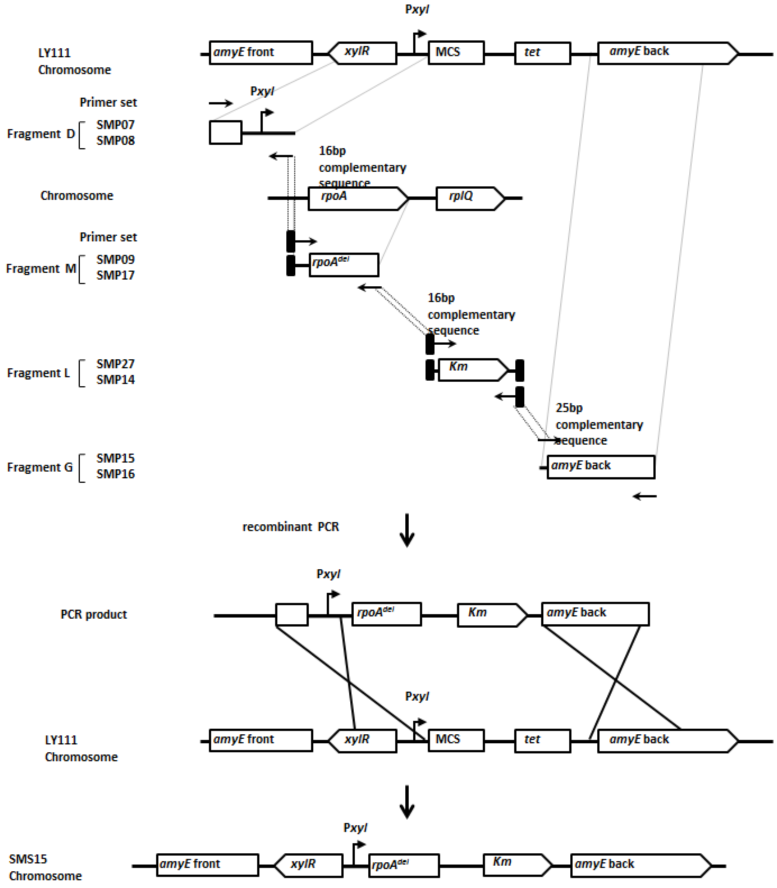

S12-3 Fig.

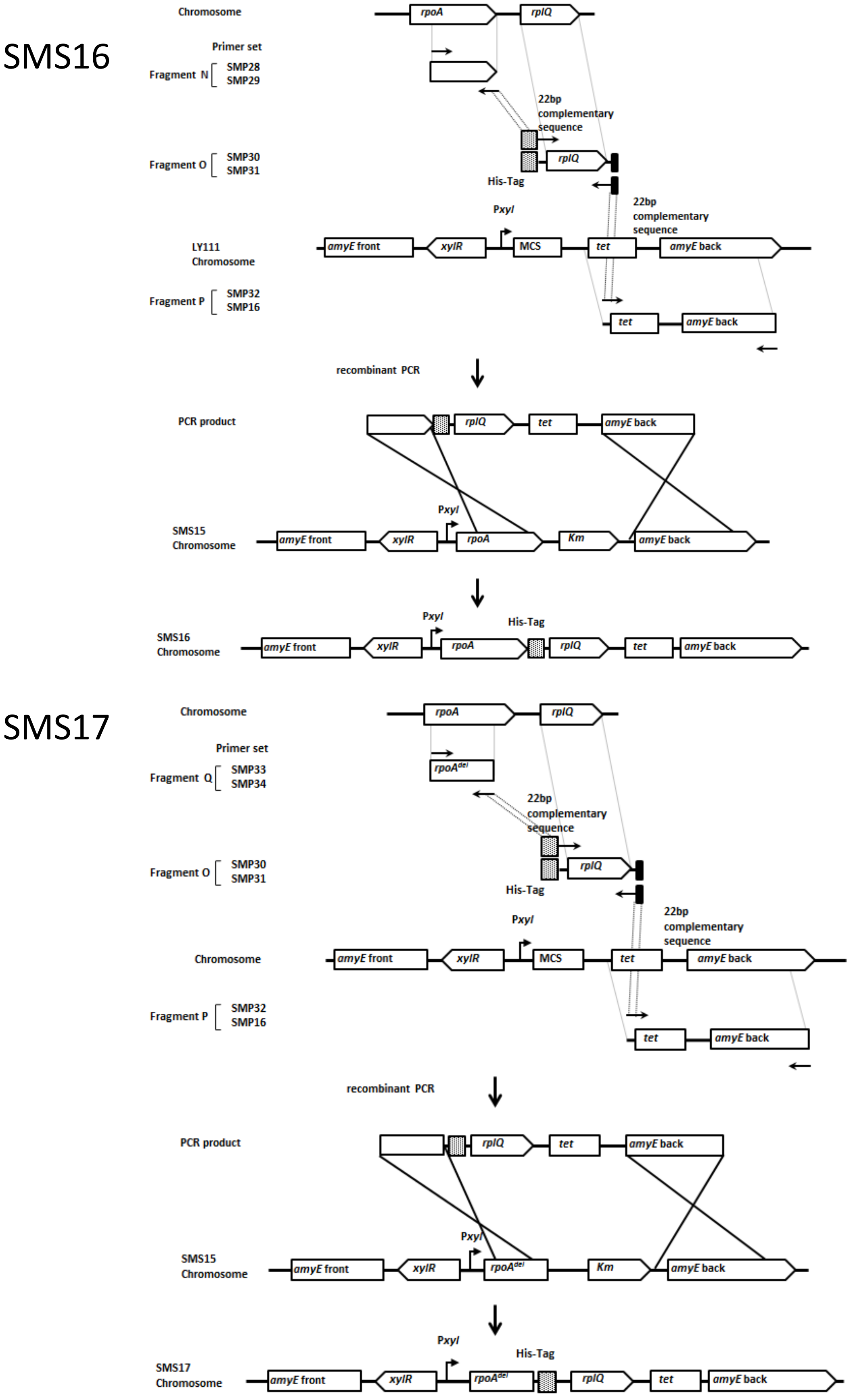
SMS16
Chromosome
rpoA
rplQ
Primer set
Fragment N
SMP28
SMP29
22bp complementary sequence
Fragment O
SMP30
SMP31
His-Tag
Pxyl
LY111 Chromosome
amyE front
xylR
MCS
tet
amyE back
Fragment P
SMP32
SMP16
recombinant PCR
PCR product
SMS15 Chromosome
Km
SMS16 Chromosome
SMS17
Fragment Q
SMP33
SMP34
rpoA^del
SMS17 Chromosome

S12-4 Fig.

S12. Fig. Recombinant PCR procedures used to construct the utilized strains. Schematic representation of the primers used to amplify *B. subtilis* chromosomal and plasmid sequences (fragments A to P). Black bars indicate the complementary primer sequences used for recombinant PCR.
